# Novel Synthetic Chalcones Display Potent Anti-*Leishmania* (*Viannia*) *braziliensis* Profile and Inhibit Proteolytic Activities

**DOI:** 10.1021/acsomega.6c06140

**Published:** 2026-09-29

**Authors:** Edgar Schaeffer, Geovane Dias-Lopes, Patrícia Queiroz Monteiro, Julia Maria Borges, Luzia Monteiro de Castro Côrtes, Alcides José Monteiro da Silva, Franklin Souza da Silva, Carlos Roberto Alves

**Affiliations:** † UFRJ, Instituto de Pesquisas de Produtos Naturais Walter Mors, Laboratório de Catálise Orgânica, 28125 Universidade Federal do Rio de Janeiro, Av. Carlos Chagas Filho 373—Bloco H, Cidade Universitária, Rio de Janeiro CEP 21941-599, Brasil; ‡ Departamento de Ciências Biomédicas e Saúde, Instituto de Biologia Roberto Alcântara Gomes, 28130 Universidade do Estado do Rio de Janeiro, UERJ, Rua Arízio Gomes da Costa 186, Jardim Flamboyant, Cabo Frio, Rio de Janeiro CEP 28905-320, Brasil; § FIOCRUZ, Instituto Oswaldo Cruz, Laboratório de Biologia Molecular e Doenças Endêmicas, 37903 Fundação Oswaldo Cruz, Avenida Brasil 4365, Manguinhos, Rio de Janeiro CEP 21040-900, Brasil; ∥ UNIG, Laboratório de Pesquisas Pré-clínicas, Universidade Iguaçu, Avenida Abílio Augusto Távora 2134, Dom Rodrigo, Nova Iguaçu, Rio de Janeiro CEP 26260-045, Brasil; ⊥ Centro de Desenvolvimento Tecnológico em Saúde, Fundação Oswaldo Cruz, FIOCRUZ, Avenida Brasil, 4036—Manguinhos, Rio de Janeiro CEP 21040-361, Brasil

## Abstract

American Tegumentary Leishmaniasis, primarily caused by *Leishmania* (*Viannia*) *braziliensis*, remains a major public health challenge in Latin America. The variable efficacy and toxicity of pentavalent antimonials underscore the urgent need for new therapeutic candidates. Among the compounds currently explored, chalcones have emerged as promising bioactive scaffolds, exhibiting antiparasitic activity and the potential to inhibit key proteases, which are essential enzymes to virulence and pathogenesis of the parasite. In this study, chalcone derivatives obtained via a novel synthetic approach were evaluated for their antileishmanial activity against *L.*­(*V.*)­*braziliensis*. Their drug-likeness properties were initially assessed in silico, and the inhibitory activity against proteases was subsequently evaluated using promastigote protein extracts. In Tris–HCl buffer, compound **3j** exhibited the most pronounced inhibitory effect, 36% residual activity, followed by compounds **3d** and 2′,4′,6′-trimethoxy-chalcone (**TMC**), which showed 47% residual activity. In acetate buffer, the most effective inhibitors were **3b** and **3j** (34%), and **3d** (35%). Cytotoxicity assays were performed in murine macrophages, and the leishmanicidal effects were evaluated against promastigotes to determine compound selectivity index (SI). **TMC**, **3b**, **3d**, **3h**, and **3i** exhibited SI ≥ 16.74 and were tested against intracellular amastigotes. They decreased ≥34% macrophage infection by intracellular amastigotes, and demonstrated a predictive profile for high metabolic stability with low organ toxicity risk. The findings suggest that these chalcones may act through a multitarget mechanism involving the inhibition of parasite proteases, supporting their progression to advanced stages of therapeutic validation.

## Graphical abstract

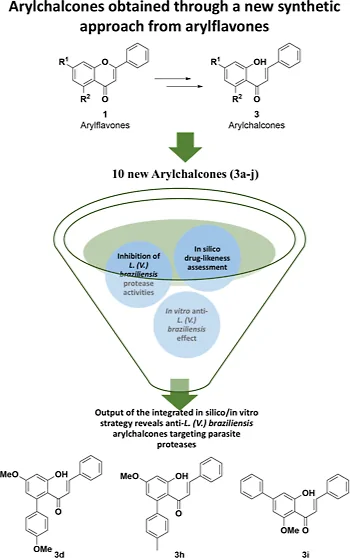

## 1 Introduction

American Tegumentary Leishmaniasis (ATL), caused primarily by species of the*Viannia* subgenus, such as *Leishmania* (*Viannia*) *braziliensis*, remains a serious public health problem in endemic regions of Latin America.^1–3^ In Brazil, epidemiological data from the national surveillance panel indicate that ATL remains endemic, with persistent transmission and annual fluctuations in the number of reported cases over the past decades (https://leishmanioses.aids.gov.br/—accessed on March 18, 2026).

In this epidemiological context, the therapeutic management remains a major challenge. Treatment options for this disease are still limited to a small number of drugs, with pentavalent antimonials constituting first-line therapy. However, their efficacy is variable and frequently associated with significant side effects and the emergence of resistant strains.^4,5^ Given these challenges, there is an urgent need for new antileishmanial agents capable of eliminating the parasite while maintaining safety, affordability, and efficacy.

Considering this therapeutic scenario, parasitic proteases, particularly cysteine and serine proteases, have emerged as attractive targets due to their critical roles in parasite survival, pathogenesis, and immune evasion in *Leishmania* spp.^6–8^ The genes encoding these proteases are highly conserved, and the corresponding enzymes represent promising targets for antileishmanial drug development.^9,10^ The diversity and molecular characteristics of these proteases reinforces their potential as drug targets, particularly in the search for novel therapeutic agents based on bioactive compounds such as chalcones. This interest is further supported by evidence showing that these compounds can inhibit different classes of proteolytic enzymes, reinforcing their relevance as promising candidates for drug development.^11–13^

Chalcones are naturally occurring organic molecules that have emerged as attractive scaffolds in the development of new therapeutic agents for a variety of diseases, including cancer, leishmaniasis, and other parasitic infections.^14–17^ Evidence indicates that chalcone derivatives exhibit leishmanicidal activity against both promastigote and amastigote forms of *Leishmania* spp.; however, despite their recognized antileishmanial potential, the molecular mechanisms underlying these effects remain incompletely understood, particularly regarding their specific intracellular targets and the pathways involved in parasite death.^18^

Their synthetic versatility allows for extensive molecular modification, enabling the design of derivatives with an improved selectivity and potency. This has supported their proposed multitarget potential against *L.*­(*V.*)­*braziliensis* proteases.^19^ These attributes highlight synthetic chalcones as promising agents for targeting *Leishmania* parasites and support the rational development of novel therapeutic strategies targeting parasite-specific proteases. However, there is still limited evidence regarding derivatives specifically designed to inhibit essential proteases involved in parasite physiology, particularly in *L.*­(*V.*)­*braziliensis*, a species of major clinical and epidemiological relevance in South America. This gap reinforces the need for studies focused on protease inhibition as a targeted therapeutic approach to this parasite.

To further investigate the inhibitory potential of chalcones previously evaluated in silico against cysteine and serine proteases,^19^ a new synthetic approach was developed, and the resulting chalcone derivatives were evaluated for their leishmanicidal activity using an integrated strategy combining drug-likeness assessment and experimental validation. The inhibitory capacity of molecules was validated in vitro by using promastigote protein extracts under specific conditions for both protease classes. Furthermore, the biological efficacy and selectivity of these compounds were assessed against promastigotes and intracellular amastigotes within murine macrophages alongside cytotoxicity assays. Overall, the results suggest that these novel chalcones may act as parasite protease inhibitors, exhibiting potent antileishmanial activity and providing a molecular rationale for their therapeutic potential.

## 2 Materials and Methods

### 2.1 Ethics Statement and Regulatory Compliance

Access to the *L.*­(*V.*)­*braziliensis* Thor strain (MCAN/BR/1998/R619) was registered in the Brazilian System of Genetic Resource Management and Associated Traditional Knowledge (SisGen A41DBDD - approved on October 16, 2018). Animal assays were approved by the Ethics Committee on Animal Use of the Oswaldo Cruz Institute (CEUA-IOC: L-014/2023A1 - approved on February 06, 2025). The Laboratory of Molecular Biology and Endemic Diseases of the Oswaldo Cruz Institute (IOC/Fiocruz) holds a Biosafety Quality Certificate (CQB 105/99 - approved on November 21, 2025), authorizing operations at Biosafety Level 2 (BSL-2).

### 2.2 In Silico Pharmacokinetic and Toxicological Evaluations

The in silico approach aimed to predict potential interactions between the chalcone derivatives and key *L.*­(*V.*)­*braziliensis* proteases. Initially, the chemical structures of the selected chalcones were drawn using ChemDraw (https://revvitysignals.com/products/research/chemdraw) and converted to three-dimensional formats. Molecular geometry optimization was performed by using the MMFF94 force field in Avogadro to generate energetically favorable conformations.

The evaluation of pharmacokinetic and safety parameters for the synthesized compounds was performed using the ProTox 3.0 web server (https://tox.charite.de/protox3/, accessed on 05 April 2025). The server operates via a hybrid, multilayered computational framework that integrates machine learning algorithms, fragment-based structural similarity, and protein–ligand pharmacophore mapping. Input structures were processed from canonical SMILES codes. The structures were evaluated based on balanced accuracy, sensitivity, specificity, and the Area Under the ROC Curve within the structures’ applicability domain.^20^

On the basis of the determination of the 2D structures of the chalcones in silico, pharmacokinetic and toxicological evaluations were performed. Key physicochemical properties related to drug-likeness, including molecular weight (MW), lipophilicity (LogP), hydrogen bond donors and acceptors, and topological polar surface area (TPSA), were predicted to support the early evaluation of the chalcone derivatives. Together, these parameters helped identify compounds with physicochemical characteristics compatible with a favorable pharmacokinetic behavior.

In addition, parameters related to acute toxicity and interactions with toxicity-associated targets were assessed, including organ toxicity, acute toxicity end points (LD_50_), Tox21 nuclear receptor signaling pathways, Tox21 stress response pathways, molecular initiating events, and metabolism, using ProTox-3.0 server. As chalcones are well represented within the chemical space used to develop these models, this platform is suitable for their preliminary toxicological evaluation. Although these predictions require experimental confirmation, they are useful for prioritizing compounds with more favorable absorption, distribution, metabolism, excretion, and toxicity (ADMET) profiles during the early stages of drug discovery.

### 2.3 Compounds

All reagents and solvents were purchased from Sigma-Aldrich/Merck (St. Louis, MO, USA) and Tokyo Chemical Industry Co., Ltd. (TCI, Tokyo, Japan) and were used without further purification unless otherwise noted. Melting points were obtained on a Stanford Research Systems OptiMelt MPA100 apparatus and are uncorrected. NMR (nuclear magnetic resonance) spectra were recorded in deuterated chloroform, with tetramethylsilane as the internal standard. The samples were analyzed at 400 or 500 MHz ^1^H NMR, 101 or 126 MHz ^13^C APT (Attached Proton Test) NMR using Varian NMR spectrometers. Chemical shifts are reported as δ values (ppm). The ^13^C APT NMR experiment yields quaternary (C) and methylene (CH_2_) signals positive and methine (CH) and methyl (CH_3_) signals negative. Data are reported as follows: chemical shift (multiplicity, coupling constant(s), integration). Multiplicity reported according to standard abbreviations. High-resolution mass spectra were obtained using a hybrid Quadrupole-Time-of-Flight mass spectrometer with high resolution and mass accuracy (Waters Corporation, Xevo G2 QTof, Milford, MA, USA) equipped with an electrospray ionization source.

The progress of reactions was monitored by thin-layer chromatography (TLC), and flash column chromatography was carried out on silica gel 60, 230–400 mesh (SiliCycle-Quebec City, QC, Canada). Analytical TLC was performed using precoated TLC sheets of silica gel 60 F254 (SiliCycle), and the spots were determined under UV light (λ = 254 and 365 nm). Compounds **1a–j** were synthesized as described previously.^21^ Pinostrobin chalcone (**3a**) and flavokawain B (**3b**) were synthesized as described below, while chalcone 2′,4′,6′-trimethoxy-chalcone (TMC), a known compound, was obtained through methylation of **3b** (Figure S1 and S46).^22^

### 2.4 General Synthetic Route

The synthesis of the chalcones **3a–j** is summarized in Figure 1. In the first step, some modifications of standard methodologies for flavone C2–C3 double bond reduction were employed for the synthesis of flavanones **2a–j**, which included an increase in the amount of the palladium catalyst to 40 mol % and changing the palladium source.^23–25^ These key intermediates **2a–j** were subjected to a newly developed ring opening approach, which was employed for the synthesis of chalcones **3a–j**, through strong basic conditions (potassium *tert*-butoxide-dimethyl sulfoxide: *t*-BuOK-DMSO)^26^ (Figure 1).

**Figure 1:**
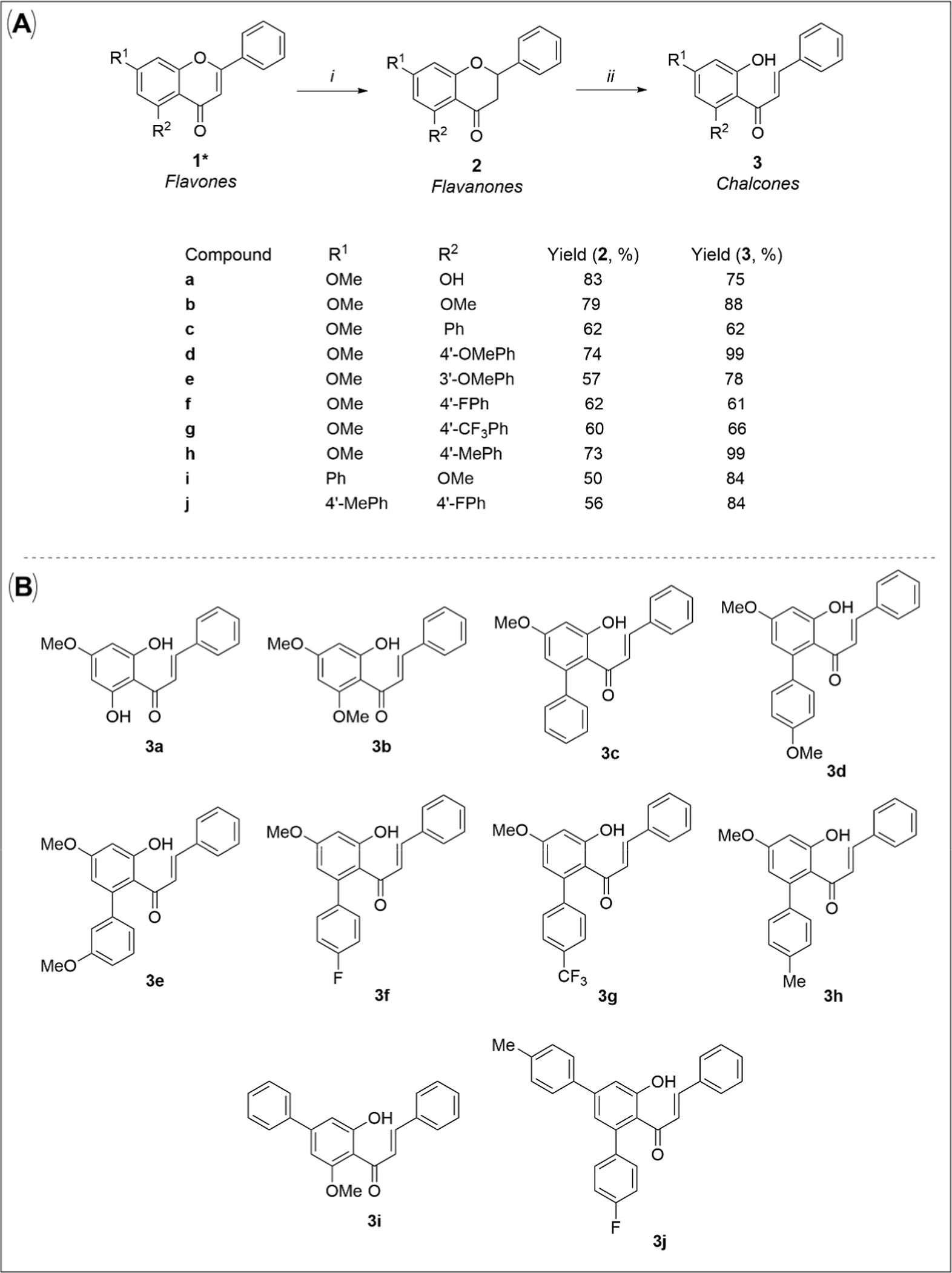
Synthetic route for the preparation of arylchalcones from the reported arylflavones. (A) General synthetic pathway from arylflavones to arylchalcones via arylflavanone intermediates. Reagents and conditions: (i) Pd/C (10%) or Pd­(OH)_2_/C (20%), H_2_ (1 atm), EtOAc, room temperature, 12 h; (ii) *t*-BuOK (4 equiv), DMSO, room temperature, 15 min. The percentages shown correspond to the isolated yields obtained for each reaction step: flavone → flavanone (i) and flavanone → chalcone (ii). (B) Structures of the synthesized arylchalcone derivatives (**3a–3j**). (*): Arylflavones were previously synthesized and reported.^21^

#### 2.4.1 General Procedures for the Reduction of Flavones (**1a–j**)

To an ethyl acetate (1.25 mL) flavone solution (0.25 mmol) was added the required amount of a suitable palladium catalyst. This mixture was degassed with hydrogen and kept stirring at room temperature under a hydrogen atmosphere (1 atm) for 12 h. The reaction progress was monitored by the TLC. The reactional mixture was filtered through a pad of Celite, eluting with ethyl acetate (AcOEt), and concentrated under reduced pressure. The pure product was obtained using flash column chromatography on silica gel (eluting with 2:8 EtOAc/hexanes).

#### 2.4.2 General Procedure for the Synthesis of Flavanones (**2a–j**)

Subjection of the previously obtained flavones **1a–j** on optimized reduction conditions (general procedure 2.4.1) furnished the flavanones **2a–j** in moderate to good yields (50–83%). These new compounds were thoroughly characterized by spectrometric and spectroscopic techniques, such as HRMS (Figures S2–S9), ^1^H NMR, and ^13^C APT NMR (Figures S26–S35) as described below.

##### 2.4.2.1 5-Hydroxy-7-methoxy-2-phenylchroman-4-one (**2a**)

Pd/C 10% (10 mol %) and 36 h reaction time were employed. Yield: 83%; white solid. ^1^H NMR (500 MHz, acetone-*d*_6_) δ: 12.12 (s, 1H), 7.56 (d, *J* = 7.6 Hz, 2H), 7.42 (dt, *J* = 26.2, 7.2 Hz, 3H), 6.07 (dd, *J* = 16.8, 2.3 Hz, 2H), 5.58 (dd, *J* = 12.8, 3.1 Hz, 1H), 3.84 (s, 3H), 3.18 (dd, *J* = 17.1, 12.8 Hz, 1H), 2.83 (dd, *J* = 17.1, 3.1 Hz, 1H). ^13^C APT NMR (126 MHz, acetone-*d*_6_) δ: 196.31, 168.01, 164.14, 163.07, 139.05, 128.62, 128.57, 126.41, 102.88, 94.72, 93.79, 79.15, 55.38, 42.73. The structural assignment of the product coincides with the previous report^24^ (Figure S26).

##### 2.4.2.2 5,7-Dimethoxy-2-phenylchroman-4-one (**2b**)

Pd/C 10% (10 mol %) and 30 h reaction time were employed. Yield: 79%; white solid. ^1^H NMR (500 MHz, CDCl_3_) δ: 7.48–7.35 (m, 5H), 6.16 (d, *J* = 2.3 Hz, 1H), 6.10 (d, *J* = 2.3 Hz, 1H), 5.41 (dd, *J* = 13.2, 2.9 Hz, 1H), 3.90 (s, 3H), 3.82 (s, 3H), 3.02 (dd, *J* = 16.5, 13.2 Hz, 1H), 2.80 (dd, *J* = 16.5, 2.9 Hz, 1H). ^13^C APT NMR (126 MHz, CDCl_3_) δ: 189.20, 165.98, 164.98, 162.29, 138.76, 128.78, 128.66, 126.11, 105.99, 93.55, 93.18, 79.22, 56.16, 55.60, 45.59. The structural assignment of the product coincides with the previous report^25^ (Figure S27).

##### 2.4.2.3 7-Methoxy-2,5-diphenylchroman-4-one (**2c**)

Pd­(OH)_2_/C 20% (40 mol %) was employed. Yield: 62%; white solid; Mp: 124.7 °C. ^1^H NMR (500 MHz, CDCl_3_) δ: 7.50–7.47 (m, 2H), 7.45–7.41 (m, 2H), 7.40–7.34 (m, 4H), 7.30–7.27 (m, 2H), 6.54 (d, *J* = 2.5 Hz, 1H), 6.45 (d, *J* = 2.6 Hz, 1H), 5.51 (dd, *J* = 13.4, 2.9 Hz, 1H), 3.83 (s, 3H), 3.00 (dd, *J* = 16.8, 13.4 Hz, 1H), 2.75 (dd, *J* = 16.9, 3.0 Hz, 1H). ^13^C APT NMR (126 MHz, CDCl_3_) δ: 189.79, 164.70, 164.20, 146.45, 141.66, 138.81, 128.85, 128.76, 128.20, 127.72, 127.20, 126.18, 113.40, 112.82, 100.63, 79.43, 55.68, 45.54. HRMS (ESI) calcd for C_22_H_18_NaO_3_ [(M + Na)^+^]: 353.1154, found 353.1153 (Figures S2 and S28).

##### 2.4.2.4 7-Methoxy-5-(4-methoxyphenyl)-2-phenylchroman-4-one (**2d**)

Pd­(OH)_2_/C 20% (40 mol %) was employed. Yield: 74%; white solid; Mp: 139.5 °C. ^1^H NMR (400 MHz, CDCl_3_) δ: 7.51–7.35 (m, 5H), 7.25–7.21 (m, 2H), 6.96–6.88 (m, 2H), 6.52 (d, *J* = 2.6 Hz, 1H), 6.44 (d, *J* = 2.5 Hz, 1H), 5.51 (dd, *J* = 13.4, 2.9 Hz, 1H), 3.84 (d, *J* = 4.3 Hz, 6H), 3.00 (dd, *J* = 16.9, 13.4 Hz, 1H), 2.76 (dd, *J* = 16.8, 2.9 Hz, 1H). ^13^C APT NMR (101 MHz, CDCl_3_) δ: 189.89, 164.82, 164.17, 158.97, 146.22, 138.81, 133.85, 129.49, 128.83, 128.72, 126.16, 113.46, 113.18, 112.83, 100.35, 79.31, 55.62, 55.22, 45.59. HRMS (ESI) calcd for C_23_H_20_NaO_4_ [(M + Na)^+^]: 383.1259, found 383.1252 (Figures S3 and S29).

##### 2.4.2.5 7-Methoxy-5-(3-methoxyphenyl)-2-phenylchroman-4-one (**2e**)

Pd­(OH)_2_/C 20% (40 mol %) was employed. Yield: 57%; thick liquid. ^1^H NMR (500 MHz, CDCl_3_) δ: 7.50–7.36 (m, 5H), 7.31–7.27 (m, 1H), 6.91–6.82 (m, 3H), 6.54 (d, *J* = 2.5 Hz, 1H), 6.46 (d, *J* = 2.6 Hz, 1H), 5.51 (dd, *J* = 13.4, 2.9 Hz, 1H), 3.83 (d, *J* = 7.5 Hz, 6H), 3.00 (dd, *J* = 16.8, 13.4 Hz, 1H), 2.75 (dd, *J* = 16.8, 2.9 Hz, 1H). ^13^C APT NMR (126 MHz, CDCl_3_) δ: 189.62, 164.63, 164.17, 159.09, 146.17, 143.07, 138.81, 128.85, 128.75, 128.65, 126.17, 120.80, 114.05, 113.25, 112.86, 112.48, 100.70, 79.44, 55.68, 55.20, 45.53. HRMS (ESI) calcd for C_23_H_20_NaO_4_ [(M + Na)^+^]: 383.1259, found 383.1258 (Figures S4 and S30).

##### 2.4.2.6 5-(4-Fluorophenyl)-7-methoxy-2-phenylchroman-4-one (**2f**)

Pd­(OH)_2_/C 20% (40 mol %) was employed. Yield: 62%; white solid; Mp: 123.3 °C. ^1^H NMR (400 MHz, CDCl_3_) δ: 7.53–7.35 (m, 5H), 7.27–7.21 (m, 2H), 7.10–7.02 (m, 2H), 6.55 (d, *J* = 2.5 Hz, 1H), 6.41 (d, *J* = 2.5 Hz, 1H), 5.51 (dd, *J* = 13.4, 2.9 Hz, 1H), 3.84 (s, 3H), 3.00 (dd, *J* = 16.8, 13.4 Hz, 1H), 2.75 (dd, *J* = 16.9, 3.0 Hz, 1H). ^13^C APT NMR (101 MHz, CDCl_3_) δ: 189.87, 164.80, 164.23, 162.23 (d, *J* = 245.7 Hz), 145.30, 138.68, 137.42 (d, *J* = 3.4 Hz), 129.86 (d, *J* = 7.8 Hz), 128.86, 128.79, 126.15, 114.62 (d, *J* = 21.5 Hz), 113.62 (d, *J* = 2.9 Hz), 112.71, 100.65, 79.39, 55.66, 45.47. HRMS (ESI) calcd for C_22_H_17_FNaO_3_ [(M + Na)^+^]: 371.1059, found 371.1059 (Figures S5 and S31).

##### 2.4.2.7 7-Methoxy-2-phenyl-5-(4-(trifluoromethyl)­phenyl)­chroman-4-one (**2g**)

Pd­(OH)_2_/C 20% (40 mol %) was employed. Yield: 60%; white solid; Mp: 172.5 °C. ^1^H NMR (500 MHz, CDCl_3_) δ: 7.63 (dt, *J* = 7.9, 0.7 Hz, 2H), 7.50–7.37 (m, 7H), 6.58 (d, *J* = 2.5 Hz, 1H), 6.41 (d, *J* = 2.5 Hz, 1H), 5.52 (dd, *J* = 13.4, 2.9 Hz, 1H), 3.85 (s, 3H), 3.01 (dd, *J* = 16.9, 13.4 Hz, 1H), 2.76 (dd, *J* = 16.9, 3.0 Hz, 1H). ^13^C APT NMR (126 MHz, CDCl_3_) δ: 189.80, 164.81, 164.38, 145.37 (d, *J* = 1.5 Hz), 144.74, 138.57, 129.23 (d, *J* = 32.3 Hz), 128.89, 128.86, 128.56, 126.16, 124.67 (q, *J* = 3.7 Hz), 124.36 (d, *J* = 272.1 Hz), 113.54, 112.53, 101.01, 79.51, 55.75, 45.30. HRMS (ESI) calcd for C_23_H_17_F_3_NaO_3_ [(M + Na)^+^]: 421.1027, found 421.1037 (Figures S6 and S32).

##### 2.4.2.8 7-Methoxy-2-phenyl-5-(*p*-tolyl)­chroman-4-one (**2h**)

Pd­(OH)_2_/C 20% (40 mol %) was employed. Yield: 73%; white solid; Mp: 140.6 °C. ^1^H NMR (500 MHz, CDCl_3_) δ: 7.50–7.36 (m, 5H), 7.19 (s, 4H), 6.53 (d, *J* = 2.6 Hz, 1H), 6.45 (d, *J* = 2.5 Hz, 1H), 5.51 (dd, *J* = 13.4, 2.9 Hz, 1H), 3.84 (s, 3H), 3.00 (dd, *J* = 16.8, 13.4 Hz, 1H), 2.75 (dd, *J* = 16.8, 2.9 Hz, 1H), 2.40 (s, 3H). ^13^C APT NMR (126 MHz, CDCl_3_) δ: 189.83, 164.73, 164.17, 146.55, 138.84, 138.69, 136.88, 128.84, 128.73, 128.48, 128.12, 126.17, 113.36, 112.90, 100.50, 79.39, 55.65, 45.59, 21.32. HRMS (ESI) calcd for C_23_H_20_NaO_3_ [(M + Na)^+^]: 367.131, found 367.1306 (Figures S7 and S33).

##### 2.4.2.9 5-Methoxy-2,7-diphenylchroman-4-one (**2i**)

Pd/C 10% (40 mol %) was employed. Yield: 50%; white solid; Mp: 170.5 °C. ^1^H NMR (500 MHz, CDCl_3_) δ: 7.62–7.58 (m, 2H), 7.50–7.36 (m, 8H), 6.91 (d, *J* = 1.6 Hz, 1H), 6.75 (d, *J* = 1.6 Hz, 1H), 5.48 (dd, *J* = 13.2, 2.9 Hz, 1H), 4.00 (s, 3H), 3.09 (dd, *J* = 16.5, 13.2 Hz, 1H), 2.88 (dd, *J* = 16.5, 2.9 Hz, 1H). ^13^C APT NMR (126 MHz, CDCl_3_) δ: 190.35, 160.99, 149.01, 139.74, 138.75, 128.95, 128.82, 128.79, 128.70, 127.14, 126.13, 110.15, 108.79, 103.17, 79.09, 56.31, 45.94. HRMS (ESI) calcd for C_22_H_18_NaO_3_ [(M + Na)^+^]: 353.1154, found 353.1152 (Figures S8 and S34).

##### 2.4.2.10 5-(4-Fluorophenyl)-2-phenyl-7-(*p*-tolyl)­chroman-4-one (**2j**)

Pd­(OH)_2_/C 20% (40 mol %) and an ultrasonic bath (40–45 °C average temperature) were employed. Yield: 56%; white solid; Mp: 145.6 °C. ^1^H NMR (500 MHz, CDCl_3_) δ: 7.56–7.49 (m, 4H), 7.47–7.43 (m, 2H), 7.39 (td, *J* = 7.4, 1.5 Hz, 1H), 7.33–7.24 (m, 5H), 7.12–7.04 (m, 3H), 5.56 (dd, *J* = 13.4, 2.9 Hz, 1H), 3.08 (ddd, *J* = 16.7, 13.4, 1.2 Hz, 1H), 2.86–2.80 (m, 1H), 2.39 (s, 3H). ^13^C APT NMR (126 MHz, CDCl_3_) δ: 191.02, 163.04, 162.26 (d, *J* = 246.0 Hz), 147.30, 143.85, 138.95, 138.70, 137.63 (d, *J* = 3.3 Hz), 136.01, 130.06 (d, *J* = 8.0 Hz), 129.75, 128.89, 128.82, 127.04, 126.17, 123.90, 117.27, 115.42, 114.70 (d, *J* = 21.5 Hz), 79.23, 45.80, 21.22. HRMS (ESI) calcd for C_28_H_21_FO_2_ [(M + Na)^+^]: 431.1423, found 431.1423 (Figures S9 and S35).

#### 2.4.3 General Procedure for the Synthesis of Chalcones (**3a–j**)

To a solution of flavanone (0.25 mmol) in DMSO (1.25 mL), potassium *t*-butoxide (1 mmol, 4 equiv) was added. This mixture was kept stirring at room temperature for 15 min, diluted with ethyl acetate (20 mL) and washed with brine (3 × 30 mL). The organic layer was dried over anhydrous sodium sulfate, and concentrated under reduced pressure to furnish the crude product. The residue obtained was purified by flash column chromatography using hexanes-ethyl acetate (10%) as the eluent to afford the pure chalcone.

Taking the next and last step of the synthetic route, these conveniently synthesized flavanones **2a–j** were subjected to newly developed ring-opening conditions affording the chalcones **3a–j** in moderate to excellent yield (61–99%) (Figure 1). These compounds were equally fully characterized by spectrometric (HRMS-Figures S10–S17) and spectroscopy techniques (^1^H and ^13^C APT NMR-Figures S36–S45), and its purity level was assessed by HPLC-UV analysis (Figures S18–S25), as shown below.

##### 2.4.3.1 (*E*)-1-(2,6-Dihydroxy-4-methoxyphenyl)-3-phenylprop-2-en-1-one (**3a/Pinostrobin chalcone**)

Yield: 75%; yellow crystals. ^1^H NMR (500 MHz, acetone-*d*_6_) δ: 7.38 (d, *J* = 15.6 Hz, 1H), 6.92 (d, *J* = 15.6 Hz, 1H), 6.84–6.80 (m, 2H), 6.57 (dd, *J* = 8.2, 2.3 Hz, 3H), 5.17 (s, 2H), 2.94 (s, 3H). ^13^C APT NMR (126 MHz, acetone-*d*_6_) δ: 205.48, 192.61, 166.39, 164.52, 142.01, 135.61, 130.13, 128.97, 128.32, 127.59, 126.43, 93.65, 55.00. The structural assignment of the product coincides with the previous report^27^ (Figure S36).

##### 2.4.3.2 (*E*)-1-(2-Hydroxy-4,6-dimethoxyphenyl)-3-phenylprop-2-en-1-one (**3b/Flavokawain B**)

Yield: 88%; yellow crystals. ^1^H NMR (400 MHz, CDCl_3_) δ: 14.29 (s, 1H), 7.90 (d, *J* = 15.6 Hz, 1H), 7.78 (d, *J* = 15.6 Hz, 1H), 7.62–7.58 (m, 2H), 7.44–7.35 (m, 3H), 6.11 (d, *J* = 2.3 Hz, 1H), 5.96 (d, *J* = 2.4 Hz, 1H), 3.91 (s, 3H), 3.83 (s, 3H). ^13^C APT NMR (101 MHz, CDCl_3_) δ: 192.64, 168.40, 166.25, 162.51, 142.31, 135.57, 130.05, 128.87, 128.35, 127.54, 106.35, 93.79, 91.27, 55.86, 55.58. The structural assignment of the product coincides with the previous report^28^ (Figure S37).

##### 2.4.3.3 (*E*)-1-(3-Hydroxy-5-methoxy-[1,1′-biphenyl]-2-yl)-3-phenylprop-2-en-1-one (**3c**)

Yield: 62%; yellow crystals; Mp: 112.8 °C. ^1^H NMR (500 MHz, CDCl_3_) δ: 12.85 (s, 1H), 7.50 (d, *J* = 15.5 Hz, 1H), 7.45–7.35 (m, 5H), 7.28–7.20 (m, 3H), 6.98–6.95 (m, 2H), 6.50 (dd, *J* = 16.0, 2.6 Hz, 2H), 6.28 (d, *J* = 15.5 Hz, 1H), 3.88 (s, 3H). ^13^C APT NMR (126 MHz, CDCl_3_) δ: 194.36, 165.75, 163.98, 146.35, 142.02, 140.95, 134.91, 130.05, 129.36, 128.80, 128.58, 128.33, 128.19, 126.95, 115.06, 110.79, 100.04, 55.62. HRMS (ESI) calcd for C_22_H_18_NaO_3_ [(M + Na)^+^]: 353.1154, found 353.1155 (Figures S10, S18, and S38).

##### 2.4.3.4 (*E*)-1-(3-Hydroxy-4′,5-dimethoxy-[1,1′-biphenyl]-2-yl)-3-phenylprop-2-en-1-one (**3d**)

Yield: 99%; yellow crystals; Mp: 104.9 °C. ^1^H NMR (500 MHz, CDCl_3_) δ: 12.82 (s, 1H), 7.49 (d, *J* = 15.5 Hz, 1H), 7.33–7.25 (m, 3H), 7.23 (ddt, *J* = 8.5, 6.7, 1.5 Hz, 2H), 7.03–6.99 (m, 2H), 6.96–6.91 (m, 2H), 6.51–6.44 (m, 2H), 6.29 (d, *J* = 15.6 Hz, 1H), 3.87 (s, 3H), 3.76 (s, 3H). ^13^C APT NMR (126 MHz, CDCl_3_) δ: 194.51, 165.67, 164.02, 159.95, 146.13, 140.59, 135.06, 134.28, 130.65, 130.01, 128.57, 128.15, 127.15, 115.20, 114.27, 110.43, 99.74, 55.59, 55.42. HRMS (ESI) calcd for C_23_H_20_NaO_4_ [(M + Na)^+^]: 383.1259, found 383.1253 (Figures S11, S19, and S39).

##### 2.4.3.5 (*E*)-1-(3-Hydroxy-3′,5-dimethoxy-[1,1′-biphenyl]-2-yl)-3-phenylprop-2-en-1-one (**3e**)

Yield: 78%; yellow crystals; Mp: 90.9 °C. ^1^H NMR (500 MHz, CDCl_3_) δ: 12.86 (s, 1H), 7.51 (d, *J* = 15.5 Hz, 1H), 7.35–7.31 (m, 1H), 7.29–7.26 (m, 1H), 7.25–7.20 (m, 2H), 7.03–6.98 (m, 3H), 6.95 (dd, *J* = 2.6, 1.6 Hz, 1H), 6.91 (ddd, *J* = 8.2, 2.6, 0.9 Hz, 1H), 6.51–6.48 (m, 2H), 6.35 (d, *J* = 15.5 Hz, 1H), 3.87 (s, 3H), 3.76 (s, 3H). ^13^C APT NMR (126 MHz, CDCl_3_) δ: 194.35, 165.74, 163.97, 159.89, 146.16, 143.34, 140.88, 134.99, 130.03, 129.87, 128.59, 128.20, 126.95, 121.90, 115.00, 114.98, 114.02, 110.59, 100.13, 55.62, 55.46. HRMS (ESI) calcd for C_23_H_20_NaO_4_ [(M + Na)^+^]: 383.1259, found 383.1258 (Figures S12, S20, and S40).

##### 2.4.3.6 (*E*)-1-(4′-Fluoro-3-hydroxy-5-methoxy-[1,1′-biphenyl]-2-yl)-3-phenylprop-2-en-1-one (**3f**)

Yield: 61%; yellow crystals; Mp: 137.3 °C. ^1^H NMR (500 MHz, CDCl_3_) δ: 12.78 (s, 1H), 7.51 (d, *J* = 15.5 Hz, 1H), 7.40–7.35 (m, 2H), 7.32–7.23 (m, 3H), 7.13–7.09 (m, 2H), 7.04–7.00 (m, 2H), 6.51 (d, *J* = 2.6 Hz, 1H), 6.44 (d, *J* = 2.6 Hz, 1H), 6.26 (d, *J* = 15.5 Hz, 1H), 3.88 (s, 3H). ^13^C APT NMR (126 MHz, CDCl_3_) δ: 194.21, 165.76, 164.02, 162.89 (d, *J* = 248.6 Hz), 145.03, 141.26, 137.97 (d, *J* = 3.4 Hz), 134.74, 131.03 (d, *J* = 8.1 Hz), 130.28, 128.75, 128.09, 126.75, 115.74 (d, *J* = 21.6 Hz), 115.04, 110.91, 100.08, 55.64. HRMS (ESI) calcd for C_22_H_17_FNaO_3_ [(M + Na)^+^]: 371.1059, found 371.1064 (Figures S13, S21, and S41).

##### 2.4.3.7 (*E*)-1-(3-Hydroxy-5-methoxy-4′-(trifluoromethyl)-[1,1′-biphenyl]-2-yl)-3-phenylprop-2-en-1-one (**3g**)

Yield: 66%; yellow crystals; Mp: 142.8 °C. ^1^H NMR (500 MHz, CDCl_3_) δ: 13.03 (s, 1H), 7.72–7.67 (m, 2H), 7.56–7.49 (m, 3H), 7.31–7.26 (m, 1H), 7.24–7.20 (m, 2H), 6.90–6.87 (m, 2H), 6.55 (d, *J* = 2.6 Hz, 1H), 6.45 (d, *J* = 2.6 Hz, 1H), 6.14 (d, *J* = 15.5 Hz, 1H), 3.89 (s, 3H). ^13^C APT NMR (126 MHz, CDCl_3_) δ: 193.79, 166.33, 164.09, 145.76 (d, *J* = 1.8 Hz), 144.54, 141.74, 134.54, 130.41, 130.39 (d, *J* = 32.9 Hz), 129.79, 128.71, 128.05, 126.64, 125.65 (q, *J* = 3.7 Hz), 123.96 (d, *J* = 272.6 Hz), 114.54, 111.18, 100.61, 55.70. HRMS (ESI) calcd for C_23_H_17_F_3_NaO_3_ [(M + Na)^+^]: 421.1027, found 421.1036 (Figures S14, S22, and S42).

##### 2.4.3.8 (*E*)-1-(3-Hydroxy-5-methoxy-4′-methyl-[1,1′-biphenyl]-2-yl)-3-phenylprop-2-en-1-one (**3h**)

Yield: 99%; yellow crystals; Mp: 109.5 °C. ^1^H NMR (500 MHz, CDCl_3_) δ: 12.92 (s, 1H), 7.48 (d, *J* = 15.5 Hz, 1H), 7.32–7.19 (m, 7H), 6.95 (dt, *J* = 7.0, 1.5 Hz, 2H), 6.52–6.45 (m, 2H), 6.26 (d, *J* = 15.5 Hz, 1H), 3.87 (s, 3H), 2.35 (s, 3H). ^13^C APT NMR (126 MHz, CDCl_3_) δ: 194.47, 165.84, 164.02, 146.51, 140.58, 139.15, 138.40, 135.08, 129.96, 129.42, 129.35, 128.56, 128.18, 127.16, 115.08, 110.53, 99.90, 55.59, 21.10. HRMS (ESI) calcd for C_23_H_20_NaO_3_ [(M + Na)^+^]: 367.131, found 367.1306 (Figure S15, S23, and S43).

##### 2.4.3.9 (*E*)-1-(3-Hydroxy-5-methoxy-[1,1′-biphenyl]-4-yl)-3-phenylprop-2-en-1-one (**3i**)

Yield: 84%; yellow crystals; Mp: 125.8 °C. ^1^H NMR (500 MHz, CDCl_3_) δ: 13.40 (s, 1H), 7.93 (d, *J* = 15.6 Hz, 1H), 7.84 (d, *J* = 15.6 Hz, 1H), 7.63 (dq, *J* = 6.4, 1.8 Hz, 4H), 7.50–7.38 (m, 6H), 6.87 (d, *J* = 1.7 Hz, 1H), 6.64 (d, *J* = 1.7 Hz, 1H), 4.03 (s, 3H). ^13^C APT NMR (126 MHz, CDCl_3_) δ: 194.05, 165.28, 161.24, 148.82, 143.02, 139.88, 135.40, 130.32, 128.94, 128.91, 128.74, 128.50, 127.57, 127.15, 110.80, 109.50, 100.74, 56.05. HRMS (ESI) calcd for C_22_H_18_NaO_3_ [(M + Na)^+^]: 353.1154, found 353.1158 (Figures S16, S24, and S44).

##### 2.4.3.10 (*E*)-1-(4″-Fluoro-5′-hydroxy-4-methyl-[1,1’:3′,1″-terphenyl]-4′-yl)-3-phenylprop-2-en-1-one (**3j**)

Yield: 84%; yellow crystals; Mp: 107.6 °C. ^1^H NMR (500 MHz, CDCl_3_) δ: 11.93 (s, 1H), 7.59–7.54 (m, 3H), 7.47–7.41 (m, 2H), 7.31–7.24 (m, 6H), 7.13 (ddd, *J* = 8.6, 5.6, 2.5 Hz, 3H), 7.06 (dt, *J* = 6.9, 1.5 Hz, 2H), 6.35 (d, *J* = 15.6 Hz, 1H), 2.41 (s, 3H). ^13^C APT NMR (126 MHz, CDCl_3_) δ: 195.41, 162.91 (d, *J* = 248.9 Hz), 162.50, 146.79, 143.45, 142.01, 138.78, 138.04 (d, *J* = 3.4 Hz), 136.36, 134.61, 131.23 (d, *J* = 8.1 Hz), 130.50, 129.71, 129.62, 128.81, 128.19, 127.08, 126.65, 120.94, 119.97, 115.82 (d, *J* = 21.5 Hz), 114.93, 21.23. HRMS (ESI) calcd for C_28_H_21_FO_2_ [(M + Na)^+^]: 431.1423, found 431.1422 (Figures S17, S25, and S45).

### 2.5 Parasite Culture

Promastigotes of *L.*­(*V.*)­*braziliensis* Thor strain were cultivated at 26 °C in Schneider’s insect medium pH 7.2 (Thermo Fisher Scientific, Waltham, MA, USA) supplemented with 20% (v/v) fetal bovine serum (FBS) (Gibco, Thermo Fisher Scientific, Waltham, MA, USA), and 100 IU/mL penicillin and 100 μg/mL streptomycin (Sigma-Aldrich Chemical Co., St. Louis, MO, USA).

### 2.6 Protein Extraction

Protein extracts were obtained from stationary-phase promastigotes (10^8^ parasites), as previously described.^29^ Briefly, parasites were washed by centrifugation (3000*g*, 4 °C, 10 min) in sterile cold PBS pH 7.2. Then, the pellets were resuspended in 1 mL of lysis buffer (100 mM Tris–HCl pH 8.0, 150 mM NaCl, 10% glycerol, and 0.6% Triton X-100) and subjected to 3 freeze–thaw cycles. After that, the soluble fraction was obtained by centrifugation (25,000*g*, 4 °C, 30 min) and the supernatant was stored at −20 °C until further use. The protein concentration was determined by the Pierce BCA Protein Assay Kit (Thermo Fisher Scientific, Waltham, MA, USA).

### 2.7 Protease Activity

The proteinase activity was performed as previously described.^29^ The cysteine protease and serine protease activity of the protein extract (5 μg) was assessed in activation buffers (10 mM Tris–HCl pH 7.5 - for serine proteases and 10 mM sodium acetate buffer pH 5.5 containing 1 mM dithiothreitol (DTT) - for cysteine proteases), using a specific fluorogenic peptide substrate, 1 mM of Z-Phe-Arg 7-amido-4-methylcoumarin (Z-FR-AMC). Samples were incubated at 37 °C for 90 min, and the variance in the relative fluorescence units (RFU) was monitored on a FlexStation 3 (Molecular Devices, San Jose, CA, USA).

Inhibition assays were performed by incubating protein extracts (25 °C, 5 min) with chalcones at different concentrations or with commercial inhibitors, such as 10 μM *N*-[*N*-(L-3-trans-Carboxyoxirane-2-carbonyl)-l-leucyl]-agmatine (E-64; for cysteine proteases) or 0.3 μM aprotinin (for serine proteases). The commercial inhibitors were prepared in bidistilled water, while the chalcones were previously dissolved in DMSO as 10 mM stock solutions and subsequently diluted to the desired concentrations for the assays.

The substrate cleavage rate was defined as follows: *v* = Δ*s*/Δ*t*, where v = velocity, Δ*s* = substrate concentration variation, and Δ*t* = total reaction time. In addition, 5% DMSO (v/v) was used as control. The self-degradation of the fluorescent peptide substrate was controlled throughout the assay to avoid incorrect readings. The enzymatic activity is expressed as RFU per minute per microgram of protein (RFU × min^–1^ × μg of protein^–1^). The IC_50_ values were determined by nonlinear regression analysis of log­[inhibitor] vs normalized response (%). All reagents used in the enzymatic reactions were purchased from Sigma-Aldrich Chemical Co. (St. Louis, MO, USA).

### 2.8 Activity Against Promastigotes

All compounds were dissolved previously in DMSO at a concentration of 10 mM. The half-maximal inhibitory concentration (IC_50_) was determined by AlamarBlue reduction assay as previously described.^30^ Briefly, promastigotes with 3 days of culture were seeded in 96-well plates (2 × 10^6^ per well) in Schneider’s medium supplemented with 20% of FBS (100 μL of total volume) with compound in concentrations ranging from 0.12 μM to 62.5 μM and incubated for 24 h at 26 °C. Amphotericin B (Bristol-Myers Squibb, New York, NY, USA) at concentrations ranging from 0.195 μM to 10 μM and 5% DMSO (v/v) were used as controls. Then, 10 μL of resazurin (Sigma-Aldrich Chemical Co., St. Louis, MO, USA) prepared at 0.015% was added to each well and incubated for 4 h at 26 °C. The analysis was performed using a FlexStation 3 with 560 nm excitation and a 590 nm emission filter. The IC_50_ values were determined by nonlinear regression analysis of log­[inhibitor] vs normalized response (%).

### 2.9 Cytotoxicity Assay in BALB/c Mice Macrophages

Cytotoxicity was evaluated in murine peritoneal macrophages obtained as previously described.^31^ Brewer’s thioglycollate medium (Sigma-Aldrich Chemical Co., St. Louis, MO, USA) was inoculated in BALB/c female mice 72 h prior to macrophage extraction. Peritoneal macrophages were recovered in RPMI 1640 medium (Gibco, Thermo Fisher Scientific, Waltham, MA, USA) and sedimented by centrifugation (520 × *g*, 10 min, 4 °C). The cells were seeded at 1 × 10^5^ per well in a 96-well cell culture plate and incubated in RPMI 1640 medium, supplemented with 10% FBS (100 μL of total volume) with compound in concentrations ranging from 1.95 μM to 250 μM for 24 h at 37 °C in 5% CO_2_. Additional control assays were included by treating the cell cultures with 5% DMSO (v/v). Macrophage viability was determined by addition of 10 μL of resazurin to each well and incubated for 4 h at 37 °C in 5% CO_2_. The CC_50_ values were determined by nonlinear regression analysis of log­[inhibitor] versus normalized response (%).

### 2.10 In Vitro Antileishmanial Evaluation Against Intracellular Amastigotes

Stationary-phase promastigotes of *L.*­(*V.*)­*braziliensis* Thor strain were used in macrophage infection assays at a ratio of 10:1 (parasite: macrophage) with 2 h interaction at 37 °C and 5% CO_2_. Following this period, a fresh RPMI 1640 medium containing 10% FBS was added. The total time of infection was 24 h. After that, 4 μM of the compounds or 5% DMSO (v/v) was added to a fresh RPMI 1640 medium containing 10% FBS for 24 h. Finally, macrophages in the LabTek slides were stained using the Quick Panoptic method and observed using a Panthera L Optical Microscope (Motic, Xiamen, China). The assays were performed in triplicate. To determine the percentage of infected macrophages and the number of parasites per cell, a total of 100 macrophages were analyzed per slide chamber. Then, endocytic index (EI) values were estimated, as follows: EI = (% of infected macrophages) × (mean number of parasites in each infected macrophage).

### 2.11 Statistical Analysis

Quantitative data were analyzed using GraphPad Prism version 8.0 (GraphPad Software, San Diego, CA, USA). For protease activity assays and leishmanicidal activity against intracellular amastigotes, statistical significance was determined by one-way ANOVA followed by Dunnett’s multiple comparison test to compare groups against the control. Statistically significant differences are indicated as (*) *p* < 0.05, (**) *p* < 0.01, (***) *p* < 0.001, and (****) *p* < 0.0001.

## 3 Results

### 3.1 In Silico Toxicology, Pharmacokinetic Evaluation, and Protease Interaction of Chalcones

Chalcone derivatives were analyzed to evaluate their ADMET properties, with the aim of characterizing their pharmacokinetic profiles. These analyses provided an initial assessment of the compounds, helping to prioritize those with more favorable predicted pharmacokinetic and toxicological profiles for a subsequent biological evaluation. The toxicity component was further explored through in silico toxicological analysis, including organ toxicity, toxicity end points, Tox21 pathways, molecular initiating events, and metabolism-related parameters.

#### 3.1.1 Absorption, Distribution, Metabolism, and Excretion of Chalcones

On the basis of the evaluation of chalcone molecules, a broader variation in physicochemical parameters was observed (Table S1). Overall, the MW of the chalcone derivatives ranged from 270.28 to 408.46 g/mol, remaining within the range generally considered drug-like. TMC and compounds **3a** and **3b** exhibited the lowest MW values (298.33, 270.28, and 284.31 g/mol, respectively) and relatively lower LogP values (3.00–3.61), suggesting a better balance between membrane permeability and aqueous solubility than the other derivatives.

Most of the compounds (**3b–3j**) had one hydrogen bond donor (HBD = 1), whereas **TMC** and **3a** compounds differed, with HBD values of 0 and 2, respectively. The compounds also showed a low number of hydrogen bond acceptors (HBA = 3–4), which may favor membrane permeability. TPSA values varied from 37.3 to 66.76 Å^2^, supporting adequate passive intestinal absorption. In contrast, LogP values ranged from 3.00 to 7.07, while values around 3.00 indicate optimal moderate lipophilicity, the upper range (>5.0) denotes high to extreme lipophilicity, which may influence aqueous solubility, with **3j** showing the highest hydrophobic character.

The predicted LD_50_ values were predominantly 2100 mg/kg for most of the compounds. In contrast, compounds **3a** and **3j** showed the lowest predicted LD_50_ values (1000 mg/kg), whereas **TMC**, **3b**, and **3i** exhibited the highest values (3000 mg/kg). These data indicate that the evaluated compounds differ in their predicted acute toxicities.

The predicted average structural similarity (ASS) values varied among the analyzed molecules. **TMC** and compound **3b** showed the highest scores, with values of 83.49% and 81.34%, respectively, whereas compound **3j** exhibited the lowest ASS value (53.23%). These results indicate that the evaluated derivatives share different degrees of structural similarity with compounds whose toxicity has been experimentally validated, providing additional support for the reliability of the predicted toxicity profiles of the chalcone derivatives evaluated in this study (Table S1).

#### 3.1.2 Comprehensive In Silico Toxicological Fingerprinting

The toxicological profiles of the chalcone derivatives were predicted using the ProTox-3.0 platform, which integrates conventional toxicity end points, Tox21 pathway analyses, and ADMET-related molecular targets (Figure 2). Overall, the derivatives exhibited a favorable safety profile, with low predicted probabilities for clinical toxicity, mutagenicity, carcinogenicity, cardiotoxicity, nephrotoxicity, and neurotoxicity across the evaluated end points, suggesting a reduced likelihood of systemic and long-term adverse effects. In contrast, immunotoxicity was consistently predicted for all evaluated compounds, indicating that immune-related effects may represent a common toxicological liability within this chemical series (Figure 2).

**Figure 2:**
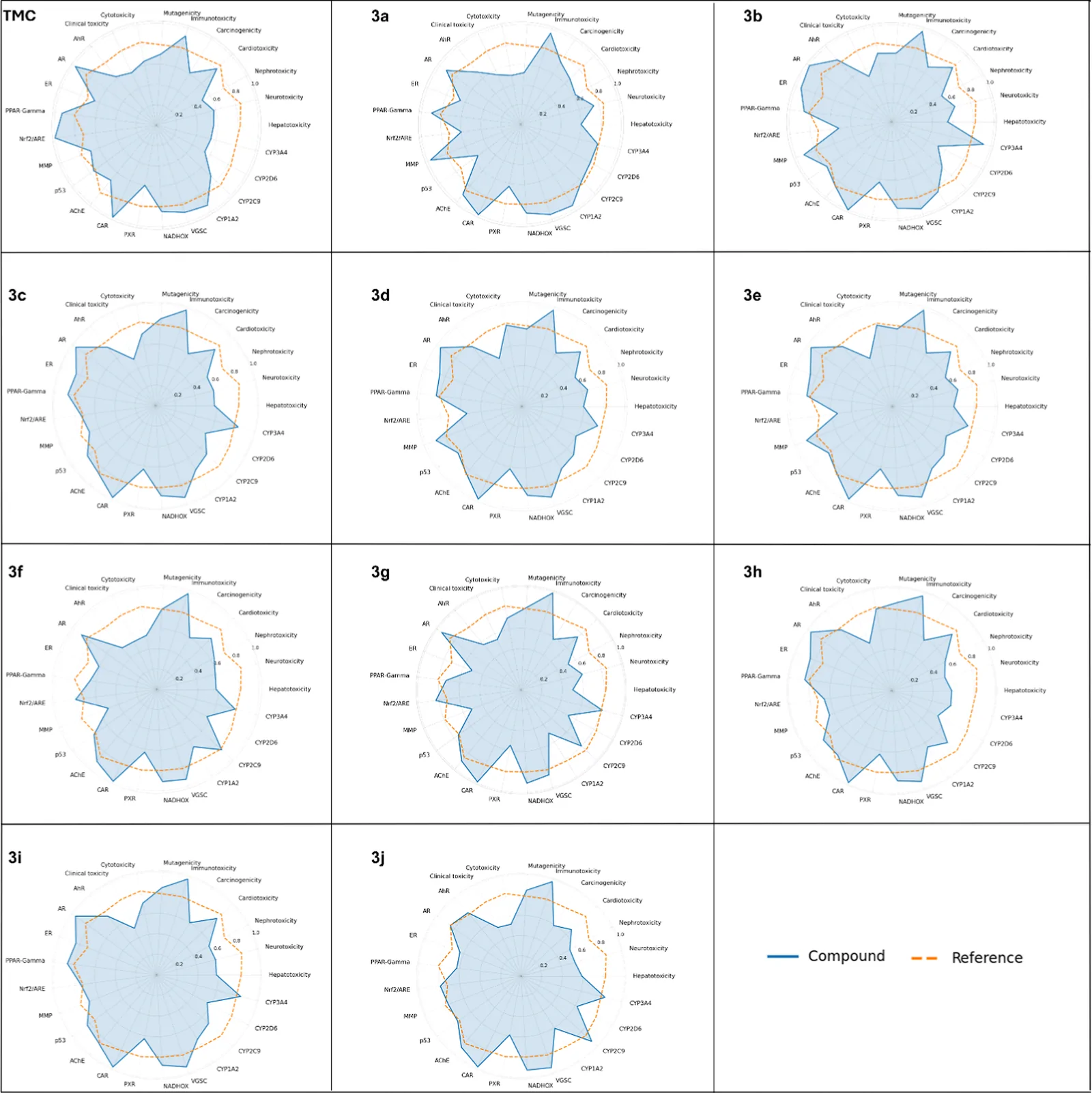
Predicted toxicological profile of chalcone derivatives. The radar plots illustrate the probability of toxicity and molecular interactions across multiple end points of chalcones: **TMC** (2′,4′,6′-trimethoxy-chalcone), **3a**, **3b**, **3c**, **3d**, **3e**, **3f**, **3g**, **3h**, **3i**, and **3j**. These include organ toxicity (hepatotoxicity, neurotoxicity, nephrotoxicity, and cardiotoxicity), toxicological outcomes (carcinogenicity, immunotoxicity, mutagenicity, cytotoxicity, and clinical toxicity), and signaling pathways (Aryl hydrocarbon Receptor—**AhR**; Androgen Receptor—**AR**; Estrogen Receptor Alpha—**ER**; Peroxisome Proliferator Activated Receptor Gamma -**PPAR-γ**); Cellular stress responses (Nuclear factor (erythroid-derived 2)-like 2/antioxidant responsive element—**nrf2/ARE**; Mitochondrial Membrane Potential—**MMP**; Phosphoprotein (Tumor Suppressor)—**p53**), molecular initiating events (Achetylcholinesterase—**AchE**; Constitutive androstane receptor—**CAR**; NADH-quinone oxidoreductase—**NADHOX**; Voltage-gated sodium channel—**VGSC**); and metabolic profiles (Cytochrome P450 isoforms: **CYP1A2**, **CYP2C9**, **CYP2D6**, **CYP3A4, CYP2C19**, and **CYP2E1**) were also evaluated. Predictions were generated using the ProTox-3.0 server. The solid line represents the predicted profile of the analyzed compound, while the dashed line corresponds to the average profile of active compounds from the ProTox-3.0 data set used to build the prediction models. Higher values indicate a greater probability of target interaction or toxicity.^20^

Comparative analysis of the Tox21 and ADMET radar plots revealed distinct differences among the chalcone derivatives with respect to their predicted interactions with toxicity-associated molecular targets. Among the evaluated compounds, **3e**, **3d**, and **3a** displayed the most favorable toxicological profiles, characterized by homogeneous distributions and consistently lower predicted interactions with key Tox21 targets, including AR, PPAR-γ, CAR, CYP1A2, and VGSC, relative to the **TMC** compound. These derivatives also maintained low predicted toxicity across the remaining ADMET end points, supporting an overall improvement in the silico safety profile.

Compounds **3c**, **3j**, and **3i** also demonstrated favorable toxicological characteristics, presenting balanced profiles with only modest increases in isolated end points, mainly involving MMP or selected cytochrome P450 isoforms, while remaining below the corresponding values observed for **TMC** in most evaluated parameters. Likewise, **3h** and **3b** exhibited toxicological profiles comparable to those of the reference compound but with lower predicted interactions across several nuclear receptor signaling pathways. Nevertheless, these derivatives showed relatively higher scores for specific end points, including VGSC, CYP3A4, and Nrf2/ARE, resulting in a slightly less favorable overall profile.

In contrast, **3g** and **3f** presented the least homogeneous toxicological profiles among the derivatives. Although both compounds exhibited reduced predicted interactions with several nuclear receptor signaling pathways compared with **TMC**, they showed comparatively higher scores for stress response pathways, particularly Nrf2/ARE, p53, and MMP, suggesting a greater predicted propensity to modulate cellular stress-response mechanisms. Notably, the compound **TMC** exhibited the highest predicted interactions with several toxicity-related molecular targets, including AR, PPAR-γ, Nrf2/ARE, CAR, NADHox, VGSC, and CYP1A2.

### 3.2 Protease Activity Inhibition and Antileishmanial Effects of Chalcone Derivatives

This study also addresses the direct effects of the new chalcones on the parasite proteases and parasite viability. To investigate these effects, the ability of the synthesized chalcones to inhibit the activity of cysteine and serine proteases in promastigote extracts was first evaluated. This biochemical screening was then integrated with phenotypic assays to establish a correlation between protease inhibition and the overall antileishmanial efficacy of the most promising derivatives.

#### 3.2.1 Inhibition of Protease Activities from Promastigotes

The inhibition of protease activities was evaluated in *L.*­(*V.*)­*braziliensis* extracts incubated with the test compounds under buffer conditions optimized to favor the enzymatic activity of each protease class. The potential inhibitory effects were corroborated by comparing enzymatic activity in the absence and presence of specific protease inhibitors, as illustrated by the inhibition profiles observed with aprotinin in Tris–HCl buffer (pH 7.5) and E-64 in sodium acetate buffer (pH 5.5) (Figures 3, S47, and S48).

**Figure 3:**
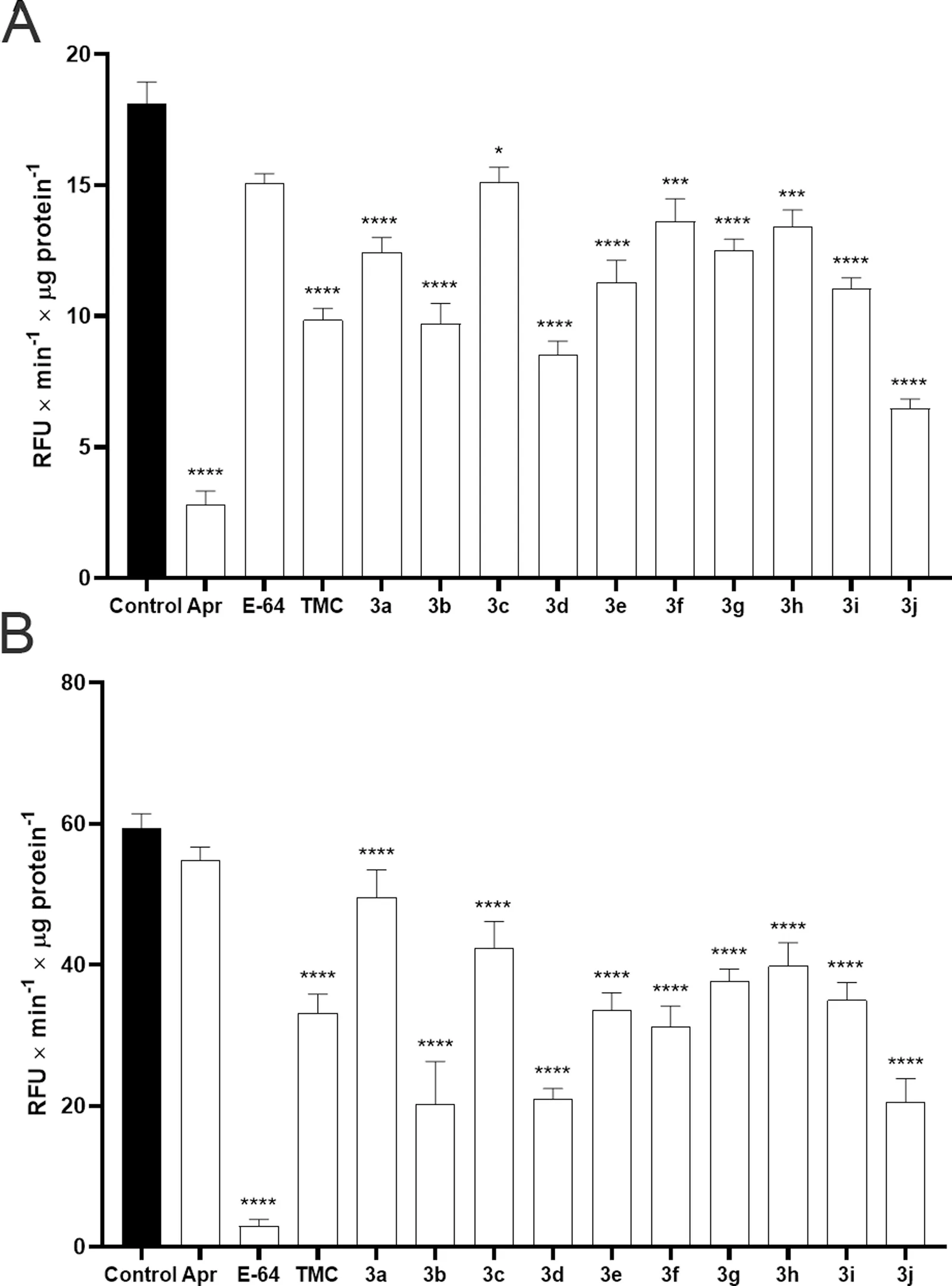
Protease activity of *Leishmania*­(*V.*)­*braziliensis* extracts in the presence and absence of compounds. The activity was evaluated in 10 mM Tris–HCl pH 7.5 (A) and 10 mM sodium acetate buffer pH 5.5 containing 1 mM dithiothreitol (B). The *x*-axis shows the experimental conditions analyzed: 0.3 μM aprotinin (Apr), 10 μM E-64, 0.4 mM of compounds (**TMC**, **3a**, **3b**, **3c**, **3d**, **3e**, **3f**, **3g**, **3h**, **3i**, and **3j**). The results are shown on the *y*-axis as relative fluorescence units per minute per microgram of protein (RFU × min^–1^ × μg of protein^–1^) and are expressed as the mean ± standard error of three independent experiments performed in triplicate. Differences between experimental groups and the solvent control (5.0% DMSO used to dilute the compounds), which represented 100% of enzyme activity (control). Data were analyzed by one-way ANOVA followed by Dunnett’s multiple comparisons test, with each treatment group compared with the control group. Statistically significant differences are indicated as (*) *p* < 0.05, (***) *p* < 0.001, and (****) *p* < 0.0001.

To establish reference activity levels, control velocity values were determined for each buffer condition. In Tris–HCl buffer, the enzymatic activity was 18.8 ± 2.4 RFU × min^–1^ × μg protein^–1^, while the corresponding value in the presence of DMSO was 18.1 ± 2.9 RFU × min^–1^ × μg protein^–1^. In sodium acetate buffer, the control activity was higher, reaching 56.3 ± 1.6 RFU × min^–1^ × μg protein^–1^, with the DMSO control yielding 59.3 ± 2.1 RFU × min^–1^ × μg protein^–1^.

The residual activities observed in the presence of the test compounds revealed distinct inhibition patterns. In the Tris–HCl buffer, aprotinin showed the most pronounced inhibitory effect (15.46% residual activity), followed by **3j** (36%), **3d** and **TMC** (47%), and **3b** (53%). In acetate buffer, the most effective inhibitors were E-64 (5%), followed by **3b** and **3j** (34%) and **3d** (35%) (Figure 3).

To further characterize the inhibitory potential of these novel chalcones and enable a comparative assessment of their efficacy, IC_50_ values were determined under both protease activity conditions, providing a quantitative measure of these compounds’ potency as cysteine protease and serine protease inhibitors (Table 1) All compounds exhibited dose-dependent inhibition with IC_50_ values ranging from 0.056 ± 0.018 mM to 0.178 ± 0.018 mM in sodium acetate buffer (pH 5.5) and from 0.10 ± 0.001 mM to 0.143 ± 0.027 mM in Tris–HCl buffer (pH 7.5). In sodium acetate buffer, compound **3i** was the most potent inhibitor (IC_50_ = 0.056 ± 0.018 mM), followed by **3d** (0.068 ± 0.009 mM). Conversely, in Tris–HCl buffer, compounds **3j** (0.010 ± 0.001 mM) and **3i** (0.010 ± 0.0004 mM) showed the highest inhibitory activity, reaching a remarkably low IC_50_, followed by **3e** (0.020 ± 0.010 mM). Notably, most compounds, including the reference **3b** demonstrated enhanced potency (lower IC_50_ values) at physiological pH 7.5 compared to acidic conditions pH 5.5, suggesting that the catalytic efficiency or the binding affinity of these chalcones is sensitive to medium alkalinity.

**Table 1:** IC_50_ Values of Chalcones for Protease Activity Evaluated under Cysteine Protease and Serine Protease Conditions in Protein Extracts from Stationary-Phase Promastigotes *Leishmania*­(*V.*)­*braziliensis* ^a^

	IC_50_ (mM)	
compounds	sodium acetate buffer pH 5.5	Tris–HCl buffer pH 7.5
**TMC**	0.096 ± 0.019	0.122 ± 0.0060
**3a**	0.164 ± 0.012	0.073 ± 0.0330
**3b**	0.088 ± 0.010	0.057 ± 0.0140
**3c**	0.082 ± 0.004	0.107 ± 0.0040
**3d**	0.068 ± 0.009	0.071 ± 0.0260
**3e**	0.076 ± 0.027	0.020 ± 0.0100
**3f**	0.082 ± 0.010	0.143 ± 0.0270
**3g**	0.132 ± 0.008	0.039 ± 0.0160
**3h**	0.178 ± 0.018	0.111 ± 0.0150
**3i**	0.056 ± 0.018	0.010 ± 0.0004
**3j**	0.076 ± 0.011	0.010 ± 0.0010

a Half-maximal inhibitory concentration (IC_50_); 2′,4′,6′-trimethoxy-chalcone (**TMC**).

#### 3.2.2 Antileishmanial Activity and Cytotoxicity in Murine Macrophages

The antileishmanial activity of 11 compounds was first assessed against *L.*­(*V.*)­*braziliensis* promastigotes (Table 2 and Figure S49), and their cytotoxicity in murine macrophages was evaluated to identify the most selective candidates (Table 2). Cell viability was determined on the basis of the reduction of resazurin to resorufin by metabolically active cells.

**Table 2:** In Vitro Effects of Chalcones on Promastigote Forms of *Leishmania*­(*V.*)­*braziliensis* and on Murine Peritoneal Macrophages^a^

compounds	promastigote IC_50_ (μM)	macrophage CC_50_ (μM)	SI
**TMC**	7.31 ± 1.53	160.01 ± 6.92	21.88
**3a**	14.16 ± 0.79	45.62 ± 8.61	3.22
**3b**	7.66 ± 0.84	136.3 ± 18.62	17.79
**3c**	8.02 ± 1.34	40.03 ± 4.22	4.99
**3d**	9.76 ± 2.43	163.45 ± 3.04	16.74
**3e**	29.73 ± 2.24	35.80 ± 4.98	1.20
**3f**	3.352 ± 0.05	25.38 ± 2.71	7.57
**3g**	5.97 ± 1.11	25.61 ± 1.43	4.28
**3h**	8.08 ± 0.29	177.06 ± 10.66	21.91
**3i**	2.08 ± 0.24	213.23 ± 7.91	102.17
**3j**	13.25 ± 1.49	37.87 ± 7.75	2.85
amphotericin B	0.38 ± 0.09	67.88 ± 0.94	178.63

a Selectivity index (SI); half-maximal inhibitory concentration (IC_50_); half-maximal cytotoxic concentration (CC_50_); 2′,4′,6′-trimethoxy-chalcone (**TMC**).

The IC_50_ values against *L.*­(*V.*)­*braziliensis* promastigotes ranged from 2.08 ± 0.24 μM (**3i**) to 29.73 ± 2.24 μM (**3e**), while the CC_50_ values in murine macrophages varied from 25.38 ± 2.71 μM (**3f**) to 213.23 ± 7.91 μM (**3i**). These results showed that most compounds exhibited low CC_50_ values in macrophages, indicating potential cytotoxicity to host cells. Based on these results, only five compounds exhibited selectivity index (SI) values greater than 10: **3d** (16.74), **3b** (17.79), **TMC** (21.88), **3h** (21.91), and **3i** (102.17), while the amphotericin B, used as a positive control, showed an SI of 178.63 (Table 2).

The effect of the compounds on murine macrophage infection by *L.*­(*V.*)­*braziliensis* was also evaluated. At this stage, only compounds with SI greater than 10 were selected for further *in vitro* efficacy testing as this threshold is commonly used to ensure a minimum safety margin between antileishmanial activity and host cell toxicity. All tested compounds reduced the EI compared to the control group (Figure 4). The greatest reduction was observed with compound **3i** (64%), followed by **3d** (52%), **3b** (49%), **3h** (40%), and **TMC** (34%), in comparison to the control group (Figure 4). Representative images of infected macrophages that were either untreated (control) or treated with 4 μM of compounds **TMC**, **3b**, **3d**, **3h**, and **3i** show the effects of these compounds on intracellular amastigotes (Figure S50).

**Figure 4:**
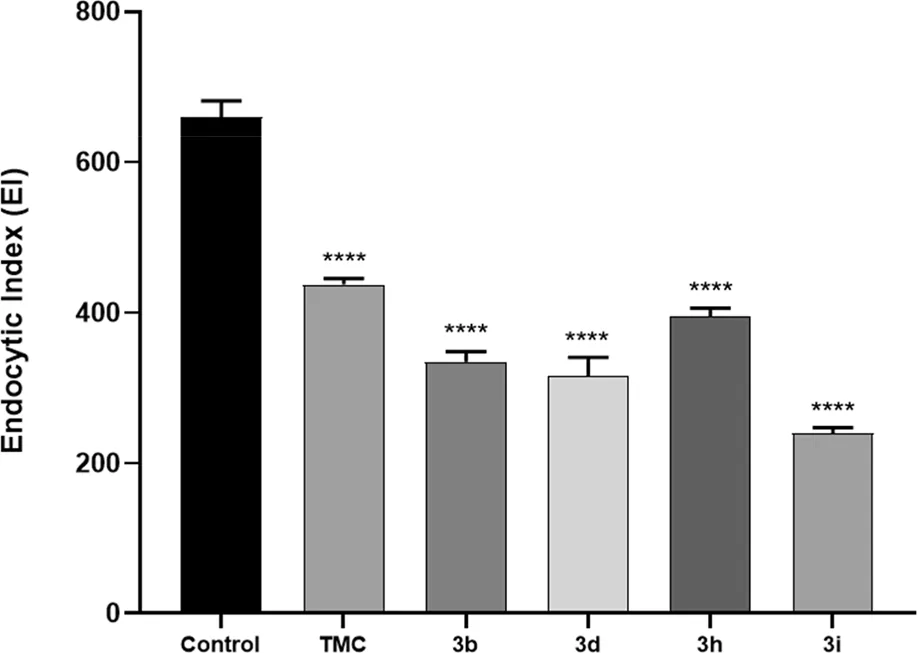
Effect of the compounds on the infection rate of *Leishmania*­(*V.*)­*braziliensis* amastigotes in murine macrophages in vitro. BALB/c macrophages were infected with parasites at a ratio of 10:1 (parasite: macrophage) for 24 h. Then, cells were treated for 24 h with 4.0 μM of the compounds 2′,4′,6′-trimethoxy-chalcone (**TMC**), **3b**, **3d**, **3h**, and **3i**, while the control group (control) received an equivalent concentration of dimethyl sulfoxide, as shown on the *x*-axis. The endocytic index (EI), shown on the *y*-axis, is expressed as mean ± standard deviation of three independent experiments performed in triplicate. Data were analyzed by one-way ANOVA followed by Dunnett’s multiple comparisons test, with each treatment group compared with the control group. Statistically significant differences are indicated as ****p < 0.0001.

## 4 Discussion

Leishmaniasis, particularly caused by *L.*­(*V.*)­*braziliensis*, remains a significant public health concern in endemic regions, with current treatment options being limited, frequently toxic, and increasingly ineffective due to the emergence of antimony-resistant strains.^32,33^ Amid these constraints, there is growing recognition that therapeutic failure may be partly associated with the limited molecular scope of current drugs. Therefore, compounds capable of interfering with multiple parasite targets, particularly enzymes essential for survival and virulence, represent a rational strategy to enhance the therapeutic efficacy and mitigate the development of resistance.

In this context, the findings of this work further support the relevance of synthetic chalcones as molecules with the potential to simultaneously inhibit multiple protease activities in *L.*­(*V.*)­*braziliensis*, in agreement with previous in silico predictions.^19^ To explore this potential, specific chalcone hits were identified as templates for the structural refinement. Building on the potent leishmanicidal effects previously observed for **3a** and **3b** against promastigote forms of *L*. (*L.*) *amazonensis* and *L.*­(*V.*)­*braziliensis*,^34,35^ the structural optimization of the chalcone scaffold was investigated. This involved increasing the degree of methylation and introducing diverse aryl substituents on the A ring.^21^ To enable this structural diversification, it was necessary to establish a synthetic strategy that would facilitate the incorporation of different aryl substituents while overcoming the limitations of the conventional chalcone synthesis. Accordingly, a new synthetic approach was developed to obtain these chalcones from the flavones.

Although the aldol condensation is the broadly employed approach to prepare chalcones, further modifications on the chalcone structure, e.g., palladium/nickel/ruthenium catalyzed cross-coupling reactions, are difficult, resulting in no or low yields and isomerization of the double bond. As previously published work,^21^ where arylflavones were synthesized, here it was converted to arylchalcones through reduction of flavone C ring double bond and opening of the flavanone C ring under strong basic condition (*t*-BuOK-DMSO). This newly developed methodology allows us to synthesize arylchalcones from arylflavanones with good to excellent yields. Moreover, this methodology offers a practical alternative for the synthesis of arylchalcones by converting previously synthesized aryl flavones into the desired products. Using a common synthetic intermediate, the approach provides access to different flavonoid scaffolds in good to excellent yields, while expanding the structural diversity of compounds available for biological evaluation.

Beyond its synthetic advantages, this approach was also designed to preserve the α,β-unsaturated carbonyl moiety, a key structural feature associated with the biological activity of chalcones.^36,37^ The preservation of this electrophilic motif may explain, at least in part, the biological and biochemical effects observed for the synthesized derivatives, including their activity against both promastigotes and amastigotes and their inhibitory effects on cysteine and serine proteases, two enzyme classes essential for parasite survival and virulence.^9^ This observation is consistent with the chemical design strategy adopted in this study. Although these derivatives were designed to target *Leishmania* proteases, their enone group may also allow interactions with other molecular targets. As an electrophilic structural feature, this group can react with nucleophilic amino acid residues, particularly cysteine, lysine, and histidine, which are widely distributed in different proteins.^14^

As is well established, the electrophilic moiety of chalcones can undergo Michael addition reactions, particularly with thiol groups of catalytic cysteine residues in proteases, as proposed for active-site amino acids of *L.*­(*V.*)­*braziliensis* Cysteine Protease B and Oligopeptidase B.^19^ Through binding interactions with the target enzyme, such compounds may inhibit protease activity, thereby impairing essential processes in parasite survival and virulence. Therefore, the preserved presence of this electrophilic motif in the novel chalcone derivatives described here suggests a plausible mechanism for the observed inhibition of both cysteine protease and serine protease activities in *L.*­(*V.*)­*braziliensis*, which may have contributed to their leishmanicidal effect. However, these results were obtained using a crude parasite extract, in which multiple proteolytic activities may coexist and be strongly influenced by the reaction pH. Thus, the distinct activity profiles observed under acidic and neutral conditions reflect the overall proteolytic activities of the extract. Further assays are therefore required to determine the specific inhibition of individual protease classes by these compounds. In addition, this inhibition profile indicates a polypharmacological profile for these compounds, which may be advantageous in overcoming compensatory mechanisms associated with parasite survival and drug resistance.^38^

Within this broader pharmacological framework, the simultaneous inhibition of more than one protease class observed here aligns with the ongoing paradigm shift in drug discovery from single target toward multitarget strategies.^39^ This concept was evidenced here for the new chalcones **3d**, **3h**, and **3i**, which demonstrated varying IC_50_ values for the inhibition of both cysteine and serine protease activities, key enzymes in the physiology of *L.*­(*V.*)­*braziliensis*.

These findings are underscored by the inhibitory profiles of these chalcones, which indicated to be potent inhibitors across distinct biochemical environments. This inhibition was maintained in pH 5.5 and pH 7.5 conditions. Both pH activity is particularly relevant because it suggests that the chalcone scaffold remains active under conditions representative of both the acidic phagolysosome, favoring inhibition of cysteine proteases, and near-physiological environments, where serine proteases are also active.^40,41^ Therefore, these novel derivatives follow a consistent trend, acting as broad-spectrum inhibitors of the parasite’s enzymatic machinery and suggesting their potential as multitarget candidates. Considering that protease genes comprise 2.18% of the *L.*­(*V.*)­*braziliensis* genome,^42^ the ability of these compounds to inhibit multiple enzyme classes simultaneously offers a significant advantage. By suppressing enzymes essential for immune evasion and host cell invasion,^9^ these chalcones present a multitarget mechanism that may be less susceptible to the development of drug resistance.

Evaluation of the chalcone derivatives according to Lipinski’s rule of five showed that most compounds fulfilled the recommended criteria for molecular weight and hydrogen bond donor and acceptor counts. The main exception was lipophilicity, as compounds **3c–3j** presented predicted LogP values above the recommended threshold, with the highest values observed for compounds **3g** and **3j**. Despite this, no increase in predicted hepatotoxicity was observed in the ProTox-3.0 analysis. Even compound 3j, which showed predicted interactions with liver metabolism-related targets, was not classified as hepatotoxic. These findings indicate that in silico parameters should not be interpreted in isolation when assessing the drug-likeness of a compound, but rather as part of an integrated evaluation that includes physicochemical, pharmacokinetic, toxicological, and biological data. In this regard, the agreement between the computational predictions and the experimental antileishmanial activity observed for compounds **3d**, **3h**, and **3i** further supports the value of the integrated in silico/in vitro strategy adopted in this study for identifying promising lead compounds. While the in silico analyses provided important information regarding the pharmacokinetic and toxicological profiles of the chalcone derivatives, their therapeutic potential ultimately depends on the balance between antiparasitic activity and host-cell safety.

In this context, amphotericin B, a well-established antileishmanial drug, was used as the reference compound in the *in vitro* assays because of its well-documented efficacy. However, its clinical use is often limited by systemic toxicity.^43,44^ By comparison, most of the new chalcone derivatives retained potential antileishmanial activity while preserving macrophage viability, resulting in favorable selectivity indices and supporting their potential as promising lead compounds. Additionally, the residual cytotoxicity observed for all chalcones evaluated in this study deserves further consideration. One possible explanation for this effect is the electrophilic nature of the double bond conjugated with the carbonyl group, which may also interact with amino acid residues in host-cell proteins. This should be considered in future optimization studies aimed at improving the selectivity of the most active derivatives while maintaining their antileishmanial activity.

From a translational perspective, however, the therapeutic relevance of multitarget activity critically depends on the balance between antiparasitic efficacy and host safety. In the screening of antiprotozoal drugs, particularly for cutaneous leishmaniasis, an SI value ≥10 is the widely accepted minimum threshold for an acceptable safety margin and specificity.^45,46^ This criterion is central to standard protocols for prioritizing antileishmanial compounds for further development. Therefore, the selected compounds are considered strong candidates for further preclinical investigation. Given their potent leishmanicidal activity and high selectivity, these molecules meet the requirements for advancement into *in vivo* proof-of-concept validation.

## 5 Conclusion

Taken together, the results of this study highlight the potential of synthetic chalcones as promising antileishmanial compounds. Their polypharmacological profile is suggested by their ability to inhibit different protease classes involved in the survival and virulence of *L.*­(*V.*)­*braziliensis*. At this stage, the results obtained do not allow definitive conclusions regarding the binding modes of the compounds to their intended molecular targets or whether the observed inhibition is reversible or involves covalent interactions.

The arylchalcones were successfully synthesized by using the proposed synthetic strategy. Furthermore, the flavanone C-ring-opening methodology developed in this work proved to be an efficient strategy to prepare chalcones from flavanones. The rational chemical design, centered on preserving the double bond conjugated to the carbonyl group, supports a plausible mechanism of enzymatic inhibition.

The results gathered here highlight new derivatives **3d**, **3h**, and **3i**, which combine protease-inhibitory and antiparasitic activities with high potency and favorable selectivity indices. These findings are supported by in silico assessments, which predict a favorable toxicological profile and acceptable drug-like properties and a low risk of organ toxicity for these specific analogues. These compounds may represent safe and promising candidates, warranting further preclinical investigations.

## Supplementary Material



## Data Availability

All data generated or analyzed during this study are included in this article.

## Notes

Physicochemical properties, drug-likeness parameters, and predicted toxicity of the synthesized chalcone; synthesis of chalcone 2′,4′,6′-trimethoxy-chalcone (TMC); high-resolution ESI-Qq-TOF of newly synthesized compounds; HPLC-UV and HPLC-DAD of biological evaluated compounds; nuclear magnetic resonance spectra of synthesized compounds; and biochemistry and biological assays (PDF)
