# Supplementary material for: Novel Synthetic Chalcones Display Potent Anti-Leishmania (Viannia) braziliensis Profile and Inhibit Proteolytic Activities
Source: ACS Omega. 2026 Sep 29;11(40):61265–77. doi: 10.1021/acsomega.6c06140 (PMC13670310; doi:10.1021/acsomega.6c06140)
Supplement: Supplementary file 1 [file ao6c06140_si_001.pdf]

Supplementary File

Novel Synthetic Chalcones Display Potent Anti-*Leishmania* (*Viannia*) *braziliensis* Profile and Inhibit Proteolytic Activities

Edgar Schaeffer[1*], Geovane Dias-Lopes[2,3*], Patrícia Queiroz Monteiro[3], Julia Maria Borges[3], Luzia Monteiro de Castro Côrtes[3], Alcides José Monteiro da Silva[1], Franklin Souza da Silva[4,5*], Carlos Roberto Alves[3**]

[1]Universidade Federal do Rio de Janeiro, UFRJ, Instituto de Pesquisas de Produtos Naturais Walter Mors, Laboratório de Catálise Orgânica, Av. Carlos Chagas Filho 373 - Bloco H, CEP 21941-599, Cidade Universitária, Rio de Janeiro-RJ, Brasil.

[2]Universidade do Estado do Rio de Janeiro, UERJ, Departamento de Ciências Biomédicas e Saúde, Instituto de Biologia Roberto Alcântara Gomes, Rua Arízio Gomes da Costa 186, CEP – 28905-320, Jardim Flamboyant, Cabo Frio, RJ, Brasil.

[3]Fundação Oswaldo Cruz, FIOCRUZ, Instituto Oswaldo Cruz, Laboratório de Biologia Molecular e Doenças Endêmicas, Avenida Brasil 4365, CEP 21040-900, Manguinhos, Rio de Janeiro, RJ, Brasil.

[4]Universidade Iguaçu, UNIG, Laboratório de Pesquisas Pré-clínicas, Avenida Abílio Augusto Távora 2134, CEP 26260-045, Dom Rodrigo, Nova Iguaçu, RJ, Brasil.

[5]Fundação Oswaldo Cruz, Centro de Desenvolvimento Tecnológico em Saúde, Avenida Brasil, 4036 - Manguinhos, Rio de Janeiro - RJ, CEP 21040-361, Manguinhos, Rio de Janeiro, RJ, Brasil.

[*]These authors contributed equally to this work

[**]Corresponding author: calves@ioc.fiocruz.br

Supplementary File

TABLE OF CONTENTS

1. **Physicochemical Properties of Chalcones** 4

Table S1. Physicochemical properties, drug-likeness parameters, and predicted toxicity of the synthesized chalcone 4

2. **Synthesis of Chalcone 2′,4′,6′-trimethoxy-chalcone (TMC)** 6

Figure S1. Scheme of synthesis of chalcone TMC 6

3. **High-resolution ESI-Qq-TOF of Newly Synthesized Compounds** 7

Figure S2. High-resolution ESI-Qq-TOF mass spectrum of **2c** 7

Figure S3. High-resolution ESI-Qq-TOF mass spectrum of **2d** 8

Figure S4. High-resolution ESI-Qq-TOF mass spectrum of **2e** 9

Figure S5. High-resolution ESI-Qq-TOF mass spectrum of **2f** 10

Figure S6. High-resolution ESI-Qq-TOF mass spectrum of **2g** 11

Figure S7. High-resolution ESI-Qq-TOF mass spectrum of **2h** 12

Figure S8. High-resolution ESI-Qq-TOF mass spectrum of **2i** 13

Figure S9. High-resolution ESI-Qq-TOF mass spectrum of **2j** 14

Figure S10. High-resolution ESI-Qq-TOF mass spectrum of **3c** 15

Figure S11. High-resolution ESI-Qq-TOF mass spectrum of **3d** 16

Figure S12. High-resolution ESI-Qq-TOF mass spectrum of **3e** 17

Figure S13. High-resolution ESI-Qq-TOF mass spectrum of **3f** 18

Figure S14. High-resolution ESI-Qq-TOF mass spectrum of **3g** 19

Figure S15. High-resolution ESI-Qq-TOF mass spectrum of **3h** 20

Figure S16. High-resolution ESI-Qq-TOF mass spectrum of **3i** 21

Figure S17. High-resolution ESI-Qq-TOF mass spectrum of **3j** 22

4. **HPLC-UV and HPLC-DAD of Biological Evaluated Compounds** 23

Figure S18. HPLC-UV chromatogram (254 nm) of **3c** 23

Figure S19. HPLC-UV chromatogram (254 nm) of **3d** 24

Figure S20. HPLC-UV chromatogram (254 nm) of **3e** 25

Figure S21. HPLC-UV chromatogram (254 nm) of **3f** 26

Figure S22. HPLC-UV chromatogram (254 nm) of **3g** 27

Figure S23. HPLC-UV chromatogram (254 nm) of **3h** 28

Figure S24. HPLC-DAD chromatogram (254 nm) of **3i** 29

Figure S25. HPLC-DAD chromatogram (254 nm) of **3j** 30

5. **Nuclear Magnetic Resonance Spectra of Synthesized Compounds** 31

Figure S26. ^{1}H and ^{13}C APT NMR spectra of compound **2a** 31

Figure S27. ^{1}H and ^{13}C APT NMR spectra of compound **2b** 32

Figure S28. ^{1}H and ^{13}C APT NMR spectra of compound **2c** 33

Figure S29. ^{1}H and ^{13}C APT NMR spectra of compound **2d** 34

Figure S30. ^{1}H and ^{13}C APT NMR spectra of compound **2e**......................................35
Figure S31. ^{1}H and ^{13}C APT NMR spectra of compound **2f** 36
Figure S32. ^{1}H and ^{13}C APT NMR spectra of compound **2g** 37
Figure S33. ^{1}H and ^{13}C APT NMR spectra of compound **2h** 38
Figure S34. ^{1}H and ^{13}C APT NMR spectra of compound **2i**.......................................39
Figure S35. ^{1}H and ^{13}C APT NMR spectra of compound **2j**.......................................40
Figure S36. ^{1}H and ^{13}C APT NMR spectra of compound **3a/Pinostrobin chalcone** .41
Figure S37. ^{1}H and ^{13}C APT NMR spectra of compound **3b/Flavokawain B** 42
Figure S38. ^{1}H and ^{13}C APT NMR spectra of compound **3c**......................................43
Figure S39. ^{1}H and ^{13}C APT NMR spectra of compound **3d** 44
Figure S40. ^{1}H and ^{13}C APT NMR spectra of compound **3e**......................................45
Figure S41. ^{1}H and ^{13}C APT NMR spectra of compound **3f** 46
Figure S42. ^{1}H and ^{13}C APT NMR spectra of compound **3g** 47
Figure S43. ^{1}H and ^{13}C APT NMR spectra of compound **3h** 48
Figure S44. ^{1}H and ^{13}C APT NMR spectra of compound **3i**.......................................49
Figure S45. ^{1}H and ^{13}C APT NMR spectra of compound **3j**.......................................50
Figure S46. ^{1}H and ^{13}C APT NMR spectra of compound **2′,4′,6′-trimethoxy-chalcone (TMC)**...51
6. Biochemistry and Biological Assays...52
Figure S47. Proteolytic inhibition assays of chalcone derivatives in Tris-HCl buffer ..52
Figure S48. Proteolytic inhibition assays of chalcone derivatives in acetate buffer....53
Figure S49. Promastigote viability profiles...54
Figure S50. Representative light microscopy images of *Leishmania* (*V.*) *braziliensis*-infected murine macrophages treated with TMC and arylchalcone derivatives...........55

Supplementary File

1. Physicochemical Properties of Chalcones

Table S1. Physicochemical properties, drug-likeness parameters, and predicted toxicity of the synthesized chalcone

Compounds ID		Structure	MW	HBA	HBD	nAtoms	nBonds	nRotB	MR	TPSA (Å²)	LogP	Predicted LD50 (mg/kg)	ASS (%)
**Monteiro et al., (2025)**	**This publication**												
**-**	**TMC**	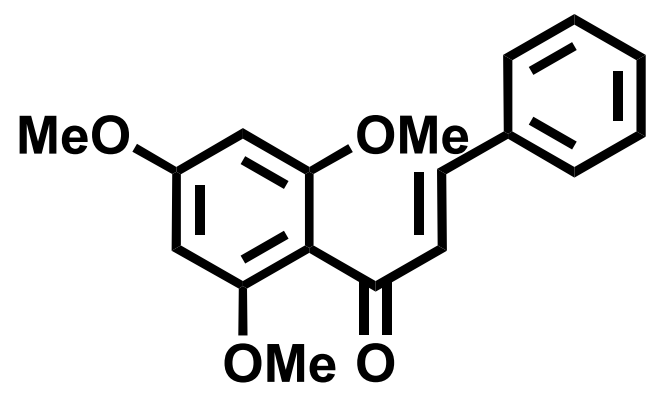	298.33	4	0	22	23	6	85.72	44.76	3.61	3000	83.49
**-**	**3a**	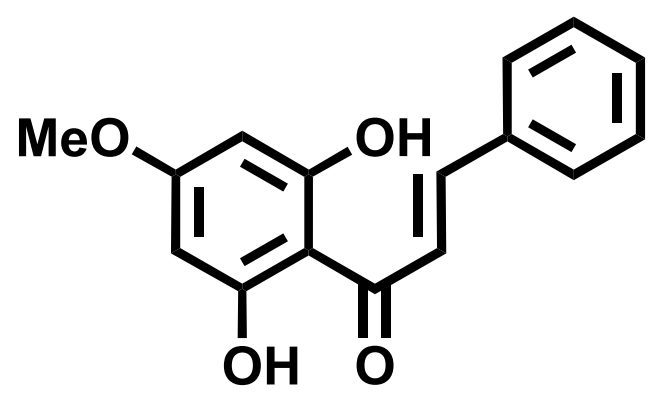	270.28	4	2	20	21	4	76.79	66.76	3.00	1000	81.34
**Flavokawain B***	**3b**	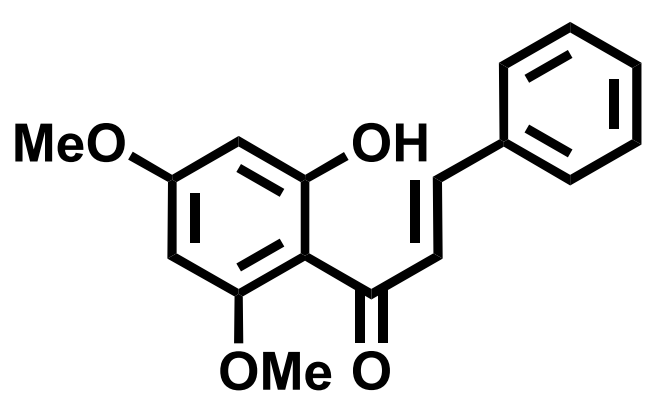	284.31	4	1	21	22	5	81.26	55.76	3.31	3000	83.49
**C01***	**3c**	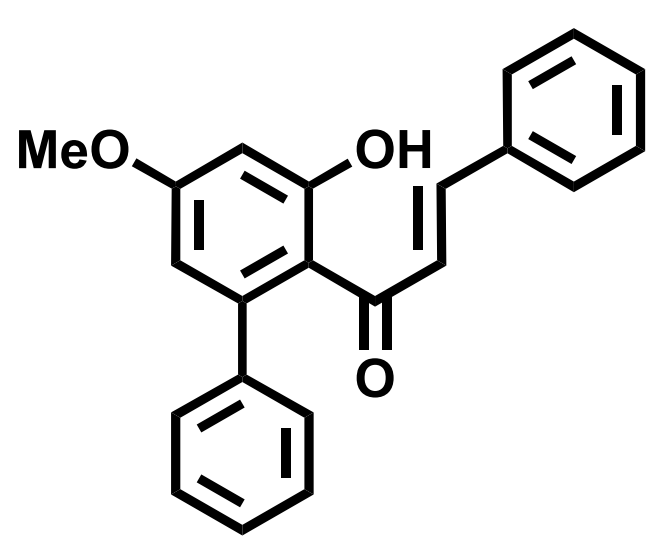	330.38	3	1	25	27	5	100.2	46.53	4.96	2100	65.66
**C02***	**3d**	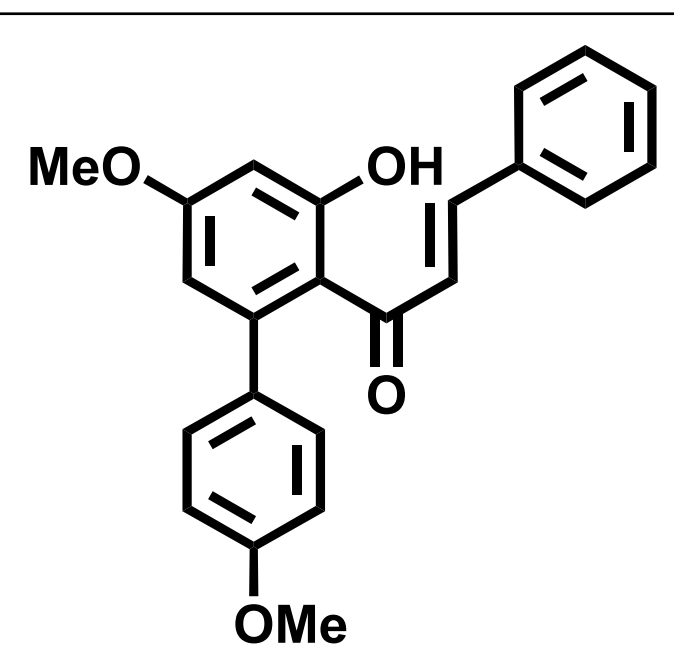	360.4	4	1	27	29	6	106.69	55.76	4.97	2100	65.03
**C03***	**3e**	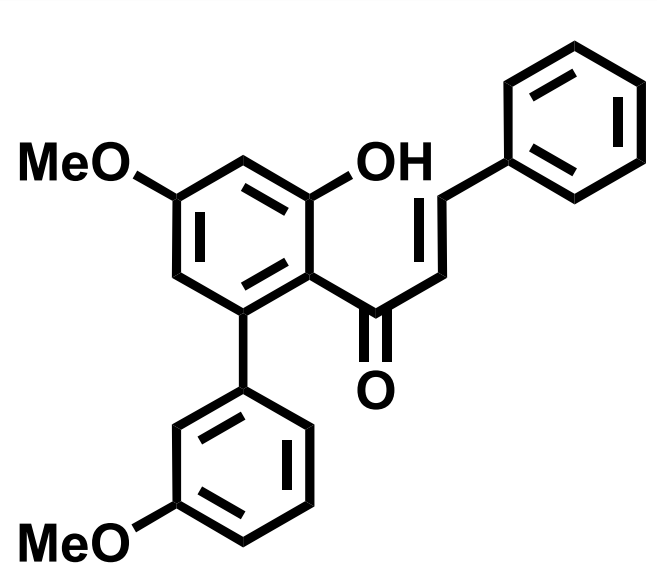	360.4	4	1	27	29	6	106.69	55.76	4.97	2100	65.03
**C08***	**3f**	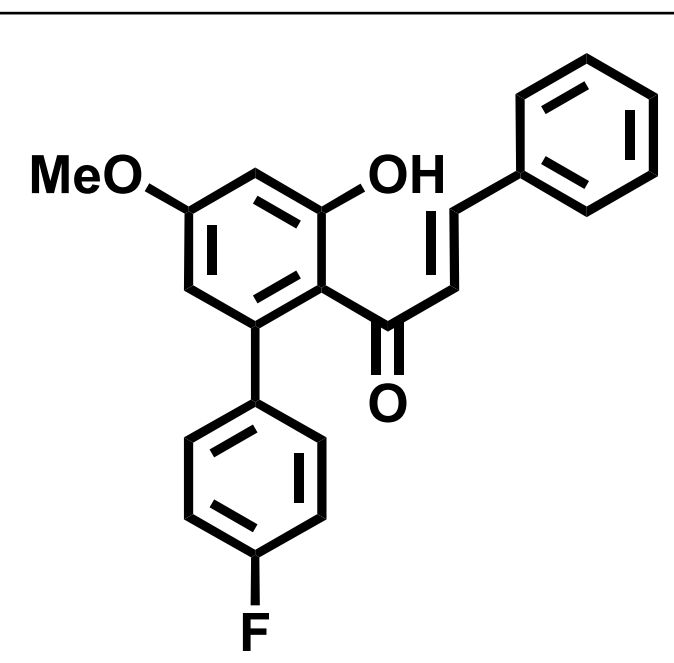	348.37	3	1	26	28	5	100.16	46.53	5.10	2100	60.33
**C09***	**3g**	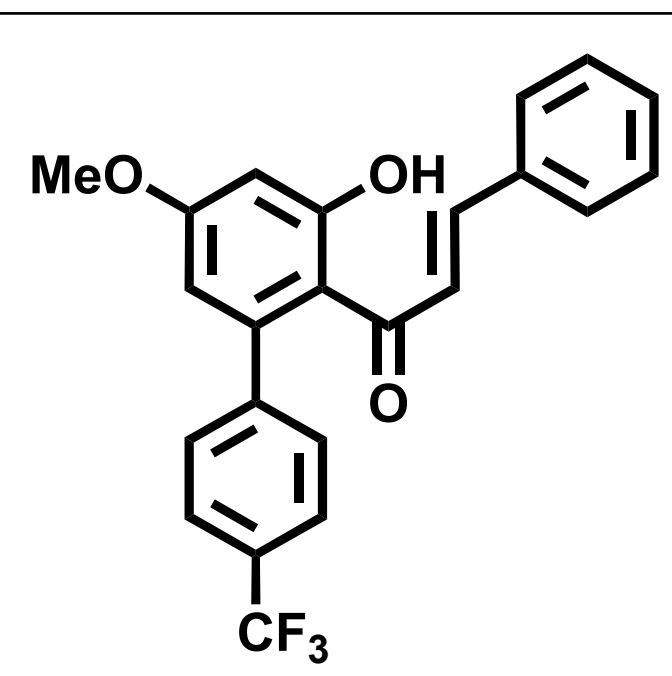	398.37	3	1	29	31	6	105.2	46.53	5.98	2100	59.3

**-**	**3h**	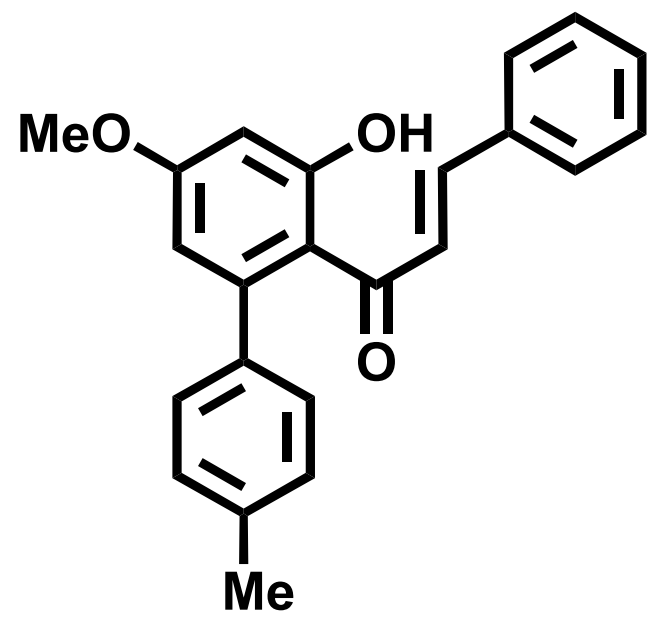	344.4	3	1	26	28	5	105.17	46.53	5.27	2100	64.41
**C13***	**3i**	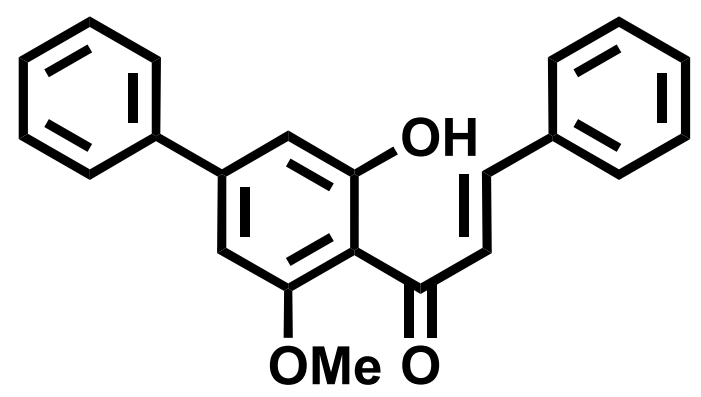	330.38	3	1	25	27	5	100.2	46.53	4.96	3000	67.09
**-**	**3j**	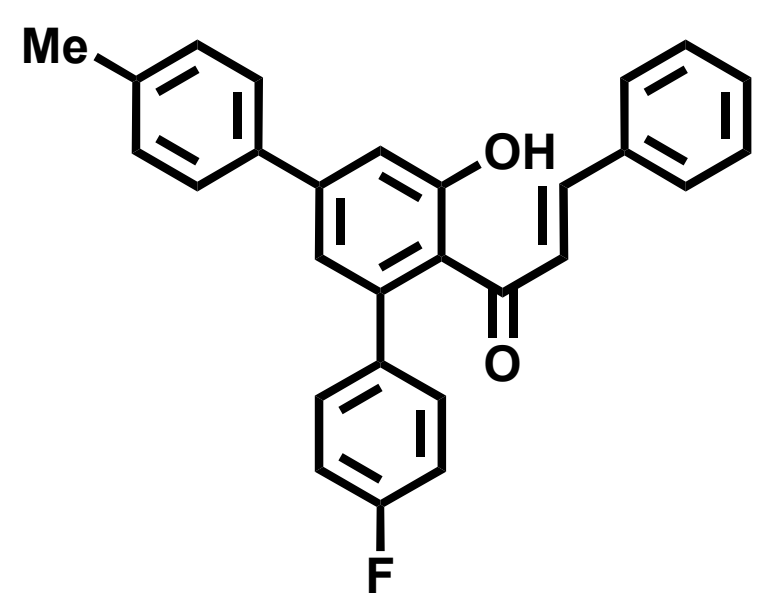	408.46	2	1	31	34	5	124.07	37.3	7.07	1000	53.23

MW: Molecular Weight (g/mol); **HBA:** Hydrogen Bond Acceptors; **HBD:** Hydrogen Bond Donors; **nAtoms:** Number of Atoms; **nBonds:** Number of Bonds; **nRotB:** Number of Rotatable Bonds; **MR:** Molar Refractivity; **TPSA:** Topological Polar Surface Area (Å^2); **LogP:** Octanol-water partition coefficient; **Predicted L*D50*:** Lethal Dose for 50% of the population; **ASS:** Average Structural Similarity.

(*) The parameters MW, HBA, HBD, nAtoms, and nBonds for these molecules have been previously reported in a publication by "P.Q. Monteiro, E. Schaeffer, A.J.M. da Silva, C.R. Alves, F. Souza-Silva, A Virtual Screening Approach to Evaluate the Multitarget Potential of a Chalcone Library with Binding Properties to Oligopeptidase B and Cysteine Proteinase B from *Leishmania (Viannia) braziliensis*, Int. J. Mol. Sci. 26 (2025) 2025.doi: 10.3390/ijms26052025".

The correspondence between the compound identifiers (ID), whose structures were previously reported (Monteiro et al., 2025), and the structures evaluated in this study is presented.

Supplementary File

2. Synthesis of Chalcone 2′,4′,6′-trimethoxy-chalcone (TMC)

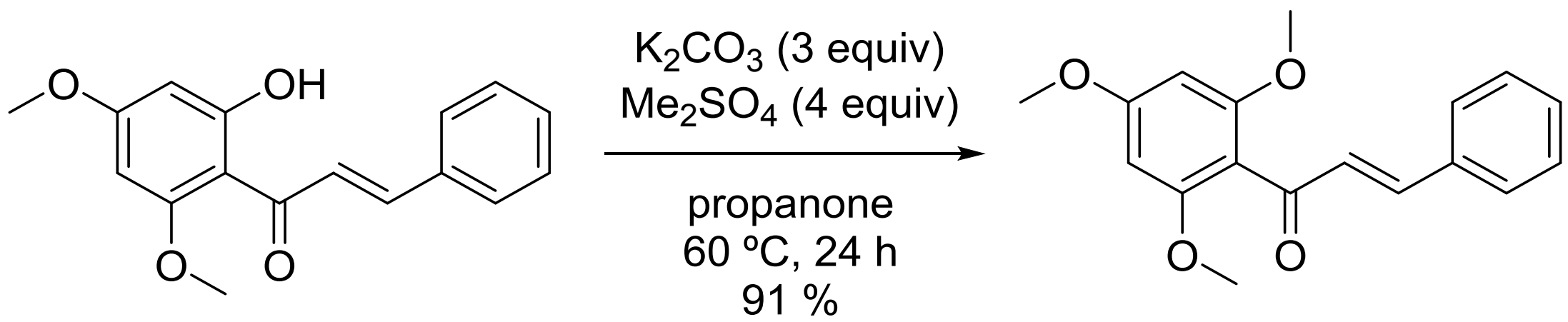

Flavokawain B (3b) **TMC**

Figure S1. Scheme of synthesis of chalcone TMC

A solution of **3b/*Flavokawain B*** (0.284 g, 1 mmol) and K_2CO_3 (0.414 g, 3 mmol) in acetone (HPLC grade, 7,5 mL) was stirred at room temperature for 30 minutes. Then Me_2SO_4 (0.4 mL, 4 mmol) was slowly added, and the reaction mixture was stirred at reflux for 24 h. The resulting reaction mixture was allowed to cool to room temperature, quenched with NH_4OH (2,5 mL of 10% solution in water) and water (25 mL) was added. Resulting suspension was extracted with ethyl acetate (3 x 50 mL) and the layers were reunited, washed with brine followed by drying over anhydrous sodium sulfate and concentrating under reduced pressure to furnish the crude product. The residue obtained was purified by flash column chromatography using hexanes-ethyl acetate (20%) as an eluent to afford chalcone **C109** (0.247 g, 91%) as a yellowish solid. The structural assignment of the product coincide with the previous report [1]. ^{1}H and ^{13}C NMR spectra are included below (Supplementary Fig. S46).

^{1}H NMR (400 MHz, $CDCl_3$) δ 7.54 – 7.50 (m, 2H), 7.39 – 7.33 (m, 4H), 6.96 (d, *J* = 16.1 Hz, 1H), 6.16 (s, 2H), 3.86 (s, 3H), 3.77 (s, 6H). ^{13}C NMR (101 MHz, $CDCl_3$) δ 194.28, 162.44, 158.87, 144.10, 135.03, 130.17, 129.04, 128.80, 128.37, 111.79, 90.73, 55.94, 55.48.

[1] Burmaoglu S, Algul O, Anil DA, Gobek A, Duran GG, Ersan RH, Duran N. Synthesis and anti-proliferative activity of fluoro-substituted chalcones. Bioorg Med Chem Lett. 2016; 26(13):3172-6. doi: 10.1016/j.bmcl.2016.04.096.

Supplementary File

3. High-resolution ESI-Qq-TOF of Newly Synthesized Compounds

Figure S2. High-resolution ESI-Qq-TOF mass spectrum of **2c**

Compound Spectrum SmartFormula Report

Analysis Info

Analysis Name	D:\Data\2025\IOC\CARLOS ALVES\252983 - ESY046.d	Acquisition Date	7/22/2025 9:46:52 AM
Method	Tune_pos_Standard.m	Operator	Vinicius Nery
Sample Name	ESY046	Instrument	compact 8255754.10035
Comment			

Acquisition Parameter

Source Type	ESI	Ion Polarity	Positive	Set Nebulizer	0.4 Bar
Focus	Not active	Set Capillary	4000 V	Set Dry Heater	200 °C
Scan Begin	100 m/z	Set End Plate Offset	-400 V	Set Dry Gas	4.0 l/min
Scan End	1000 m/z	Set Charging Voltage	2000 V	Set Divert Valve	Source
		Set Corona	0 nA	Set APCI Heater	0 °C

+MS, 0.1-0.2min #3-13

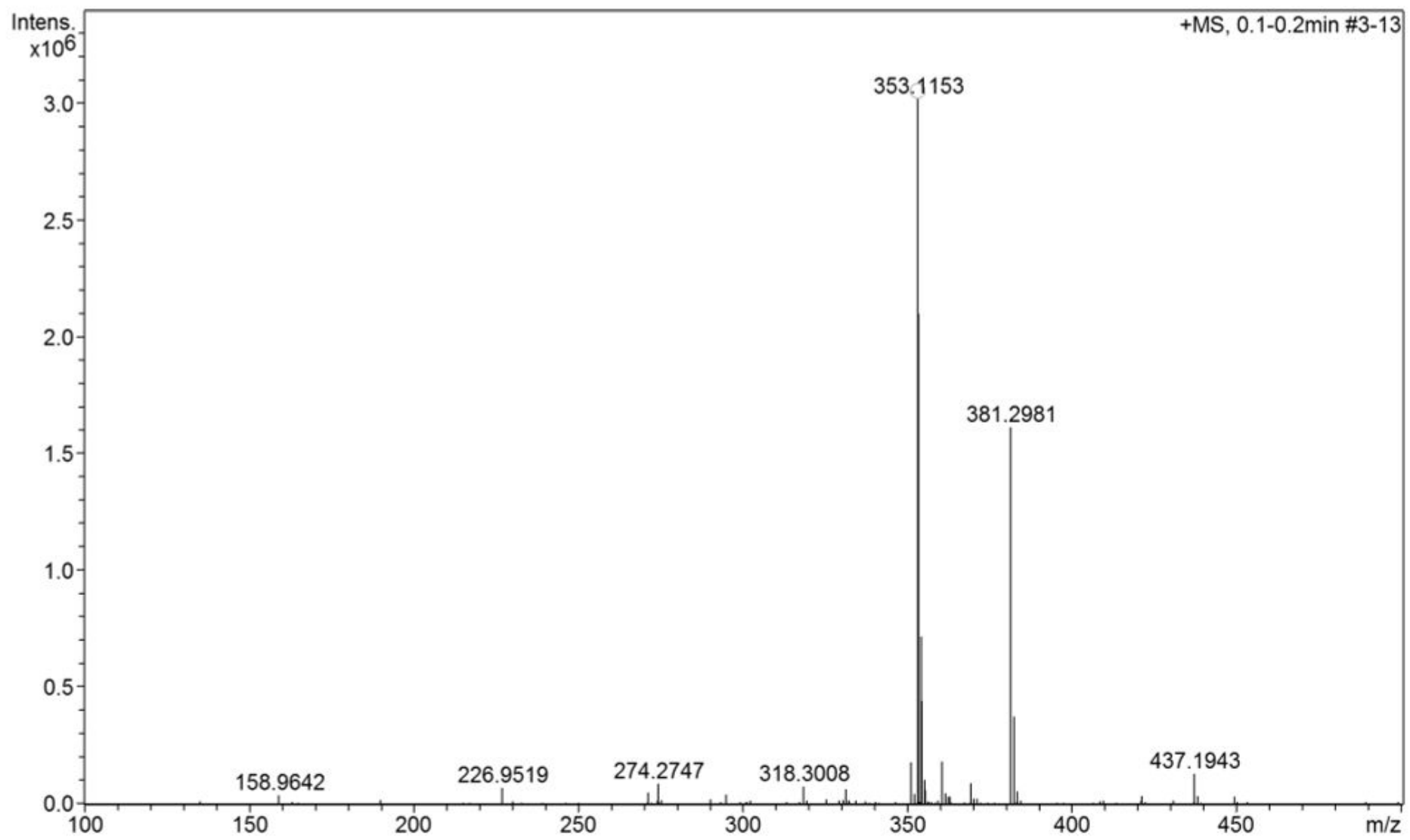

Meas. m/z	#	Ion Formula	m/z	err [ppm]	Mean err [ppm]	rdb	N-Rule	e⁻ Conf	mSigma	Std I	Std Mean m/z	Std I VarNorm	Std m/z Diff	Std Comb Dev
353.115298	1	C20H13N6O	353.114535	-2.2	-3.8	17.5	ok	even	2.8	4.2	n.a.	n.a.	n.a.	n.a.
	1	C22H18NaO3	353.114815	-1.4	-2.1	13.5	ok	even	1.9	2.9	n.a.	n.a.	n.a.	n.a.

252983 - ESY046.d
Bruker Compass DataAnalysis 4.2 printed: 7/22/2025 10:50:01 AM by: Vinicius Nery Page 1 of 1

Supplementary File

Figure S3. High-resolution ESI-Qq-TOF mass spectrum of **2d**

Compound Spectrum SmartFormula Report

Analysis Info

Analysis Name	D:\Data\2025\IOC\CARLOS ALVES\252984 - ESY047.d	Acquisition Date	7/22/2025 9:48:43 AM
Method	Tune_pos_Standard.m	Operator	Vinicius Nery
Sample Name	ESY047	Instrument	compact 8255754.10035
Comment			

Acquisition Parameter

Source Type	ESI	Ion Polarity	Positive	Set Nebulizer	0.4 Bar
Focus	Not active	Set Capillary	4000 V	Set Dry Heater	200 °C
Scan Begin	100 m/z	Set End Plate Offset	-400 V	Set Dry Gas	4.0 l/min
Scan End	1000 m/z	Set Charging Voltage	2000 V	Set Divert Valve	Source
		Set Corona	0 nA	Set APCI Heater	0 °C

+MS, 0.1-0.4min #5-25

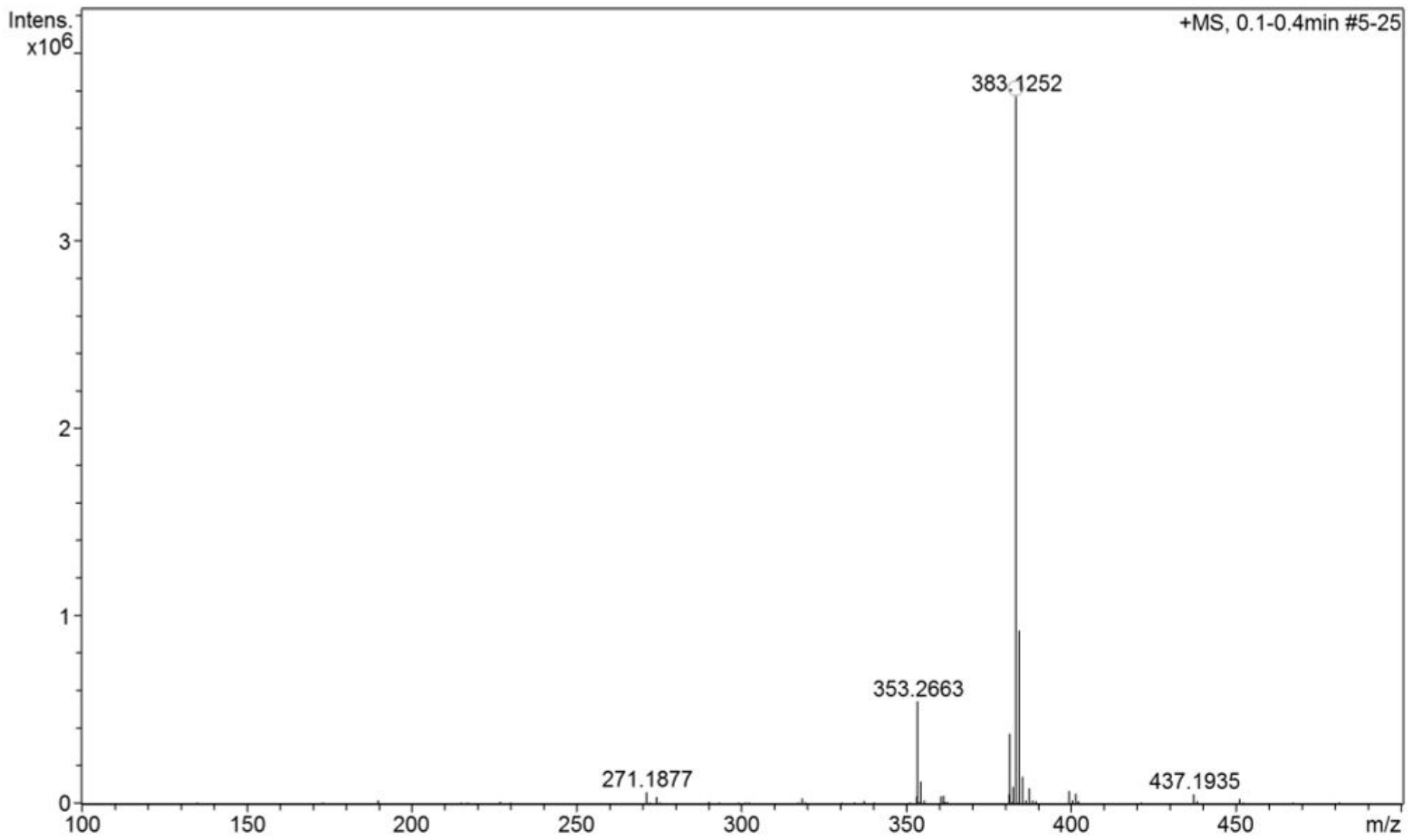

Meas. m/z	#	Ion Formula	m/z	err [ppm]	Mean err [ppm]	rdb	N-Rule	e⁻ Conf	mSigma	Std I	Std Mean m/z	Std I VarNorm	Std m/z Diff	Std Comb Dev
383.125223	1	C21H15N6O2	383.125100	-0.3	-1.7	17.5	ok	even	3.9	5.2	n.a.	n.a.	n.a.	n.a.
	1	C23H20NaO4	383.125380	0.4	-0.2	13.5	ok	even	4.0	6.3	n.a.	n.a.	n.a.	n.a.

252984 - ESY047.d
Bruker Compass DataAnalysis 4.2 printed: 7/22/2025 9:50:25 AM by: Vinicius Nery Page 1 of 1

Figure S4. High-resolution ESI-Qq-TOF mass spectrum of **2e**

Compound Spectrum SmartFormula Report

Analysis Info

Analysis Name	D:\Data\2025\IOC\CARLOS ALVES\252986 - ESY049.d	Acquisition Date	7/22/2025 9:54:55 AM
Method	Tune_pos_Standard.m	Operator	Vinicius Nery
Sample Name	ESY049	Instrument	compact 8255754.10035
Comment			

Acquisition Parameter

Source Type	ESI	Ion Polarity	Positive	Set Nebulizer	0.4 Bar
Focus	Not active	Set Capillary	4000 V	Set Dry Heater	200 °C
Scan Begin	100 m/z	Set End Plate Offset	-400 V	Set Dry Gas	4.0 l/min
Scan End	1000 m/z	Set Charging Voltage	2000 V	Set Divert Valve	Source
		Set Corona	0 nA	Set APCI Heater	0 °C

+MS, 0.2-0.9min #13-51

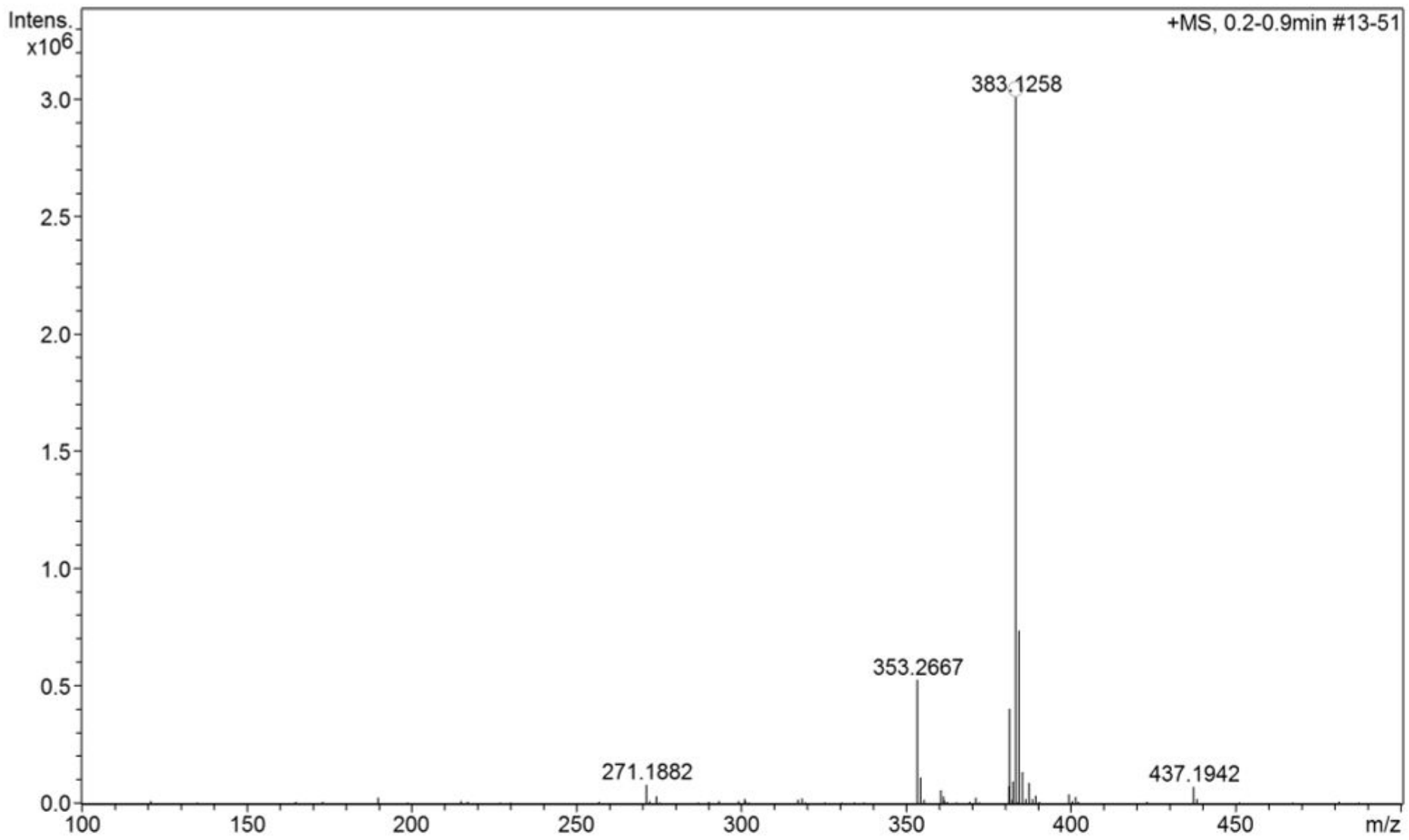

Meas. m/z	#	Ion Formula	m/z	err [ppm]	Mean err [ppm]	rdb	N-Rule	e⁻ Conf	mSigma	Std I	Std Mean m/z	Std I VarNorm	Std m/z Diff	Std Comb Dev
383.125809	1	C23H20NaO4	383.125380	-1.1	-2.5	13.5	ok	even	5.4	7.2	n.a.	n.a.	n.a.	n.a.
	2	C24H16N4Na	383.126717	2.4	0.7	18.5	ok	even	18.3	25.0	n.a.	n.a.	n.a.	n.a.

252986 - ESY049.d
Bruker Compass DataAnalysis 4.2 printed: 7/22/2025 9:57:07 AM by: Vinicius Nery Page 1 of 1

Supplementary File

Figure S5. High-resolution ESI-Qq-TOF mass spectrum of **2f**

Compound Spectrum SmartFormula Report

Analysis Info

Analysis Name	D:\Data\2025\IOC\CARLOS ALVES\252985 - ESY048.d	Acquisition Date	7/22/2025 9:51:26 AM
Method	Tune_pos_Standard.m	Operator	Vinicius Nery
Sample Name	ESY048	Instrument	compact 8255754.10035
Comment			

Acquisition Parameter

Source Type	ESI	Ion Polarity	Positive	Set Nebulizer	0.4 Bar
Focus	Not active	Set Capillary	4000 V	Set Dry Heater	200 °C
Scan Begin	100 m/z	Set End Plate Offset	-400 V	Set Dry Gas	4.0 l/min
Scan End	1000 m/z	Set Charging Voltage	2000 V	Set Divert Valve	Source
		Set Corona	0 nA	Set APCI Heater	0 °C

+MS, 0.2-1.0min #13-58

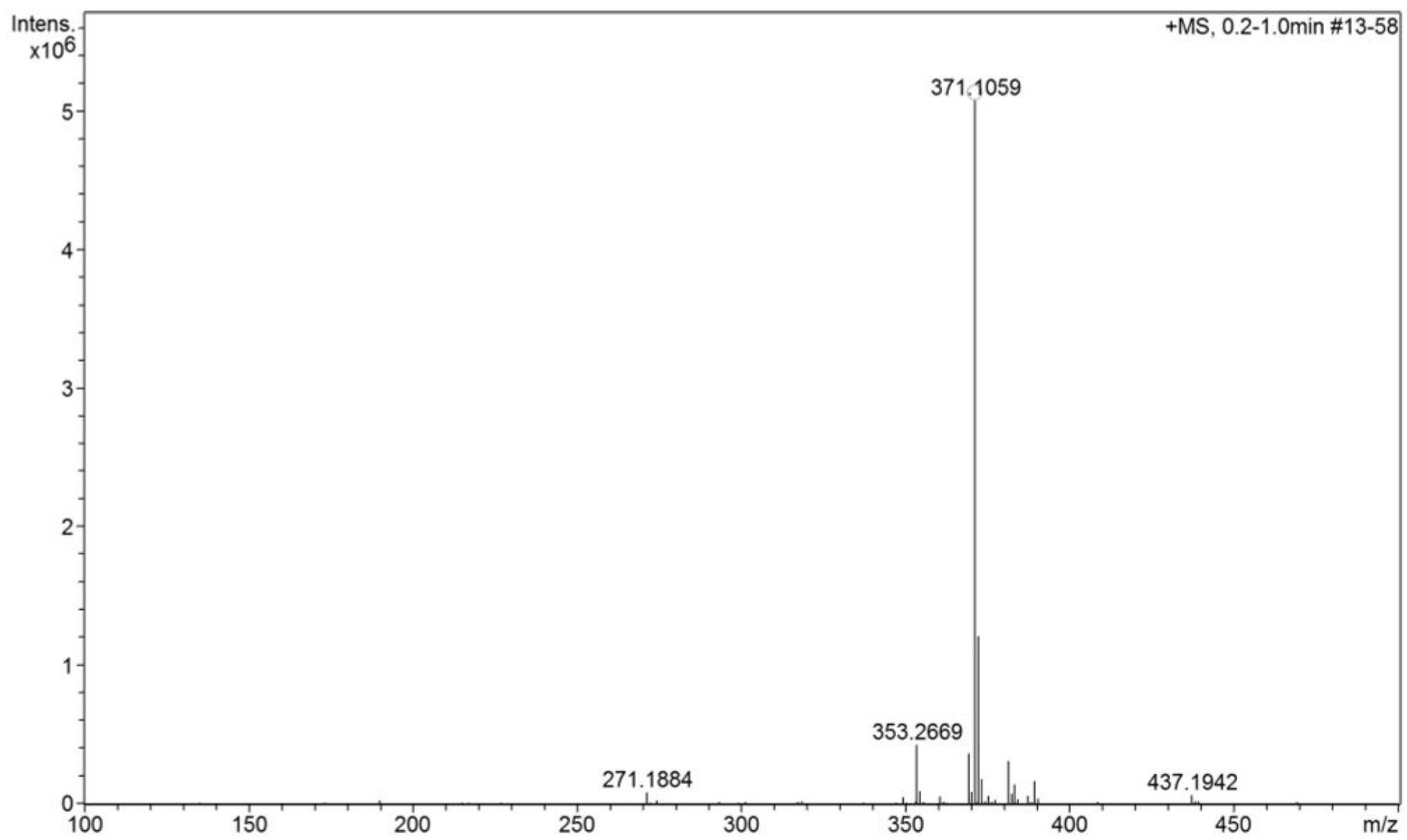

Meas. m/z	#	Ion Formula	m/z	err [ppm]	Mean err [ppm]	rdb	N-Rule	e⁻ Conf	mSigma	Std I	Std Mean m/z	Std I VarNorm	Std m/z Diff	Std Comb Dev
371.105853	1	C22H17FNaO3	371.105393	-1.2	-2.1	13.5	ok	even	1.4	2.0	n.a.	n.a.	n.a.	n.a.

252985 - ESY048.d
Bruker Compass DataAnalysis 4.2 printed: 7/22/2025 9:53:47 AM by: Vinicius Nery Page 1 of 1

Supplementary File

Figure S6. High-resolution ESI-Qq-TOF mass spectrum of **2g**

Compound Spectrum SmartFormula Report

Analysis Info

Analysis Name	D:\Data\2025\IOC\CARLOS ALVES\252988 - ESY051.d	Acquisition Date	7/22/2025 9:59:20 AM
Method	Tune_pos_Standard.m	Operator	Vinicius Nery
Sample Name	ESY051	Instrument	compact 8255754.10035
Comment			

Acquisition Parameter

Source Type	ESI	Ion Polarity	Positive	Set Nebulizer	0.4 Bar
Focus	Not active	Set Capillary	4000 V	Set Dry Heater	200 °C
Scan Begin	100 m/z	Set End Plate Offset	-400 V	Set Dry Gas	4.0 l/min
Scan End	1000 m/z	Set Charging Voltage	2000 V	Set Divert Valve	Source
		Set Corona	0 nA	Set APCI Heater	0 °C

+MS, 0.1-1.0min #6-59

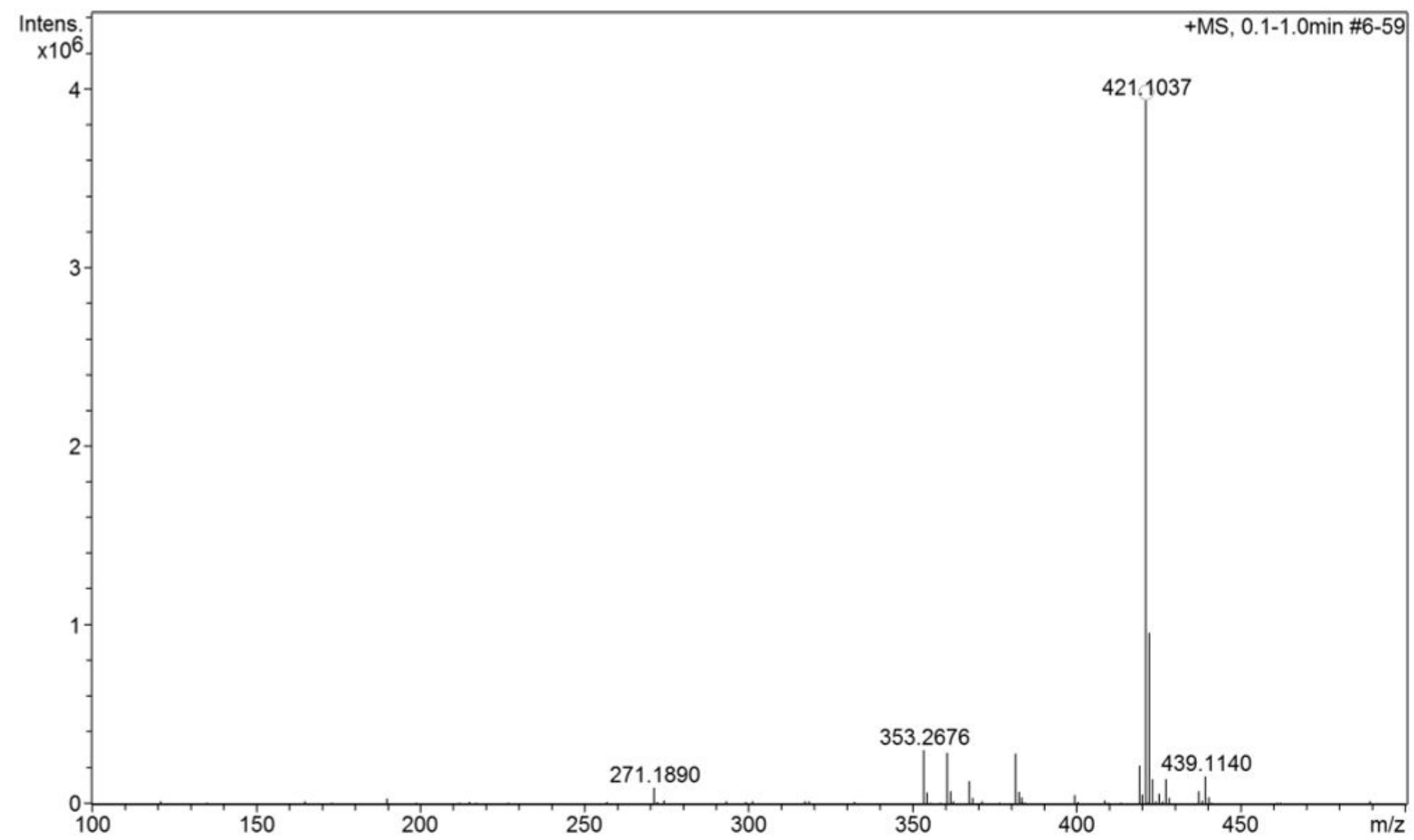

Meas. m/z	#	Ion Formula	m/z	err [ppm]	Mean err [ppm]	rdb	N-Rule	e⁻ Conf	mSigma	Std I	Std Mean m/z	Std I VarNorm	Std m/z Diff	Std Comb Dev
421.103685	1	C23H17F3NaO3	421.102200	-3.5	-4.2	13.5	ok	even	4.6	7.5	n.a.	n.a.	n.a.	n.a.

252988 - ESY051.d
Bruker Compass DataAnalysis 4.2 printed: 7/22/2025 10:00:48 AM by: Vinicius Nery Page 1 of 1

Figure S7. High-resolution ESI-Qq-TOF mass spectrum of **2h**

Compound Spectrum SmartFormula Report

Analysis Info

Analysis Name	D:\Data\2025\IOC\CARLOS ALVES\252987 - ESY050.d	Acquisition Date	7/22/2025 9:56:33 AM
Method	Tune_pos_Standard.m	Operator	Vinicius Nery
Sample Name	ESY050	Instrument	compact 8255754.10035
Comment			

Acquisition Parameter

Source Type	ESI	Ion Polarity	Positive	Set Nebulizer	0.4 Bar
Focus	Not active	Set Capillary	4000 V	Set Dry Heater	200 °C
Scan Begin	100 m/z	Set End Plate Offset	-400 V	Set Dry Gas	4.0 l/min
Scan End	1000 m/z	Set Charging Voltage	2000 V	Set Divert Valve	Source
		Set Corona	0 nA	Set APCI Heater	0 °C

+MS, 0.2-1.0min #9-59

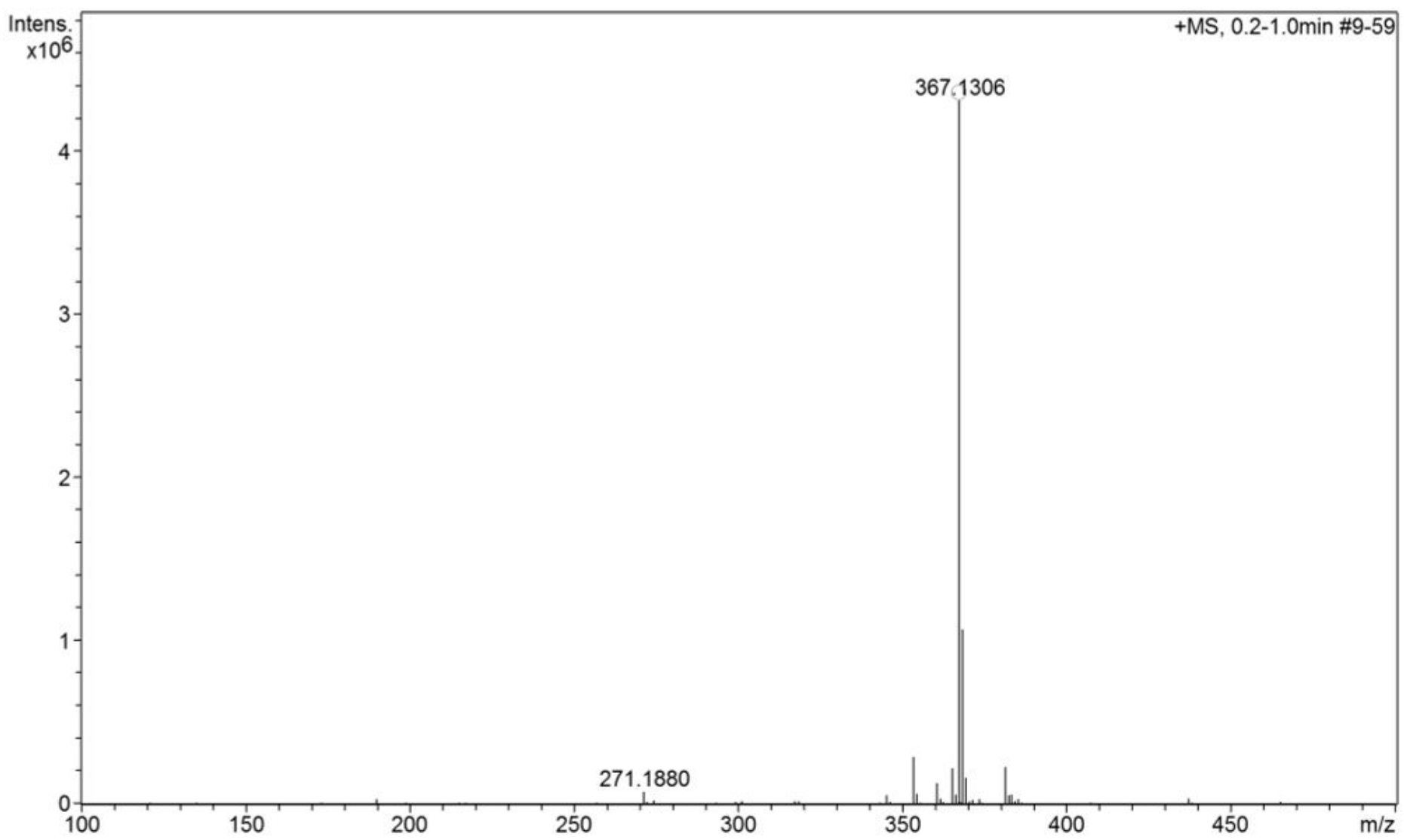

Meas. m/z	#	Ion Formula	m/z	err [ppm]	Mean err [ppm]	rdb	N-Rule	e⁻ Conf	mSigma	Std I	Std Mean m/z	Std I VarNorm	Std m/z Diff	Std Comb Dev
367.130633	1	C23H20NaO3	367.130465	-0.5	-1.2	13.5	ok	even	2.1	3.0	n.a.	n.a.	n.a.	n.a.

252987 - ESY050.d
Bruker Compass DataAnalysis 4.2 printed: 7/22/2025 9:57:57 AM by: Vinicius Nery Page 1 of 1

Supplementary File

Figure S8. High-resolution ESI-Qq-TOF mass spectrum of **2i**

Compound Spectrum SmartFormula Report

Analysis Info

Analysis Name	D:\Data\2025\IOC\CARLOS ALVES\252989 - ESY052.d	Acquisition Date	7/22/2025 10:02:05 AM
Method	Tune_pos_Standard.m	Operator	Vinicius Nery
Sample Name	ESY052	Instrument	compact 8255754.10035
Comment			

Acquisition Parameter

Source Type	ESI	Ion Polarity	Positive	Set Nebulizer	0.4 Bar
Focus	Not active	Set Capillary	4000 V	Set Dry Heater	200 °C
Scan Begin	100 m/z	Set End Plate Offset	-400 V	Set Dry Gas	4.0 l/min
Scan End	1000 m/z	Set Charging Voltage	2000 V	Set Divert Valve	Source
		Set Corona	0 nA	Set APCI Heater	0 °C

+MS, 0.3-1.0min #20-59

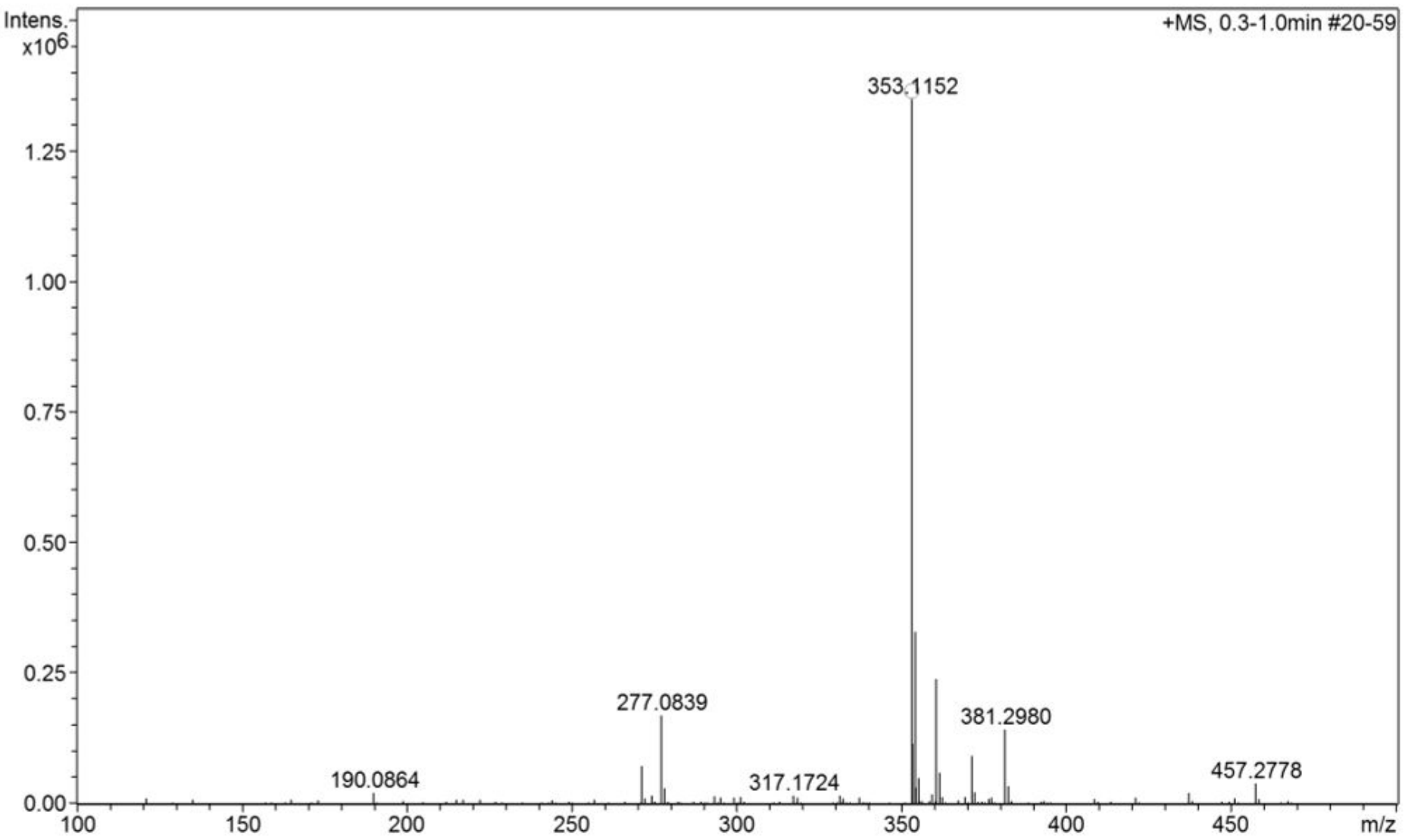

Meas. m/z	#	Ion Formula	m/z	err [ppm]	Mean err [ppm]	rdb	N-Rule	e⁻ Conf	mSigma	Std I	Std Mean m/z	Std I VarNorm	Std m/z Diff	Std Comb Dev
353.115230	1	C22H18NaO3	353.114815	-1.2	-1.8	13.5	ok	even	2.4	4.4	n.a.	n.a.	n.a.	n.a.

252989 - ESY052.d
Bruker Compass DataAnalysis 4.2 printed: 7/22/2025 10:03:33 AM by: Vinicius Nery Page 1 of 1

Figure S9. High-resolution ESI-Qq-TOF mass spectrum of **2j**

Compound Spectrum SmartFormula Report

Analysis Info

Analysis Name	D:\Data\2025\IOC\CARLOS ALVES\252990 - ESY057.d	Acquisition Date	7/22/2025 10:04:36 AM
Method	Tune_pos_Standard.m	Operator	Vinicius Nery
Sample Name	ESY057	Instrument	compact 8255754.10035
Comment			

Acquisition Parameter

Source Type	ESI	Ion Polarity	Positive	Set Nebulizer	0.4 Bar
Focus	Not active	Set Capillary	4000 V	Set Dry Heater	200 °C
Scan Begin	100 m/z	Set End Plate Offset	-400 V	Set Dry Gas	4.0 l/min
Scan End	1000 m/z	Set Charging Voltage	2000 V	Set Divert Valve	Source
		Set Corona	0 nA	Set APCI Heater	0 °C

+MS, 0.3-1.0min #15-56

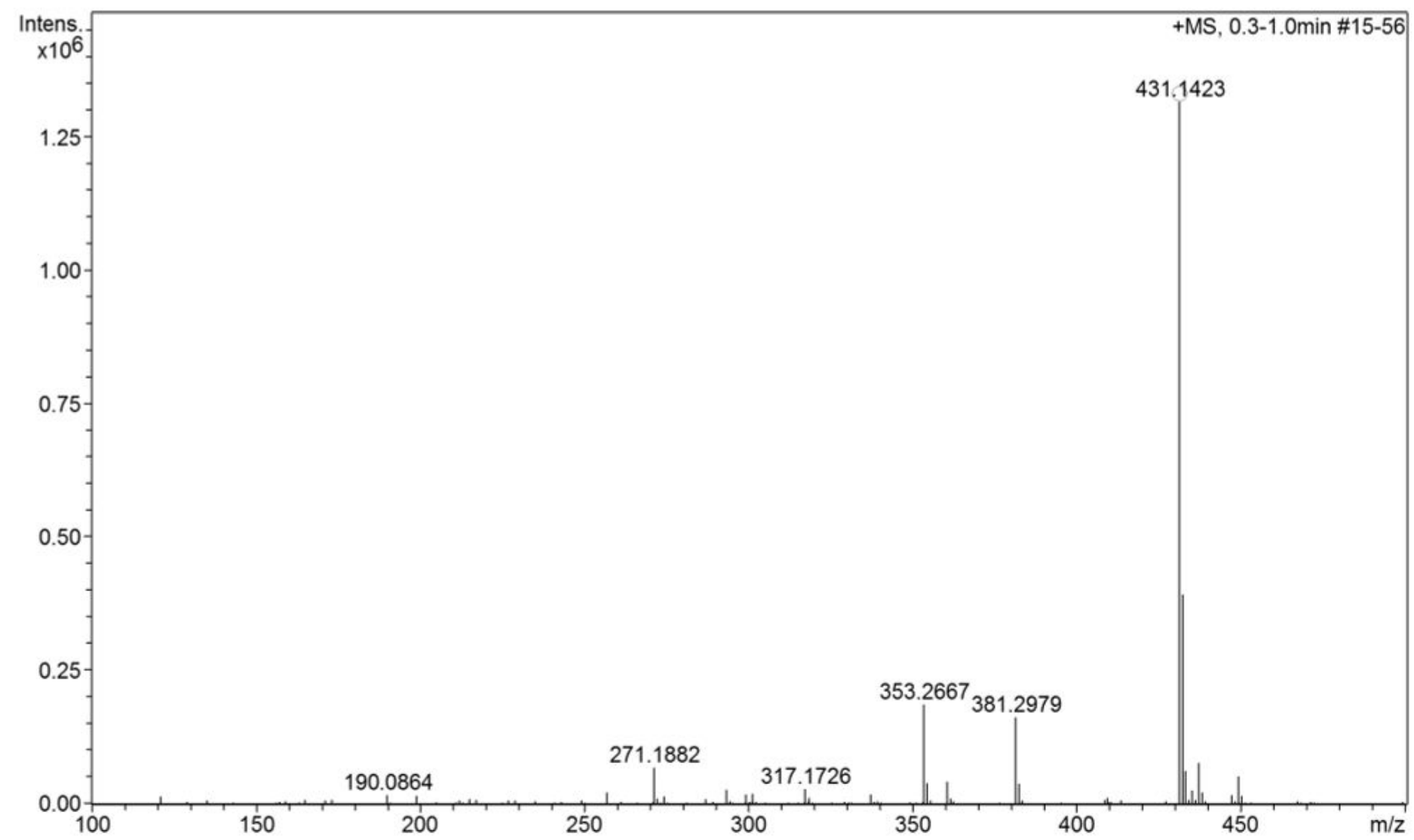

Meas. m/z	#	Ion Formula	m/z	err [ppm]	Mean err [ppm]	rdb	N-Rule	e⁻ Conf	mSigma	Std I	Std Mean m/z	Std I VarNorm	Std m/z Diff	Std Comb Dev
431.142330	1	$C_{28}H_{21}FNaO_2$	431.141779	-1.3	-1.4	17.5	ok	even	4.5	6.8	n.a.	n.a.	n.a.	n.a.

252990 - ESY057.d

Bruker Compass DataAnalysis 4.2 printed: 7/22/2025 10:06:55 AM by: Vinicius Nery Page 1 of 1

Supplementary File

Figure S10. High-resolution ESI-Qq-TOF mass spectrum of **3c**

Compound Spectrum SmartFormula Report

Analysis Info

Analysis Name	D:\Data\2025\IOC\CARLOS ALVES\252991 - ESY058.d	Acquisition Date	7/22/2025 10:08:49 AM
Method	Tune_pos_Standard.m	Operator	Vinicius Nery
Sample Name	ESY058	Instrument	compact 8255754.10035
Comment			

Acquisition Parameter

Source Type	ESI	Ion Polarity	Positive	Set Nebulizer	0.4 Bar
Focus	Not active	Set Capillary	4000 V	Set Dry Heater	200 °C
Scan Begin	100 m/z	Set End Plate Offset	-400 V	Set Dry Gas	4.0 l/min
Scan End	1000 m/z	Set Charging Voltage	2000 V	Set Divert Valve	Source
		Set Corona	0 nA	Set APCI Heater	0 °C

+MS, 0.2-1.0min #13-59

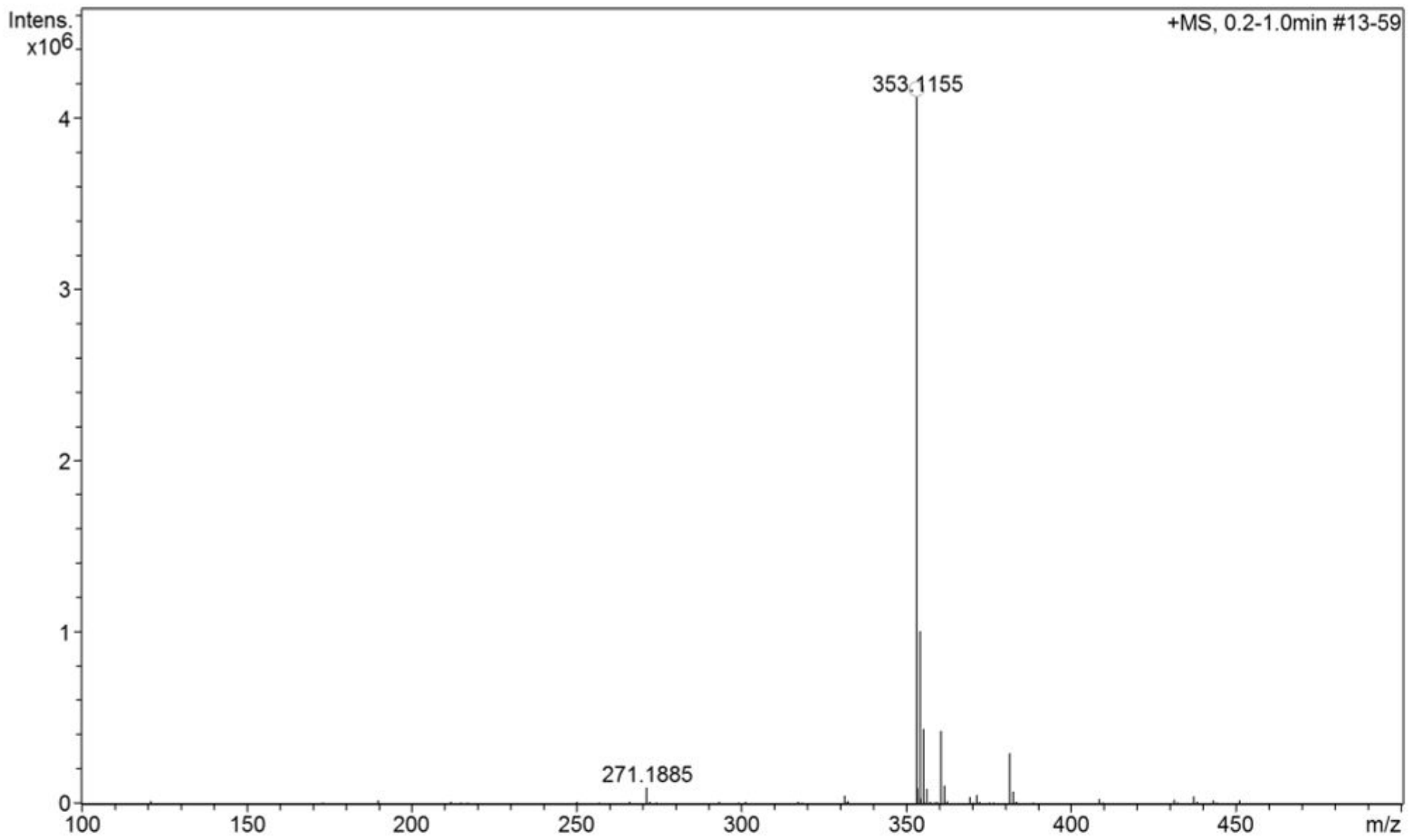

Meas. m/z	#	Ion Formula	m/z	err [ppm]	Mean err [ppm]	rdb	N-Rule	e⁻ Conf	mSigma	Std I	Std Mean m/z	Std I VarNorm	Std m/z Diff	Std Comb Dev
353.115498	1	$C_{22}H_{18}NaO_3$	353.114815	-1.9	-6.1	13.5	ok	even	41.2	67.7	n.a.	n.a.	n.a.	n.a.

252991 - ESY058.d
Bruker Compass DataAnalysis 4.2 printed: 7/22/2025 10:10:56 AM by: Vinicius Nery Page 1 of 1

Figure S11. High-resolution ESI-Qq-TOF mass spectrum of **3d**

Compound Spectrum SmartFormula Report

Analysis Info

Analysis Name	D:\Data\2025\IOC\CARLOS ALVES\252992 - ESY059.d	Acquisition Date	7/22/2025 10:10:36 AM
Method	Tune_pos_Standard.m	Operator	Vinicius Nery
Sample Name	ESY059	Instrument	compact 8255754.10035
Comment			

Acquisition Parameter

Source Type	ESI	Ion Polarity	Positive	Set Nebulizer	0.4 Bar
Focus	Not active	Set Capillary	4000 V	Set Dry Heater	200 °C
Scan Begin	100 m/z	Set End Plate Offset	-400 V	Set Dry Gas	4.0 l/min
Scan End	1000 m/z	Set Charging Voltage	2000 V	Set Divert Valve	Source
		Set Corona	0 nA	Set APCI Heater	0 °C

+MS, 0.3-1.0min #17-59

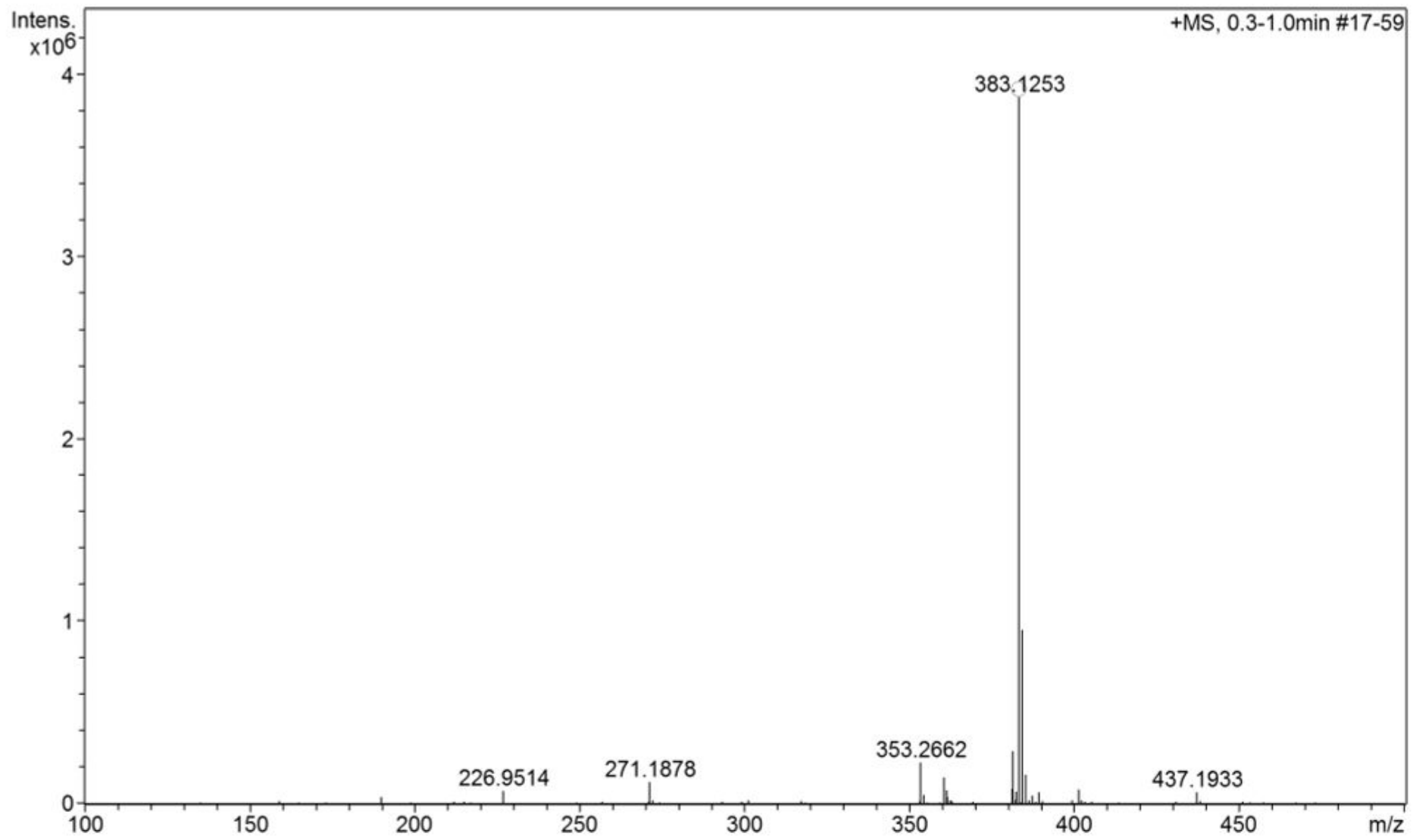

Meas. m/z	#	Ion Formula	m/z	err [ppm]	Mean err [ppm]	rdb	N-Rule	e⁻ Conf	mSigma	Std I	Std Mean m/z	Std I VarNorm	Std m/z Diff	Std Comb Dev
383.125266	1	C23H20NaO4	383.125380	0.3	-0.5	13.5	ok	even	3.7	5.1	n.a.	n.a.	n.a.	n.a.
	2	C24H16N4Na	383.126717	3.8	2.7	18.5	ok	even	17.2	24.4	n.a.	n.a.	n.a.	n.a.

252992 - ESY059.d

Bruker Compass DataAnalysis 4.2 printed: 7/22/2025 10:14:06 AM by: Vinicius Nery Page 1 of 1

Supplementary File

Figure S12. High-resolution ESI-Qq-TOF mass spectrum of **3e**

Compound Spectrum SmartFormula Report

Analysis Info

Analysis Name	D:\Data\2025\IOC\CARLOS ALVES\252994 - ESY061.d	Acquisition Date	7/22/2025 10:16:57 AM
Method	Tune_pos_Standard.m	Operator	Vinicius Nery
Sample Name	ESY061	Instrument	compact 8255754.10035
Comment			

Acquisition Parameter

Source Type	ESI	Ion Polarity	Positive	Set Nebulizer	0.4 Bar
Focus	Not active	Set Capillary	4000 V	Set Dry Heater	200 °C
Scan Begin	100 m/z	Set End Plate Offset	-400 V	Set Dry Gas	4.0 l/min
Scan End	1000 m/z	Set Charging Voltage	2000 V	Set Divert Valve	Source
		Set Corona	0 nA	Set APCI Heater	0 °C

+MS, 0.3-1.0min #15-59

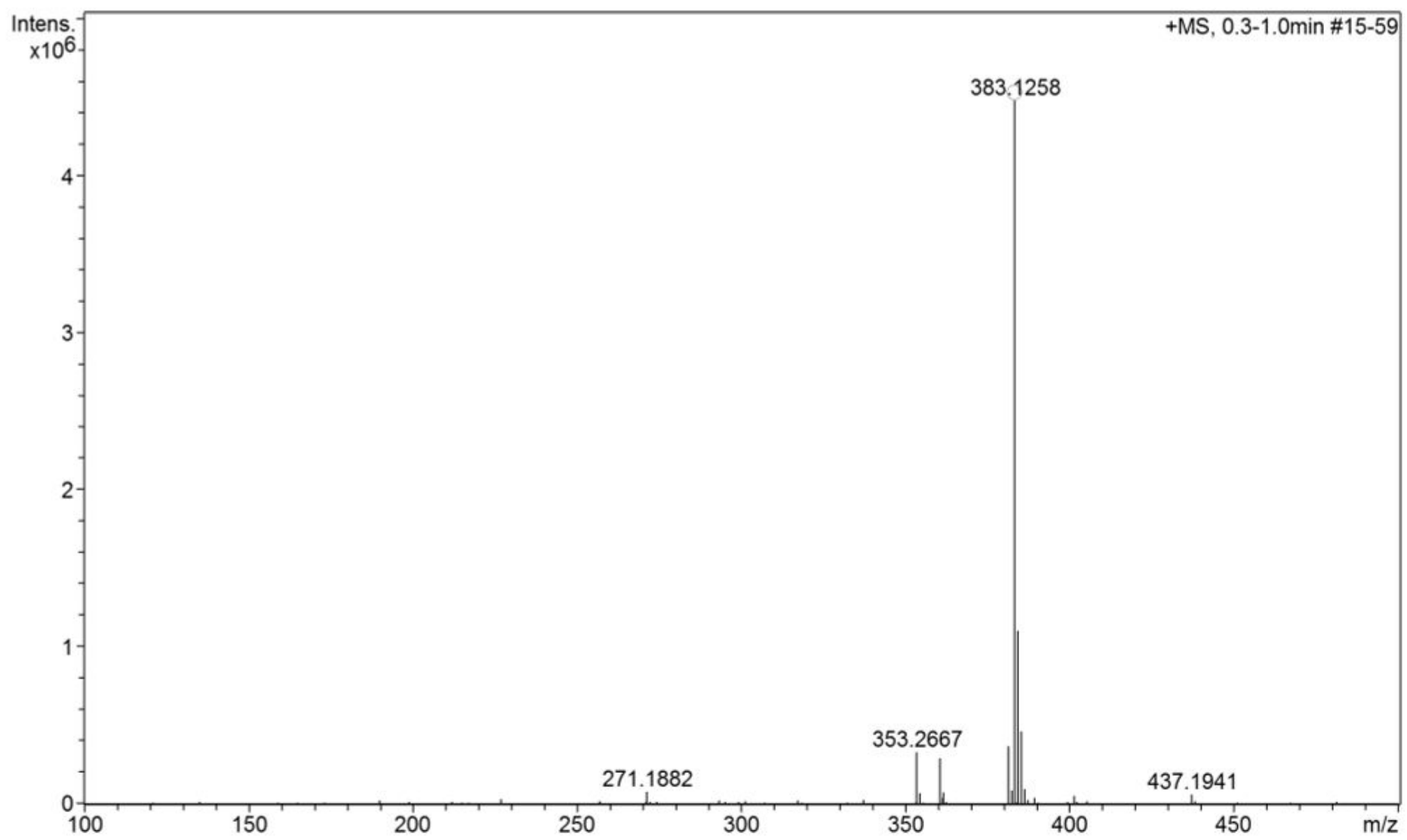

Meas. m/z	#	Ion Formula	m/z	err [ppm]	Mean err [ppm]	rdb	N-Rule	e⁻ Conf	mSigma	Std I	Std Mean m/z	Std I VarNorm	Std m/z Diff	Std Comb Dev
383.125773	1	C23H20NaO4	383.125380	-1.0	-4.7	13.5	ok	even	36.9	58.0	n.a.	n.a.	n.a.	n.a.
	2	C24H16N4Na	383.126717	2.5	-1.5	18.5	ok	even	41.5	59.0	n.a.	n.a.	n.a.	n.a.

252994 - ESY061.d

Bruker Compass DataAnalysis 4.2 printed: 7/22/2025 10:18:31 AM by: Vinicius Nery Page 1 of 1

Supplementary File

Figure S13. High-resolution ESI-Qq-TOF mass spectrum of **3f**

Compound Spectrum SmartFormula Report

Analysis Info

Analysis Name	D:\Data\2025\IOC\CARLOS ALVES\252993 - ESY060.d	Acquisition Date	7/22/2025 10:13:34 AM
Method	Tune_pos_Standard.m	Operator	Vinicius Nery
Sample Name	ESY060	Instrument	compact 8255754.10035
Comment			

Acquisition Parameter

Source Type	ESI	Ion Polarity	Positive	Set Nebulizer	0.4 Bar
Focus	Not active	Set Capillary	4000 V	Set Dry Heater	200 °C
Scan Begin	100 m/z	Set End Plate Offset	-400 V	Set Dry Gas	4.0 l/min
Scan End	1000 m/z	Set Charging Voltage	2000 V	Set Divert Valve	Source
		Set Corona	0 nA	Set APCI Heater	0 °C

+MS, 0.1-1.0min #8-56

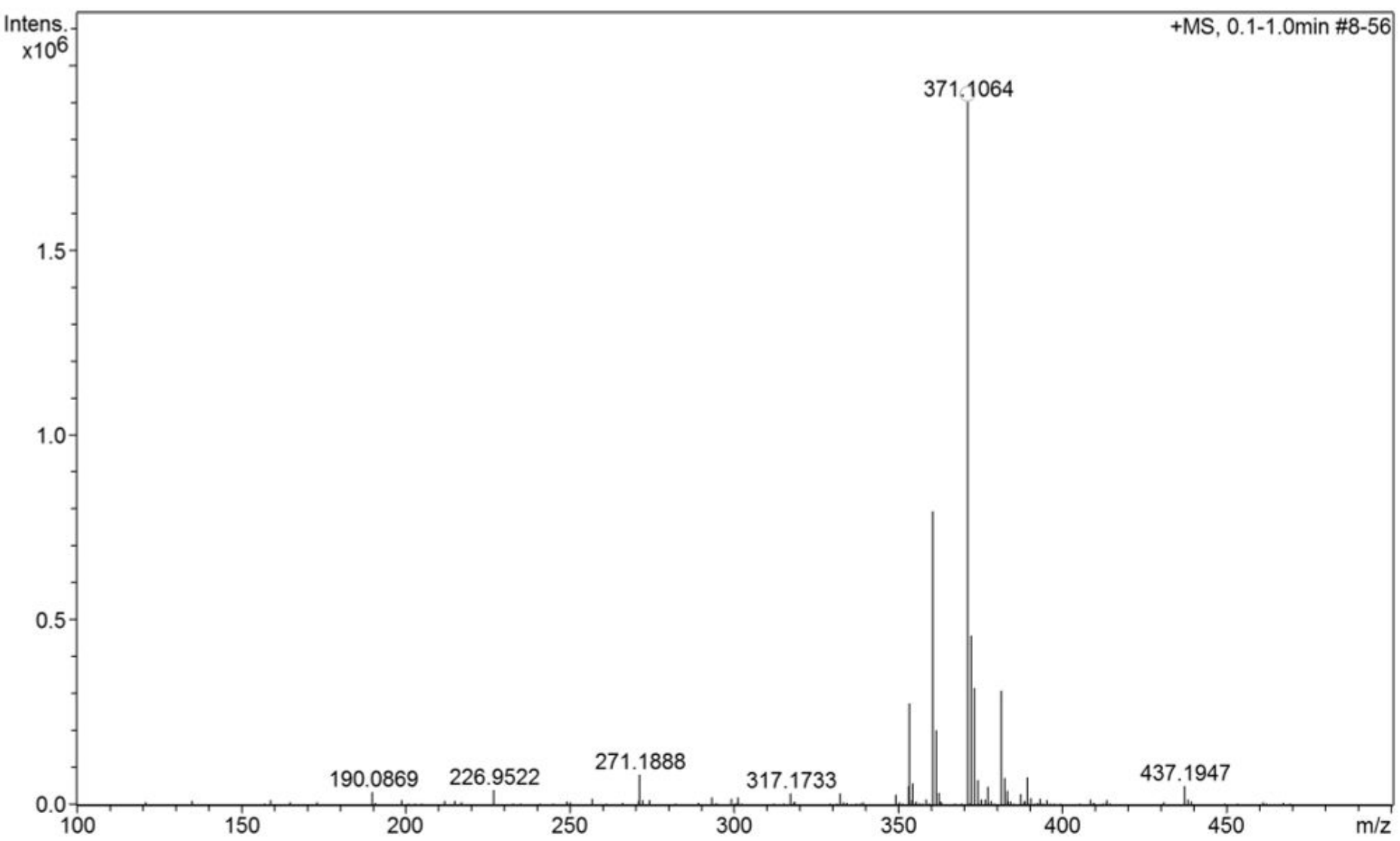

Meas. m/z	#	Ion Formula	m/z	err [ppm]	Mean err [ppm]	rdb	N-Rule	e⁻ Conf	mSigma	Std I	Std Mean m/z	Std I VarNorm	Std m/z Diff	Std Comb Dev
371.106397	1	C22H17FNaO3	371.105393	-2.7	-7.3	13.5	ok	even	76.2	118.7	n.a.	n.a.	n.a.	n.a.

252993 - ESY060.d
Bruker Compass DataAnalysis 4.2 printed: 7/22/2025 10:15:08 AM by: Vinicius Nery Page 1 of 1

Supplementary File

Figure S14. High-resolution ESI-Qq-TOF mass spectrum of **3g**

Compound Spectrum SmartFormula Report

Analysis Info

Analysis Name	D:\Data\2025\IOC\CARLOS ALVES\252996 - ESY063.d	Acquisition Date	7/22/2025 10:21:38 AM
Method	Tune_pos_Standard.m	Operator	Vinicius Nery
Sample Name	ESY063	Instrument	compact 8255754.10035
Comment			

Acquisition Parameter

Source Type	ESI	Ion Polarity	Positive	Set Nebulizer	0.4 Bar
Focus	Not active	Set Capillary	4000 V	Set Dry Heater	200 °C
Scan Begin	100 m/z	Set End Plate Offset	-400 V	Set Dry Gas	4.0 l/min
Scan End	1000 m/z	Set Charging Voltage	2000 V	Set Divert Valve	Source
		Set Corona	0 nA	Set APCI Heater	0 °C

+MS, 0.4-1.0min #22-59

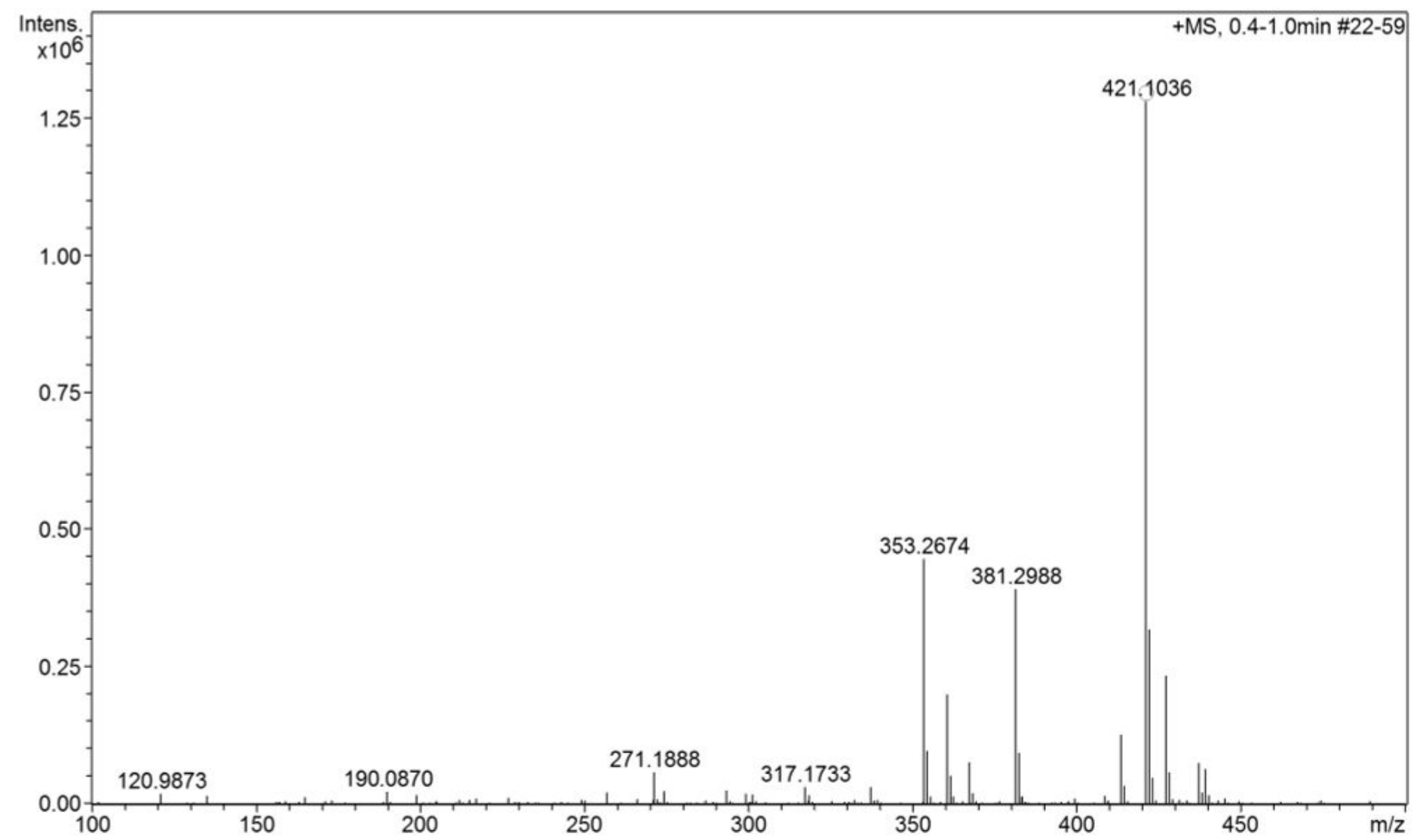

Meas. m/z	#	Ion Formula	m/z	err [ppm]	Mean err [ppm]	rdb	N-Rule	e⁻ Conf	mSigma	Std I	Std Mean m/z	Std I VarNorm	Std m/z Diff	Std Comb Dev
421.103570	1	C23H17F3NaO3	421.102200	-3.3	-3.8	13.5	ok	even	2.1	2.9	n.a.	n.a.	n.a.	n.a.

252996 - ESY063.d
Bruker Compass DataAnalysis 4.2 printed: 7/22/2025 10:27:50 AM by: Vinicius Nery Page 1 of 1

Supplementary File

Figure S15. High-resolution ESI-Qq-TOF mass spectrum of **3h**

Compound Spectrum SmartFormula Report

Analysis Info

Analysis Name	D:\Data\2025\IOC\CARLOS ALVES\252995 - ESY062.d	Acquisition Date	7/22/2025 10:19:35 AM
Method	Tune_pos_Standard.m	Operator	Vinicius Nery
Sample Name	ESY062	Instrument	compact 8255754.10035
Comment			

Acquisition Parameter

Source Type	ESI	Ion Polarity	Positive	Set Nebulizer	0.4 Bar
Focus	Not active	Set Capillary	4000 V	Set Dry Heater	200 °C
Scan Begin	100 m/z	Set End Plate Offset	-400 V	Set Dry Gas	4.0 l/min
Scan End	1000 m/z	Set Charging Voltage	2000 V	Set Divert Valve	Source
		Set Corona	0 nA	Set APCI Heater	0 °C

+MS, 0.3-0.9min #17-53

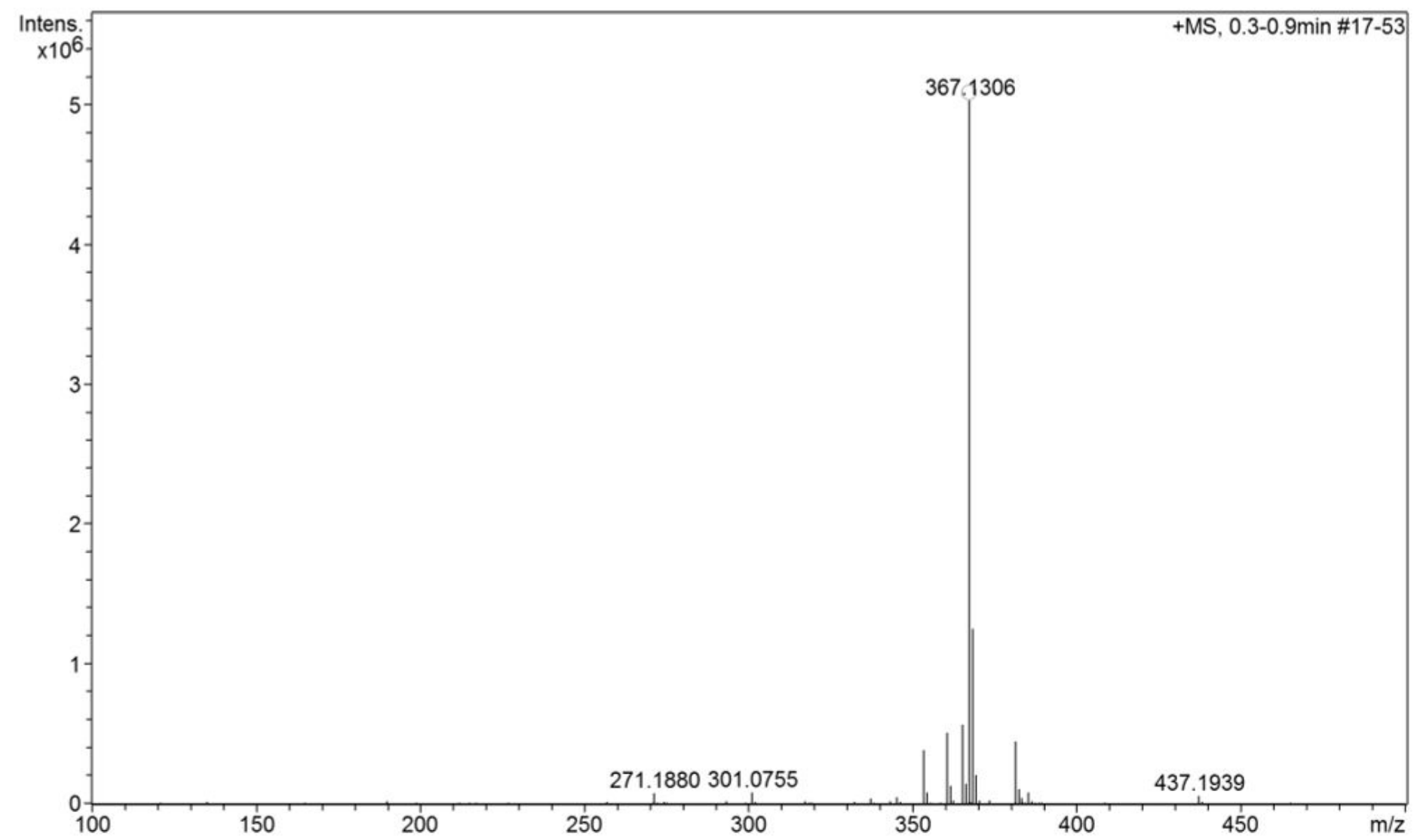

Meas. m/z	#	Ion Formula	m/z	err [ppm]	Mean err [ppm]	rdb	N-Rule	e⁻ Conf	mSigma	Std I	Std Mean m/z	Std I VarNorm	Std m/z Diff	Std Comb Dev
367.130639	1	C23H20NaO3	367.130465	-0.5	-1.8	13.5	ok	even	2.8	4.0	n.a.	n.a.	n.a.	n.a.

252995 - ESY062.d
Bruker Compass DataAnalysis 4.2 printed: 7/22/2025 10:26:46 AM by: Vinicius Nery Page 1 of 1

Supplementary File

Figure S16. High-resolution ESI-Qq-TOF mass spectrum of **3i**

Compound Spectrum SmartFormula Report

Analysis Info

Analysis Name	D:\Data\2025\IOC\CARLOS ALVES\252997 - ESY064.d	Acquisition Date	7/22/2025 10:29:30 AM
Method	Tune_pos_Standard.m	Operator	Vinicius Nery
Sample Name	ESY064	Instrument	compact 8255754.10035
Comment			

Acquisition Parameter

Source Type	ESI	Ion Polarity	Positive	Set Nebulizer	0.4 Bar
Focus	Not active	Set Capillary	4000 V	Set Dry Heater	200 °C
Scan Begin	100 m/z	Set End Plate Offset	-400 V	Set Dry Gas	4.0 l/min
Scan End	1000 m/z	Set Charging Voltage	2000 V	Set Divert Valve	Source
		Set Corona	0 nA	Set APCI Heater	0 °C

+MS, 0.3-1.0min #20-56

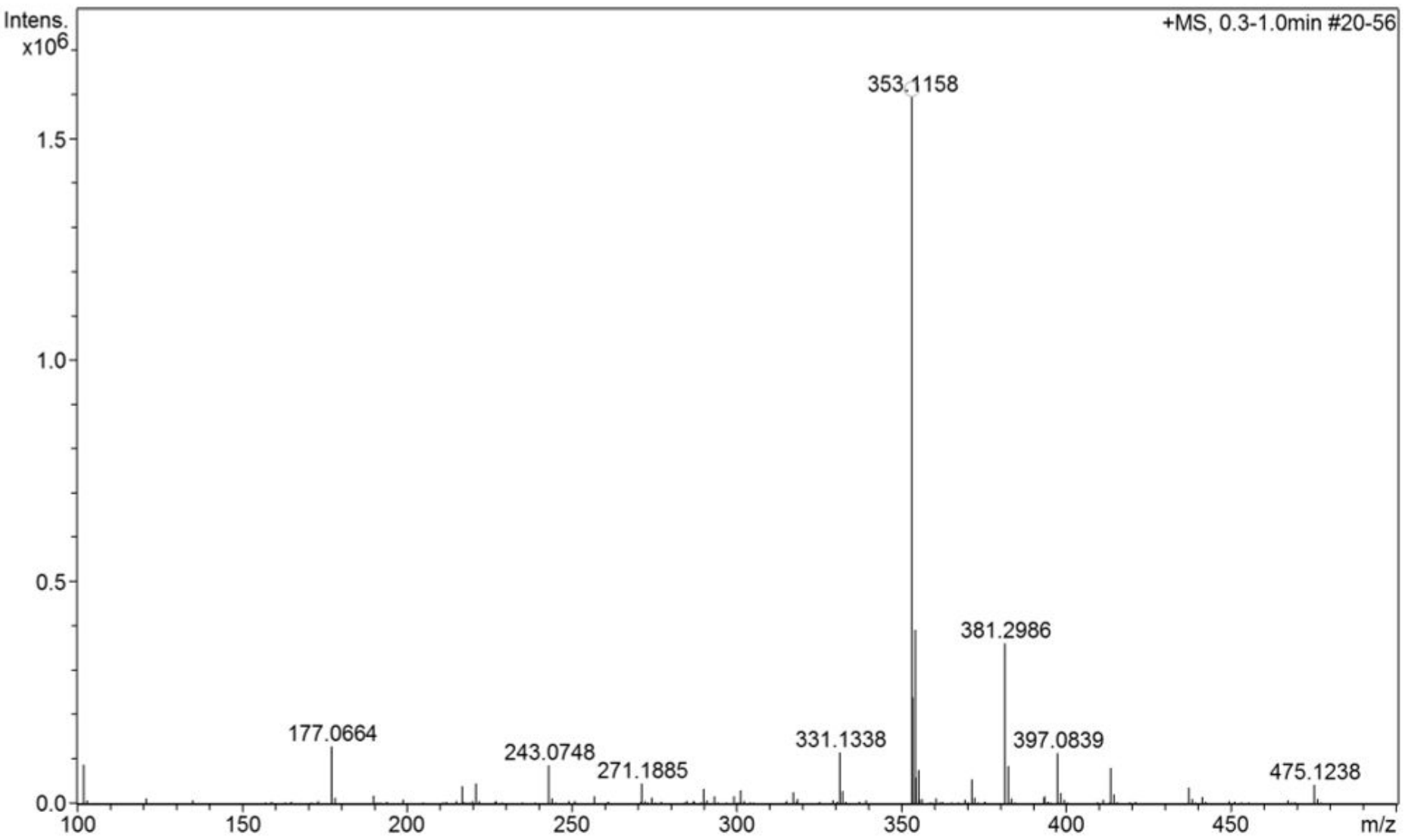

Meas. m/z	#	Ion Formula	m/z	err [ppm]	Mean err [ppm]	rdb	N-Rule	e⁻ Conf	mSigma	Std I	Std Mean m/z	Std I VarNorm	Std m/z Diff	Std Comb Dev
353.115752	1	C22H18NaO3	353.114815	-2.7	-4.5	13.5	ok	even	8.6	15.6	n.a.	n.a.	n.a.	n.a.

252997 - ESY064.d
Bruker Compass DataAnalysis 4.2 printed: 7/22/2025 10:32:23 AM by: Vinicius Nery Page 1 of 1

Figure S17. High-resolution ESI-Qq-TOF mass spectrum of **3j**

Compound Spectrum SmartFormula Report

Analysis Info

Analysis Name	D:\Data\2025\IOC\CARLOS ALVES\252998 - ESY065.d	Acquisition Date	7/22/2025 10:34:35 AM
Method	Tune_pos_Standard.m	Operator	Vinicius Nery
Sample Name	ESY065	Instrument	compact 8255754.10035
Comment			

Acquisition Parameter

Source Type	ESI	Ion Polarity	Positive	Set Nebulizer	0.4 Bar
Focus	Not active	Set Capillary	4000 V	Set Dry Heater	200 °C
Scan Begin	100 m/z	Set End Plate Offset	-400 V	Set Dry Gas	4.0 l/min
Scan End	1000 m/z	Set Charging Voltage	2000 V	Set Divert Valve	Source
		Set Corona	0 nA	Set APCI Heater	0 °C

+MS, 0.1-1.0min #6-59

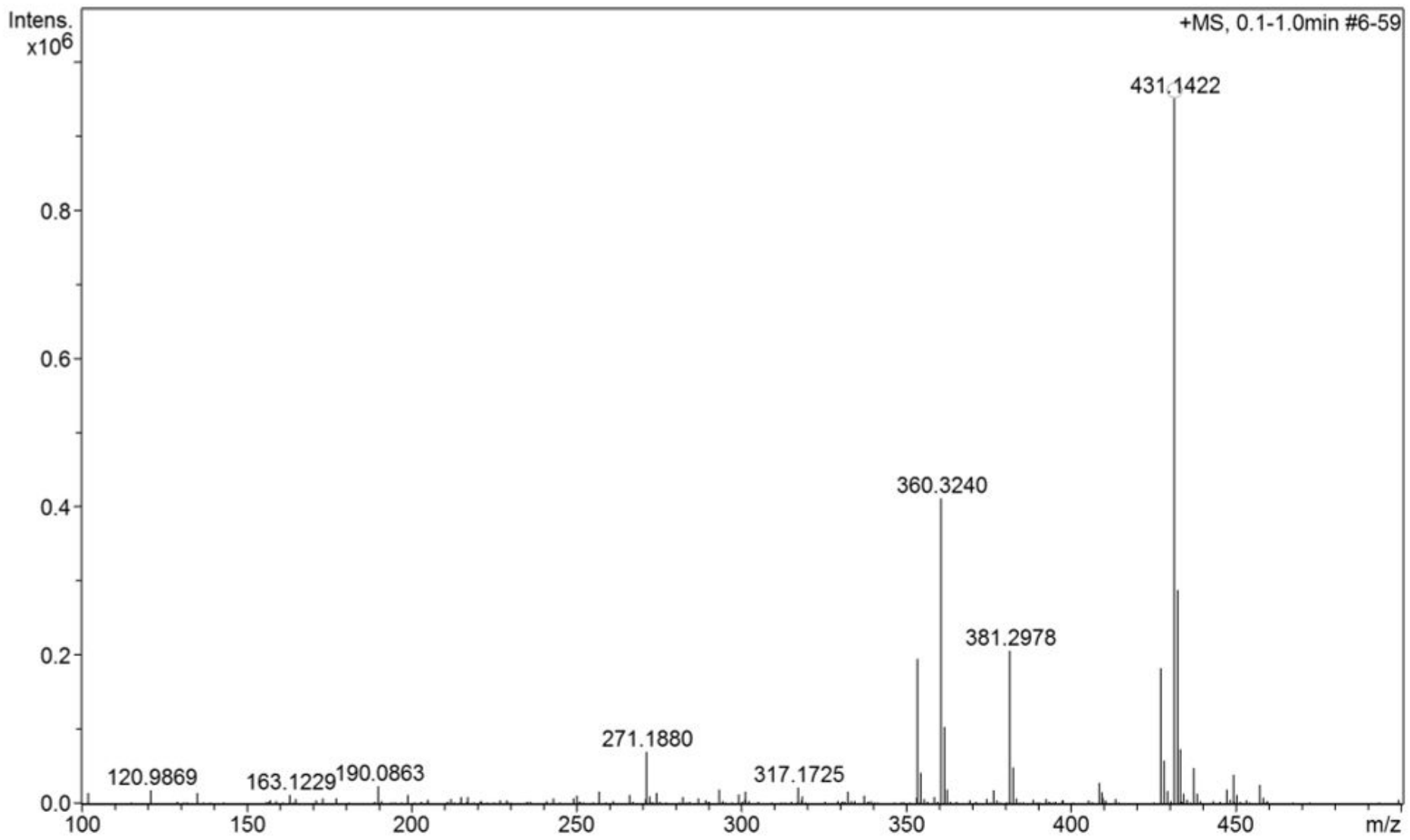

Meas. m/z	#	Ion Formula	m/z	err [ppm]	Mean err [ppm]	rdb	N-Rule	e¯ Conf	mSigma	Std I	Std Mean m/z	Std I VarNorm	Std m/z Diff	Std Comb Dev
431.142238	1	C28H21FNaO2	431.141779	-1.1	-2.9	17.5	ok	even	16.3	24.7	n.a.	n.a.	n.a.	n.a.

252998 - ESY065.d

Bruker Compass DataAnalysis 4.2 printed: 7/22/2025 10:36:58 AM by: Vinicius Nery Page 1 of 1

4. HPLC-UV and HPLC-DAD of Biological Evaluated Compounds

Figure S18. HPLC-UV chromatogram (254 nm) of **3c**

28/06/2024 16:07:21 Page 1 / 2

SHIMADZU LabSolutions **Analysis Report**

<Sample Information>

Sample Name	: ESY046_ISO		
Sample ID	: ESY046_ISO		
Data Filename	: ESY046_ISO tratado 254.lcd		
Method Filename	: ISO_88_ACN.lcm		
Batch Filename	: 06_07_23.lcb		
Vial #	: 1-7	Sample Type	: Unknown
Injection Volume	: 10 uL		
Date Acquired	: 21/08/2023 15:23:00	Acquired by	: System Administrator
Date Processed	: 28/06/2024 14:52:16	Processed by	: System Administrator

<Chromatogram>

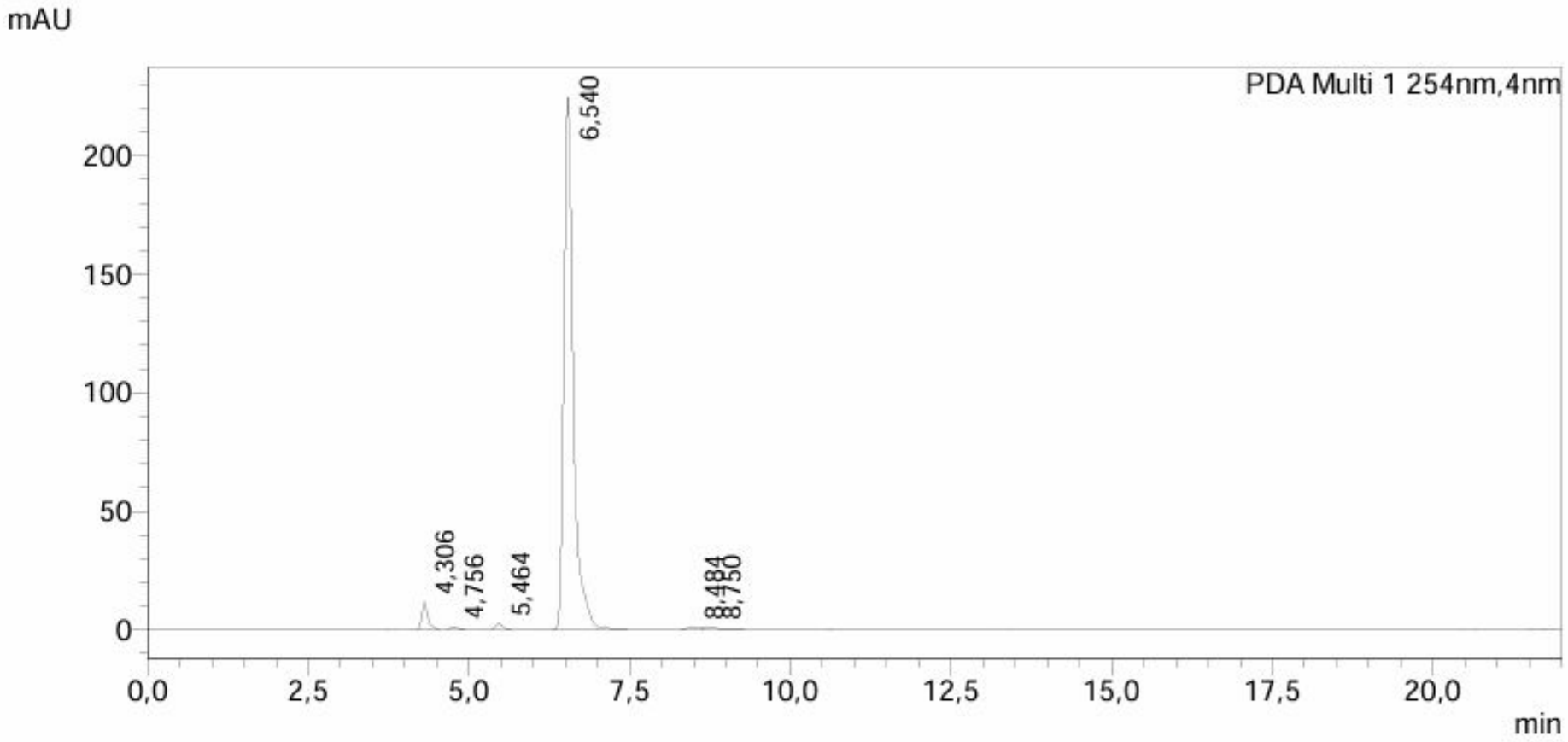

<Peak Table>
PDA Ch1 254nm

Peak#	Ret. Time	Area	Height	Area%
1	4,306	74329	11311	3,061
2	4,756	6814	1012	0,281
3	5,464	16459	2176	0,678
4	6,540	2306670	224408	94,983
5	8,484	11585	966	0,477
6	8,750	12657	949	0,521
Total		2428513	240822	100,000

Figure S19. HPLC-UV chromatogram (254 nm) of **3d**

29/06/2024 13:38:58 Page 1 / 2

SHIMADZU LabSolutions

Analysis Report

<Sample Information>

Sample Name	: ESY059_ISO		
Sample ID	: ESY059_ISO		
Data Filename	: ESY059_ISO tratado 254.lcd		
Method Filename	: ISO_88_ACN.lcm		
Batch Filename	: 06_07_23.lcb		
Vial #	: 1-15	Sample Type	: Unknown
Injection Volume	: 10 uL		
Date Acquired	: 21/08/2023 18:22:56	Acquired by	: System Administrator
Date Processed	: 28/06/2024 15:10:23	Processed by	: System Administrator

<Chromatogram>

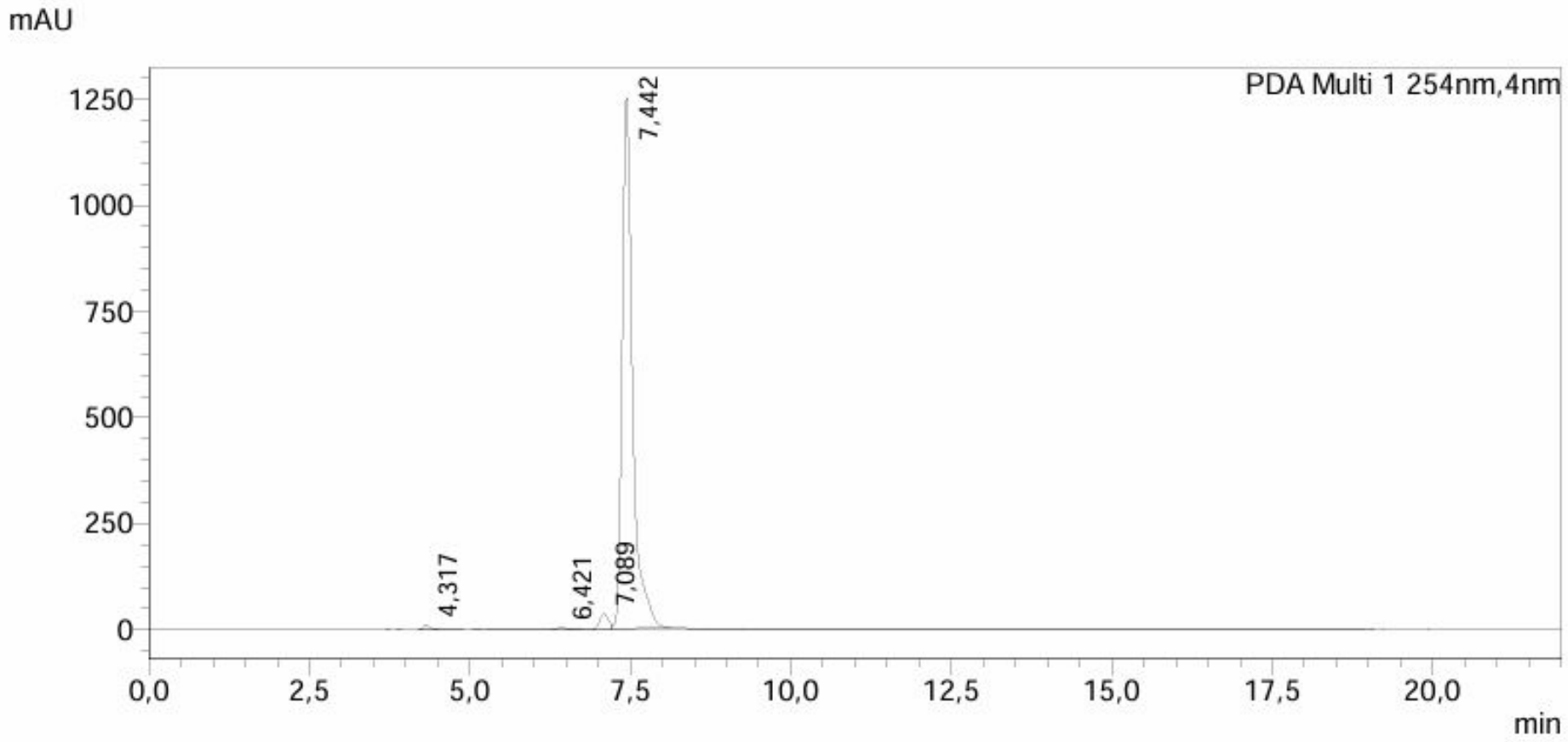

<Peak Table>

PDA Ch1 254nm

Peak#	Ret. Time	Area	Height	Area%
1	4,317	71231	10957	0,492
2	6,421	43251	4729	0,299
3	7,089	333664	35876	2,307
4	7,442	14017898	1250138	96,902
Total		14466043	1301699	100,000

Supplementary File

Figure S20. HPLC-UV chromatogram (254 nm) of **3e**

SHIMADZU LabSolutions

Analysis Report

<Sample Information>

Sample Name	: ESY061_ISO		
Sample ID	: ESY061_ISO		
Data Filename	: ESY061_ISO tratado 254.lcd		
Method Filename	: ISO_88_ACN.lcm		
Batch Filename	: 06_07_23.lcb		
Vial #	: 1-17	Sample Type	: Unknown
Injection Volume	: 10 uL		
Date Acquired	: 21/08/2023 19:07:53	Acquired by	: System Administrator
Date Processed	: 28/06/2024 15:22:25	Processed by	: System Administrator

<Chromatogram>

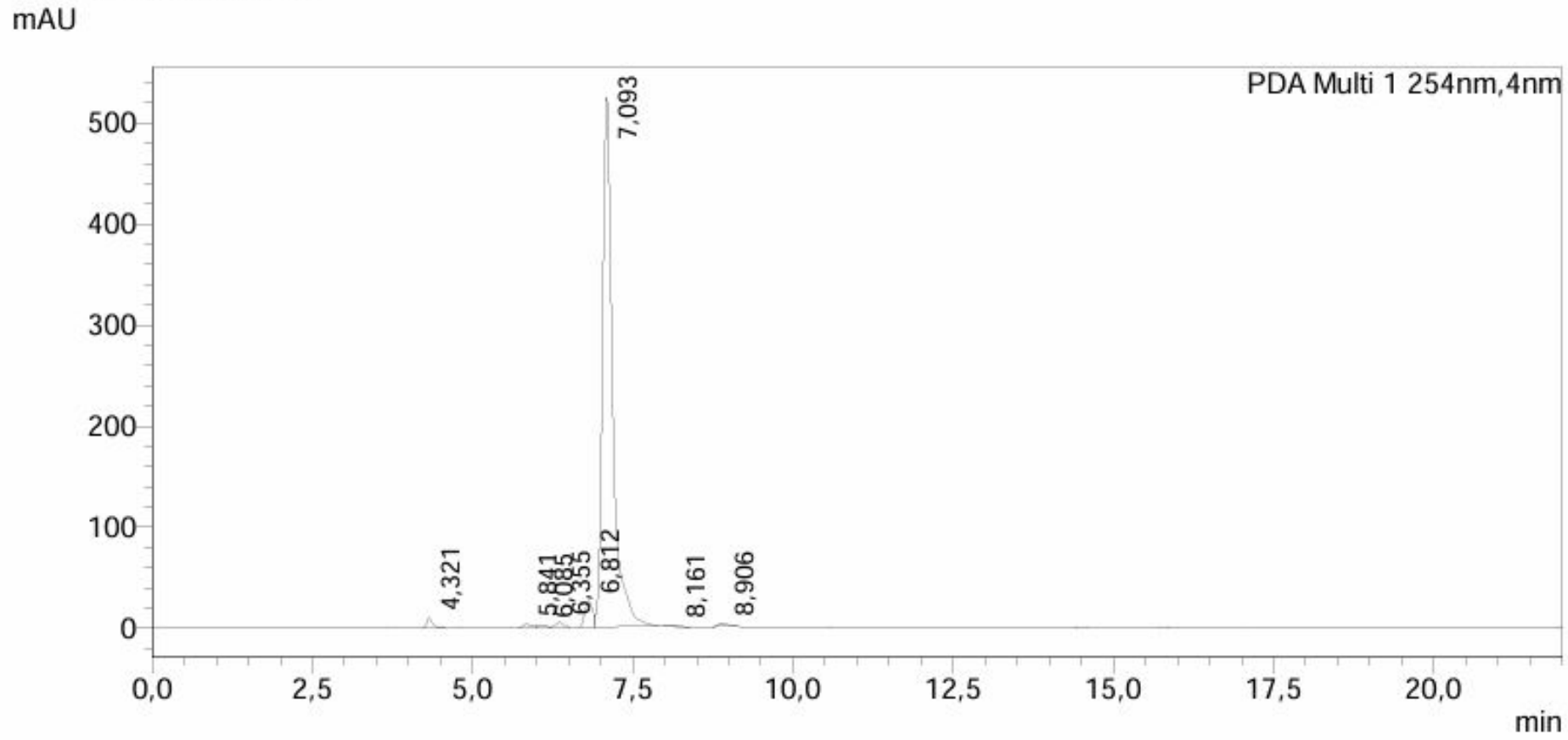

<Peak Table>
PDA Ch1 254nm

Peak#	Ret. Time	Area	Height	Area%
1	4,321	63532	9663	1,022
2	5,841	29236	3013	0,470
3	6,085	19484	2148	0,313
4	6,355	39432	4550	0,634
5	6,812	215442	25728	3,465
6	7,093	5799067	524410	93,276
7	8,161	13725	1296	0,221
8	8,906	37205	3376	0,598
Total		6217123	574184	100,000

Supplementary File

Figure S21. HPLC-UV chromatogram (254 nm) of **3f**

29/06/2024 13:41:21 Page 1 / 2

SHIMADZU LabSolutions **Analysis Report**

<Sample Information>

Sample Name : ESY060_ISO
Sample ID : ESY060_ISO
Data Filename : ESY060_ISO tratado 254.lcd
Method Filename : ISO_88_ACN.lcm
Batch Filename : 06_07_23.lcb
Vial # : 1-16 Sample Type : Unknown
Injection Volume : 10 uL
Date Acquired : 21/08/2023 18:45:25 Acquired by : System Administrator
Date Processed : 28/06/2024 15:13:16 Processed by : System Administrator

<Chromatogram>

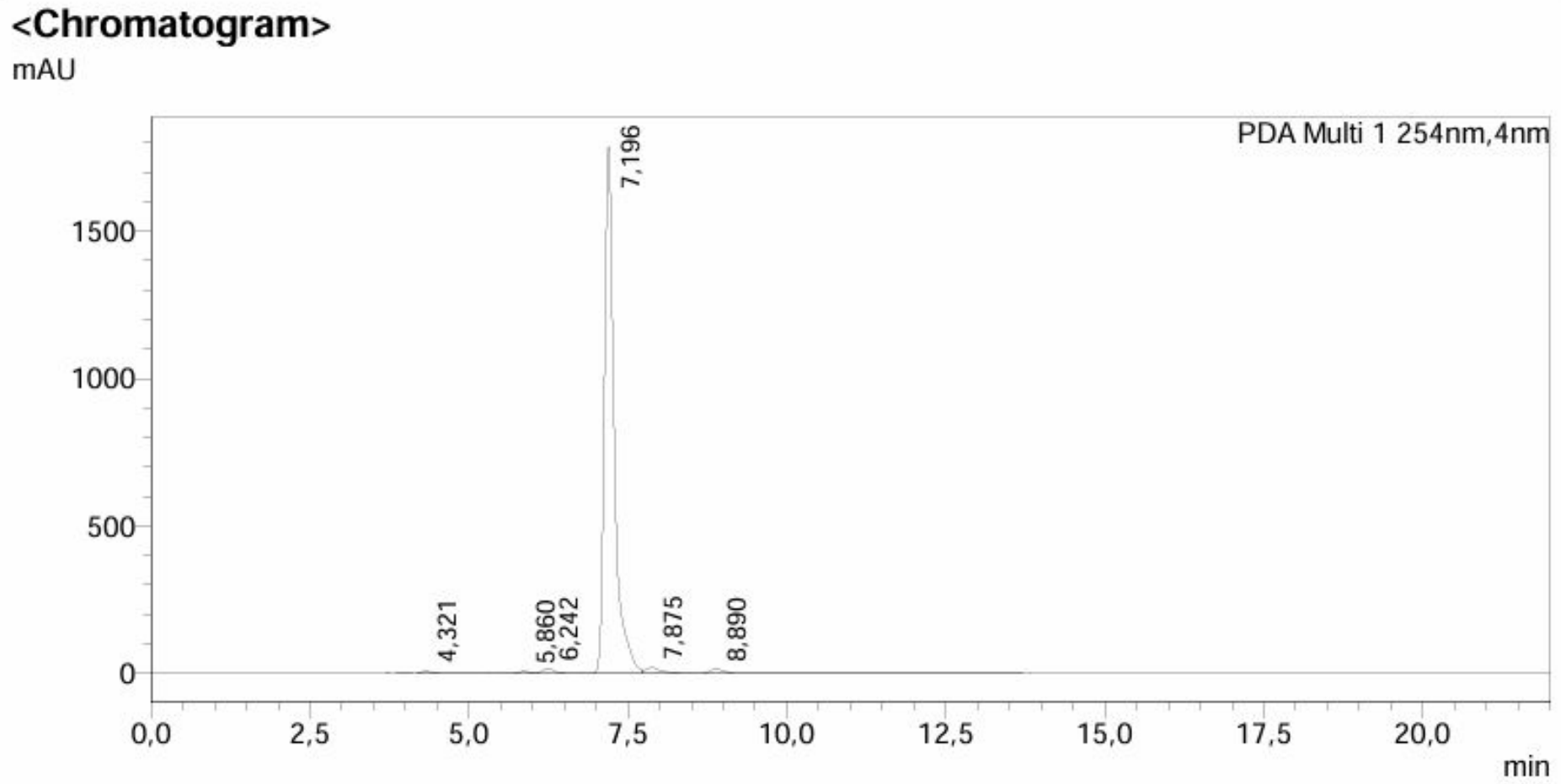

<Peak Table>
PDA Ch1 254nm

Peak#	Ret. Time	Area	Height	Area%
1	4,321	72006	11266	0,361
2	5,860	48119	4865	0,242
3	6,242	168229	16213	0,844
4	7,196	19243737	1783688	96,595
5	7,875	258361	17692	1,297
6	8,890	131580	11222	0,660
Total		19922032	1844946	100,000

Figure S22. HPLC-UV chromatogram (254 nm) of **3g**

29/06/2024 13:27:26 Page 1 / 2

SHIMADZU LabSolutions **Analysis Report**

<Sample Information>

Sample Name	: ESY051_ISO		
Sample ID	: ESY051_ISO		
Data Filename	: ESY051_ISO tratado 254.lcd		
Method Filename	: ISO_88_ACN.lcm		
Batch Filename	: 06_07_23.lcb		
Vial #	: 1-12	Sample Type	: Unknown
Injection Volume	: 10 uL		
Date Acquired	: 21/08/2023 17:15:26	Acquired by	: System Administrator
Date Processed	: 28/06/2024 15:03:05	Processed by	: System Administrator

<Chromatogram>

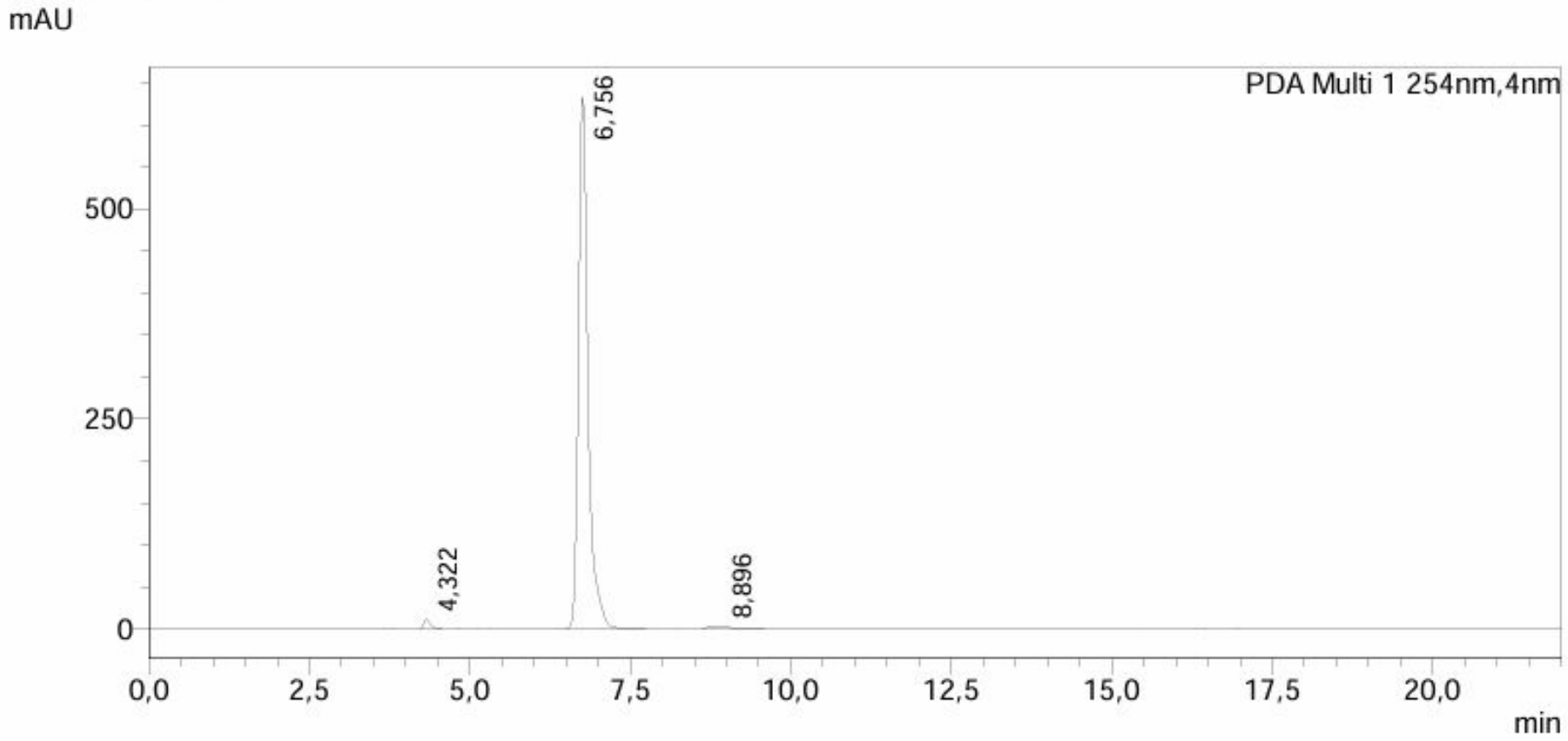

<Peak Table>
PDA Ch1 254nm

Peak#	Ret. Time	Area	Height	Area%
1	4,322	73543	11330	1,097
2	6,756	6562852	632079	97,879
3	8,896	68686	3252	1,024
Total		6705082	646661	100,000

Supplementary File

Figure S23. HPLC-UV chromatogram (254 nm) of **3h**

28/06/2024 15:29:54 Page 1 / 2

SHIMADZU LabSolutions **Analysis Report**

<Sample Information>

Sample Name	: ESY062_ISO		
Sample ID	: ESY062_ISO		
Data Filename	: ESY062_ISO.lcd		
Method Filename	: ISO_88_ACN.lcm		
Batch Filename	: 06_07_23.lcb		
Vial #	: 1-18	Sample Type	: Unknown
Injection Volume	: 10 uL		
Date Acquired	: 21/08/2023 19:30:22	Acquired by	: System Administrator
Date Processed	: 30/08/2023 14:54:59	Processed by	: System Administrator

<Chromatogram>

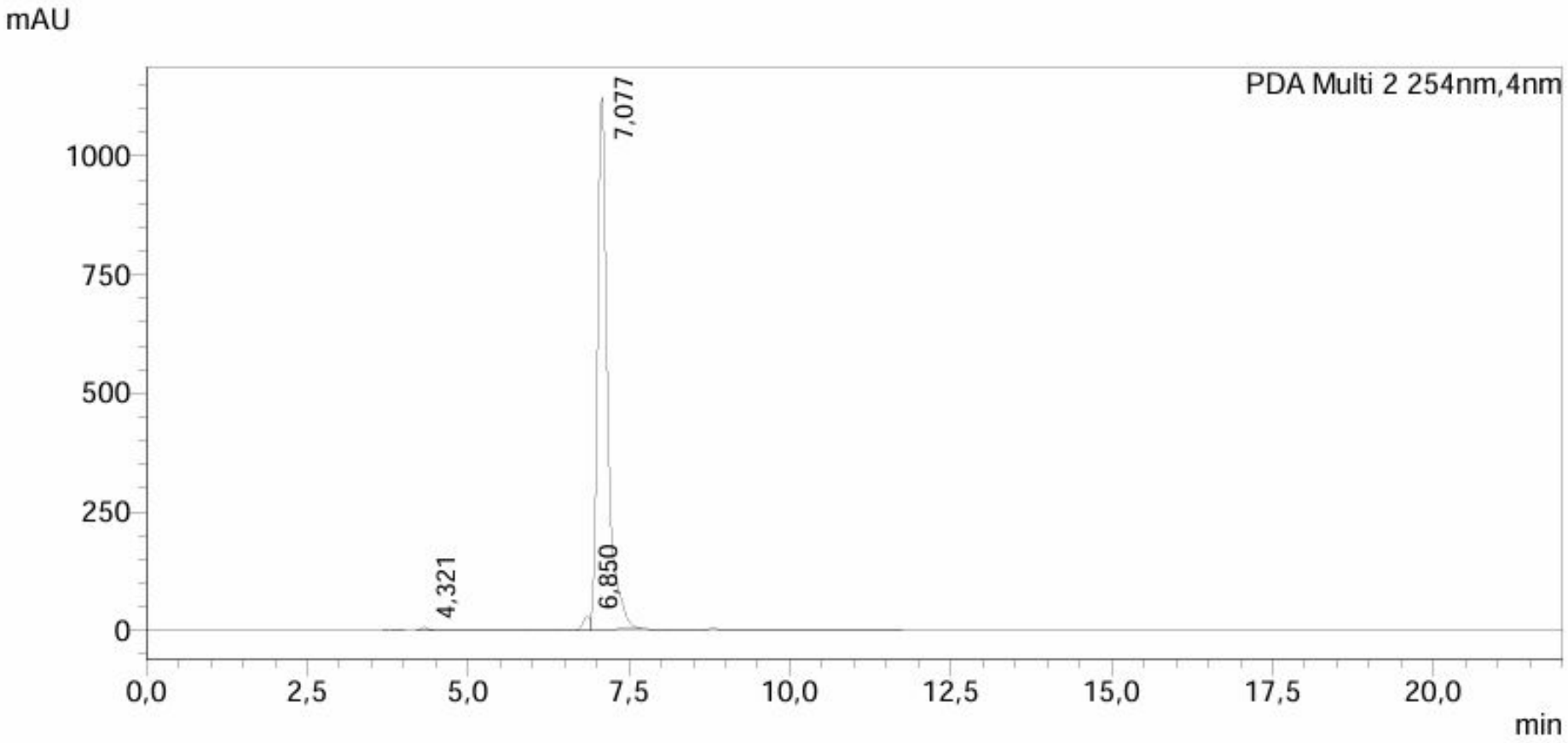

<Peak Table>

PDA Ch1 254nm

Peak#	Ret. Time	Area	Height	Area%
1	4,321	41421	7005	0,335
2	6,850	187984	27076	1,519
3	7,077	12144177	1121152	98,146
Total		12373582	1155233	100,000

Supplementary File

Figure S24. HPLC-DAD chromatogram (254 nm) of **3i**

28/06/2024 15:32:57 Page 1 / 2

SHIMADZU LabSolutions **Analysis Report**

<Sample Information>

Sample Name	: ESY064_ISO		
Sample ID	: ESY064_ISO		
Data Filename	: ESY064_ISO.lcd		
Method Filename	: ISO_88_ACN.lcm		
Batch Filename	: 06_07_23.lcb		
Vial #	: 1-19	Sample Type	: Unknown
Injection Volume	: 10 uL		
Date Acquired	: 21/08/2023 19:52:51	Acquired by	: System Administrator
Date Processed	: 30/08/2023 14:54:24	Processed by	: System Administrator

<Chromatogram>

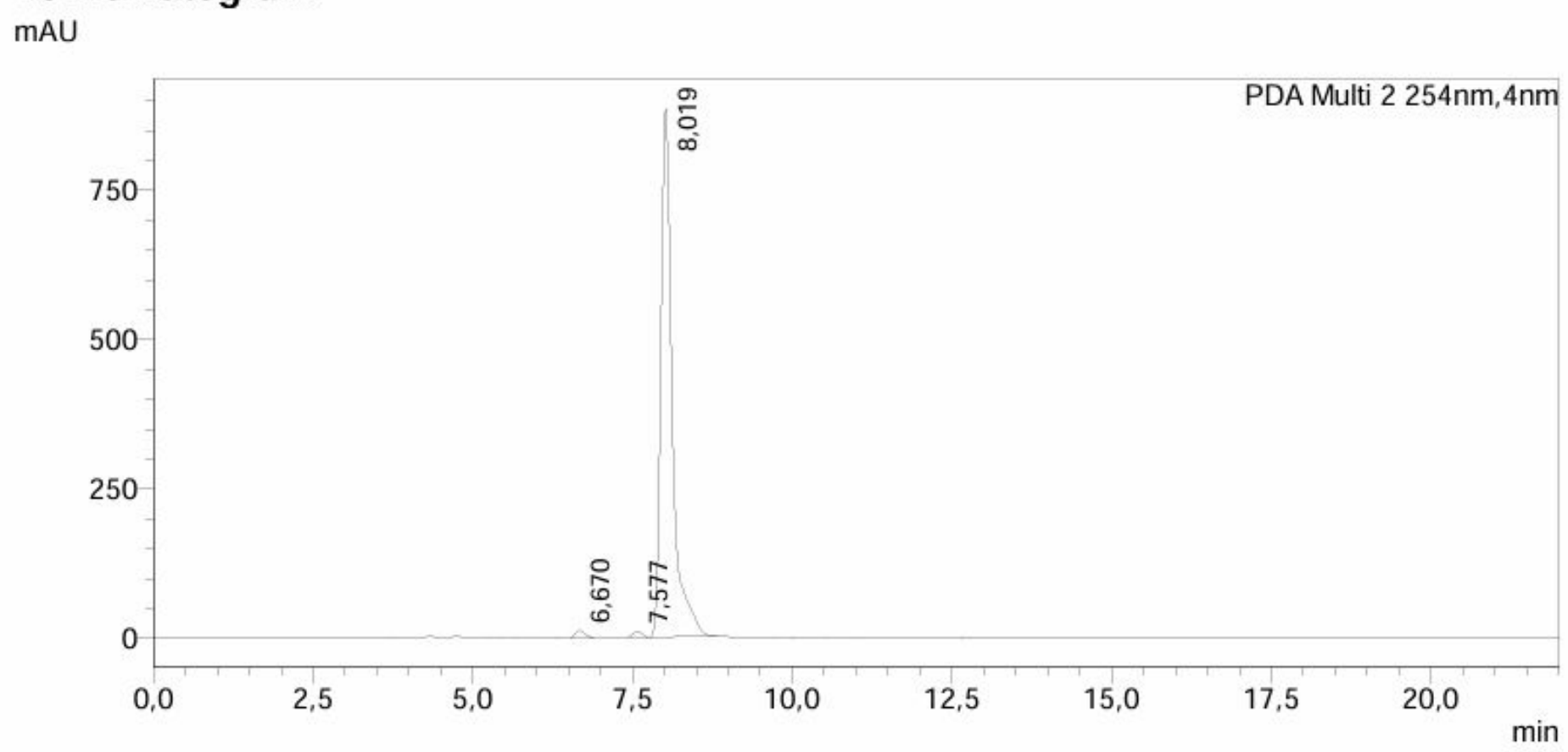

<Peak Table>
PDA Ch1 254nm

Peak#	Ret. Time	Area	Height	Area%
1	6,670	112147	11370	1,007
2	7,577	85134	9422	0,765
3	8,019	10936379	883743	98,228
Total		11133660	904536	100,000

Supplementary File

Figure S25. HPLC-DAD chromatogram (254 nm) of **3j**

29/06/2024 14:03:15 Page 1 / 2

SHIMADZU LabSolutions **Analysis Report**

<Sample Information>

Sample Name	: ESY065_ISO		
Sample ID	: ESY065_ISO		
Data Filename	: ESY065_ISO tratado 254.lcd		
Method Filename	: ISO_88_ACN.lcm		
Batch Filename	: 06_07_23.lcb		
Vial #	: 1-20	Sample Type	: Unknown
Injection Volume	: 10 uL		
Date Acquired	: 21/08/2023 20:15:19	Acquired by	: System Administrator
Date Processed	: 29/06/2024 13:45:44	Processed by	: System Administrator

<Chromatogram>

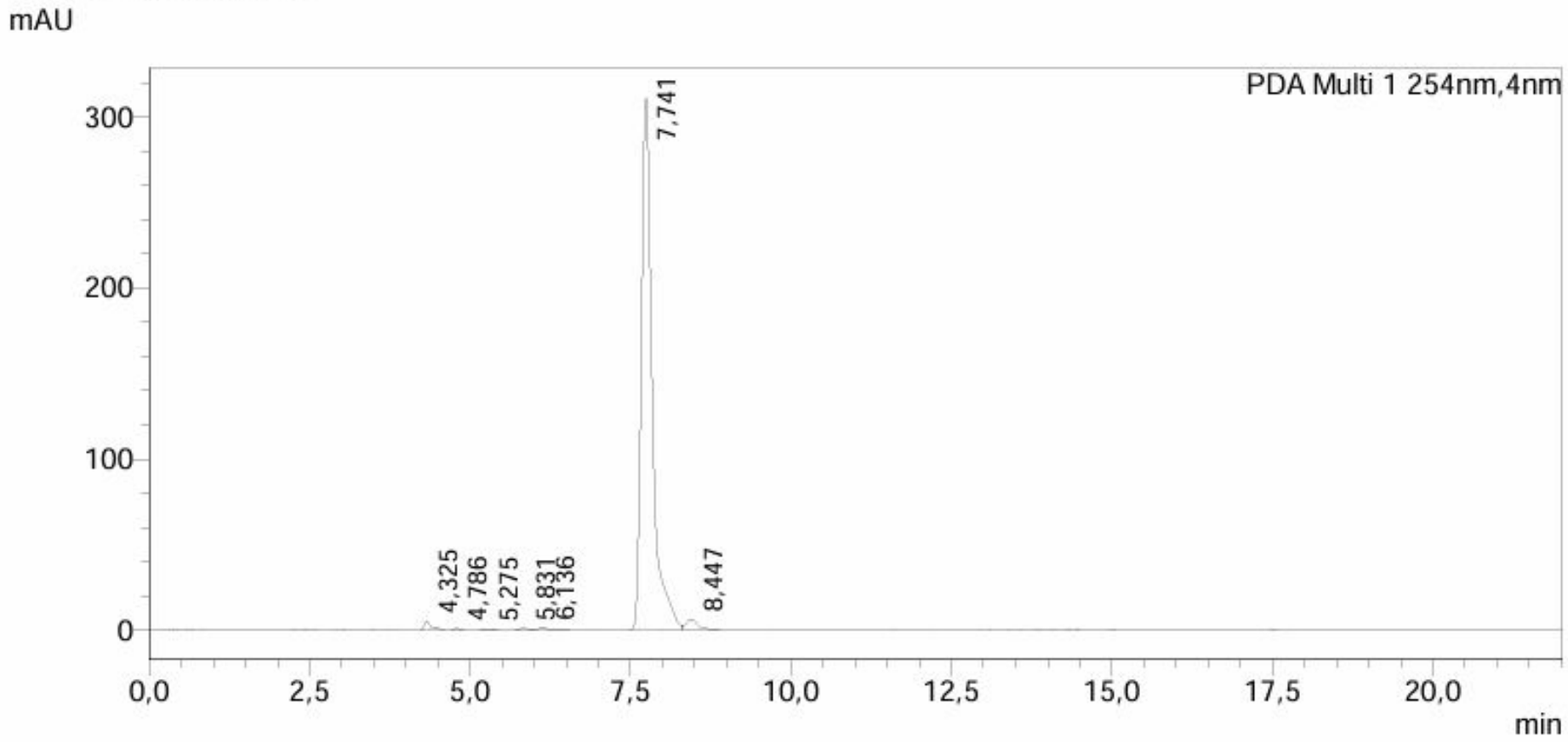

<Peak Table>
PDA Ch1 254nm

Peak#	Ret. Time	Area	Height	Area%
1	4,325	36176	4959	0,949
2	4,786	3613	641	0,095
3	5,275	3952	530	0,104
4	5,831	7720	945	0,202
5	6,136	14851	1275	0,389
6	7,741	3672035	310793	96,277
7	8,447	75674	6230	1,984
Total		3814021	325373	100,000

5. Nuclear Magnetic Resonance Spectra of Synthesized Compounds

Figure S26. ^{1}H and ^{13}C APT NMR spectra of compound **2a**

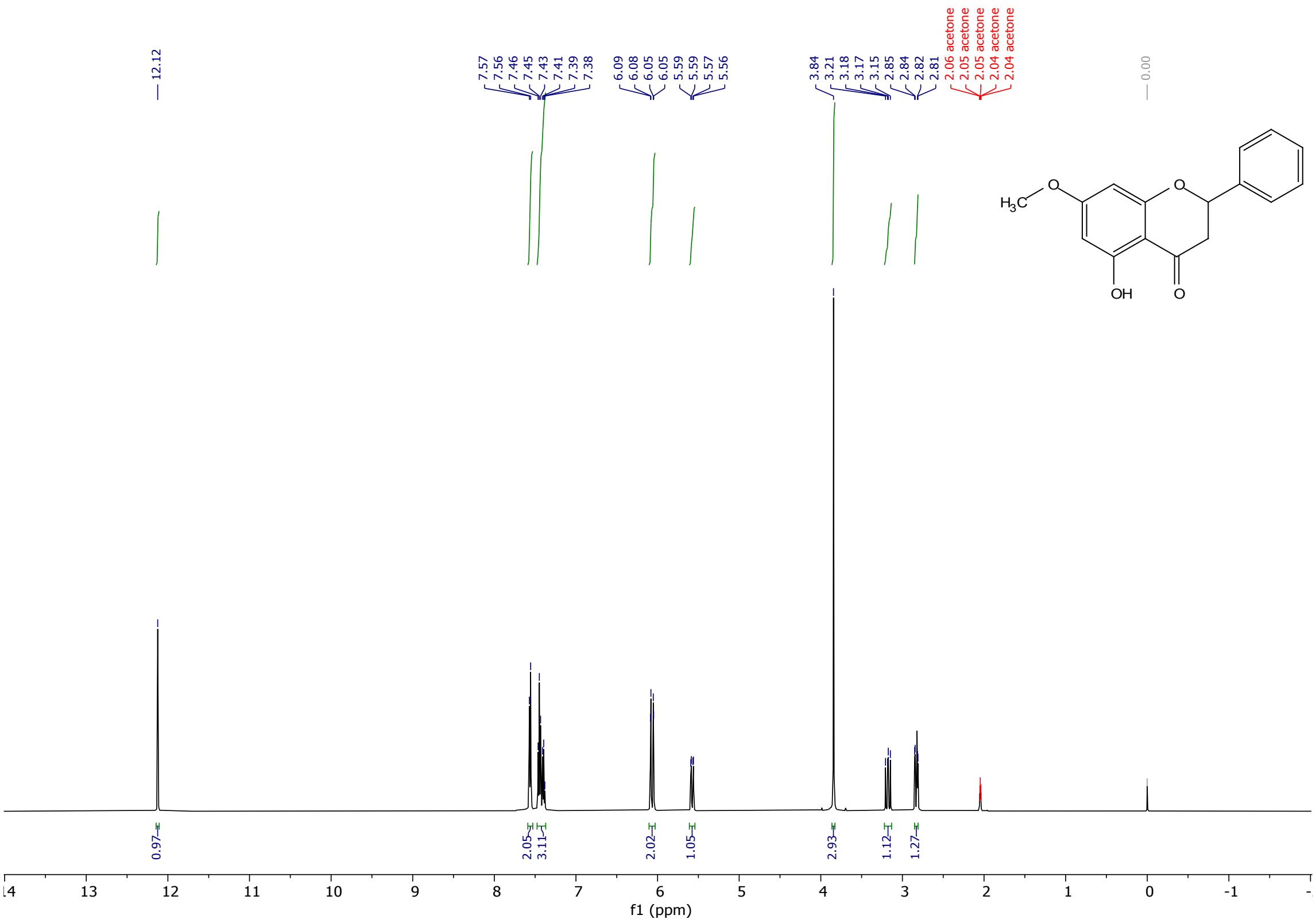

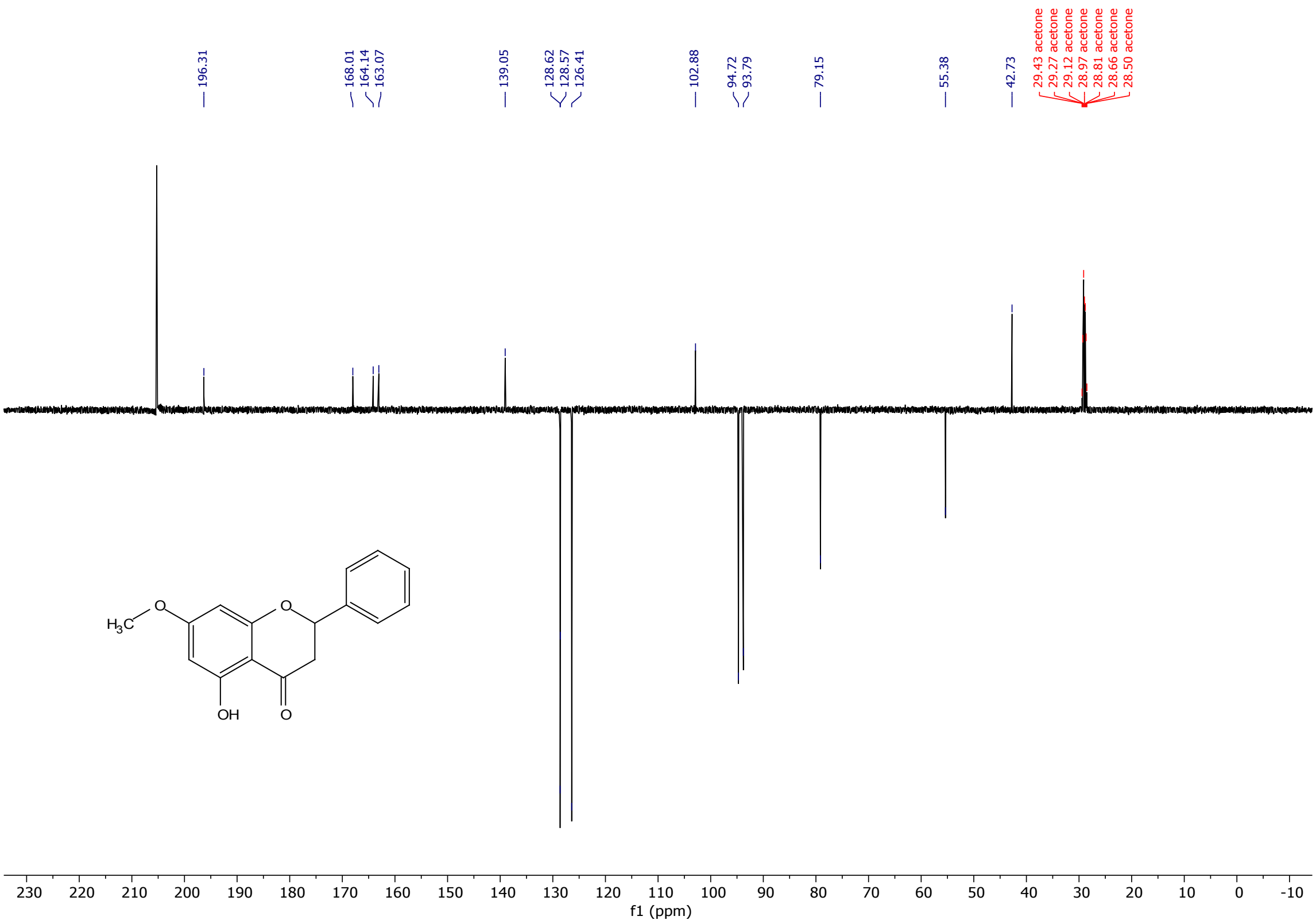

Figure S27. ^{1}H and ^{13}C APT NMR spectra of compound **2b**

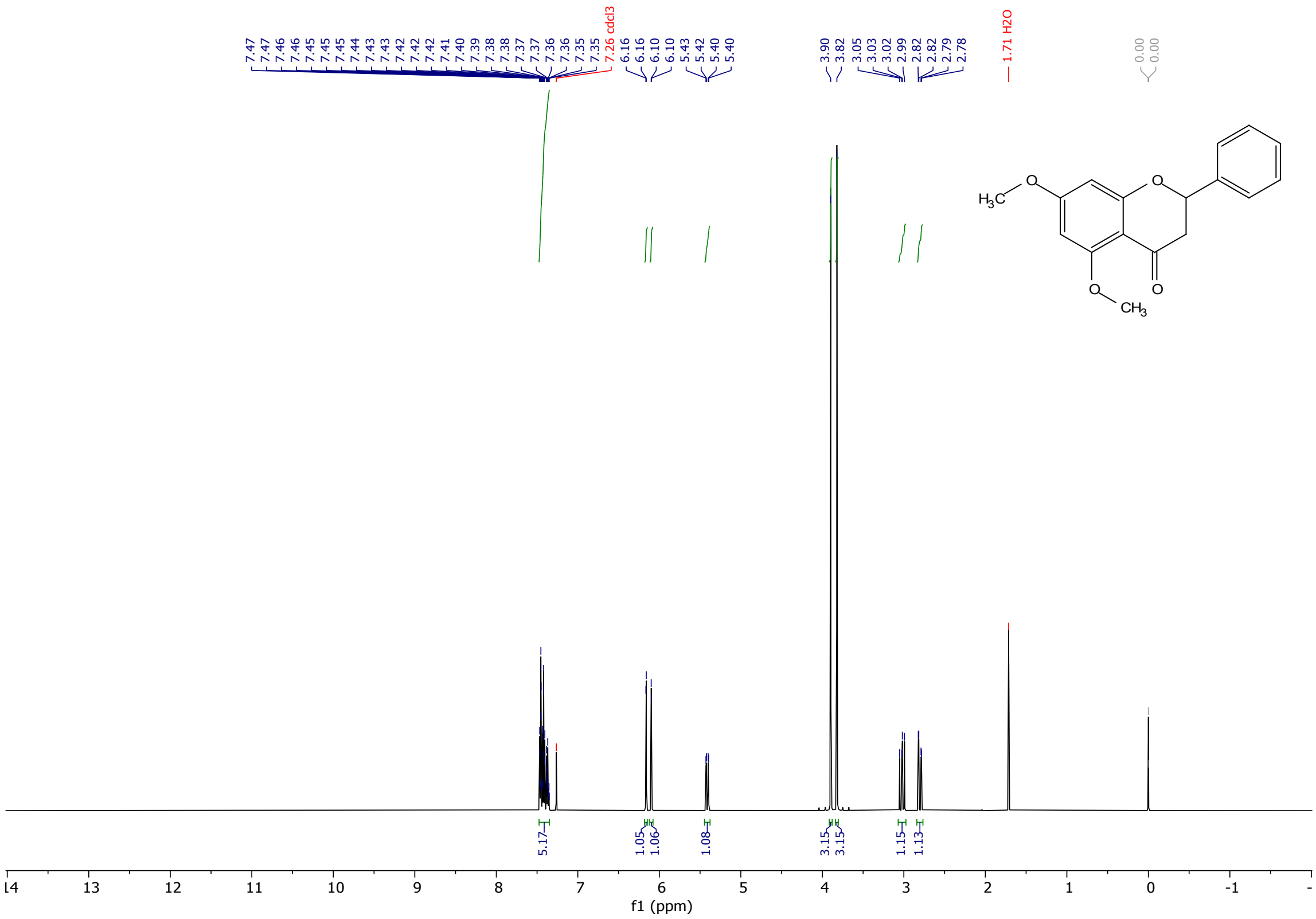

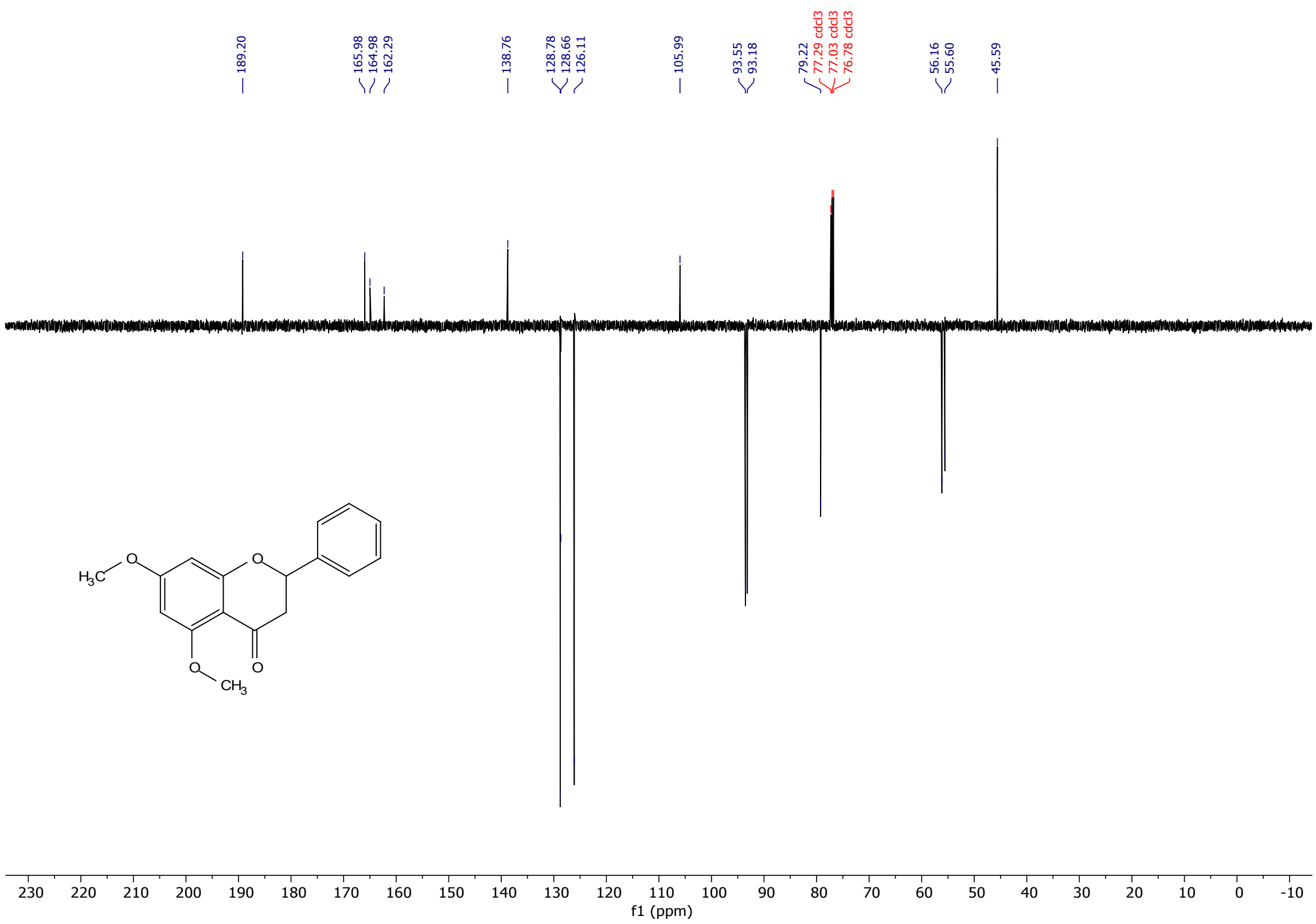

Figure S28. ^{1}H and ^{13}C APT NMR spectra of compound **2c**

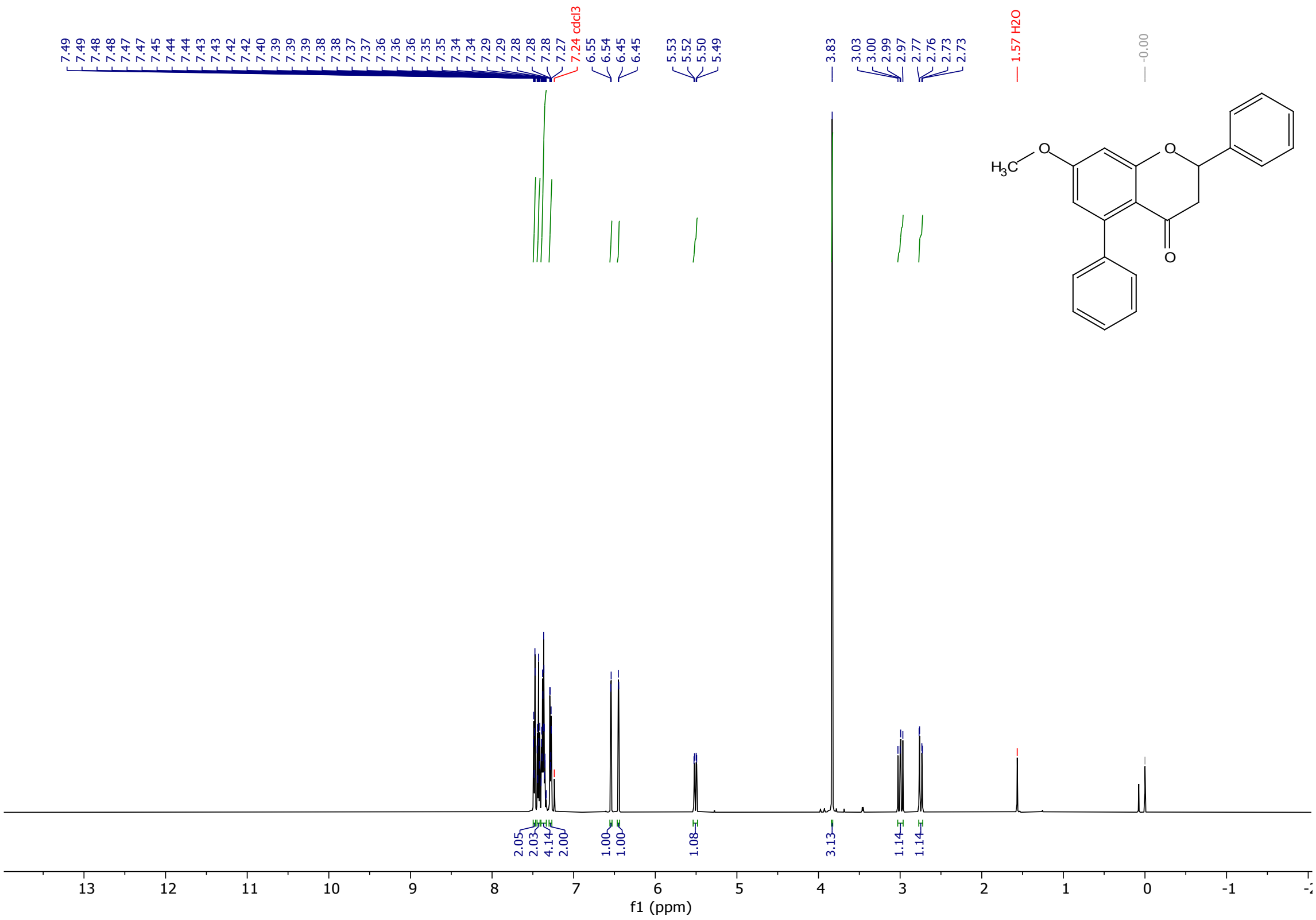

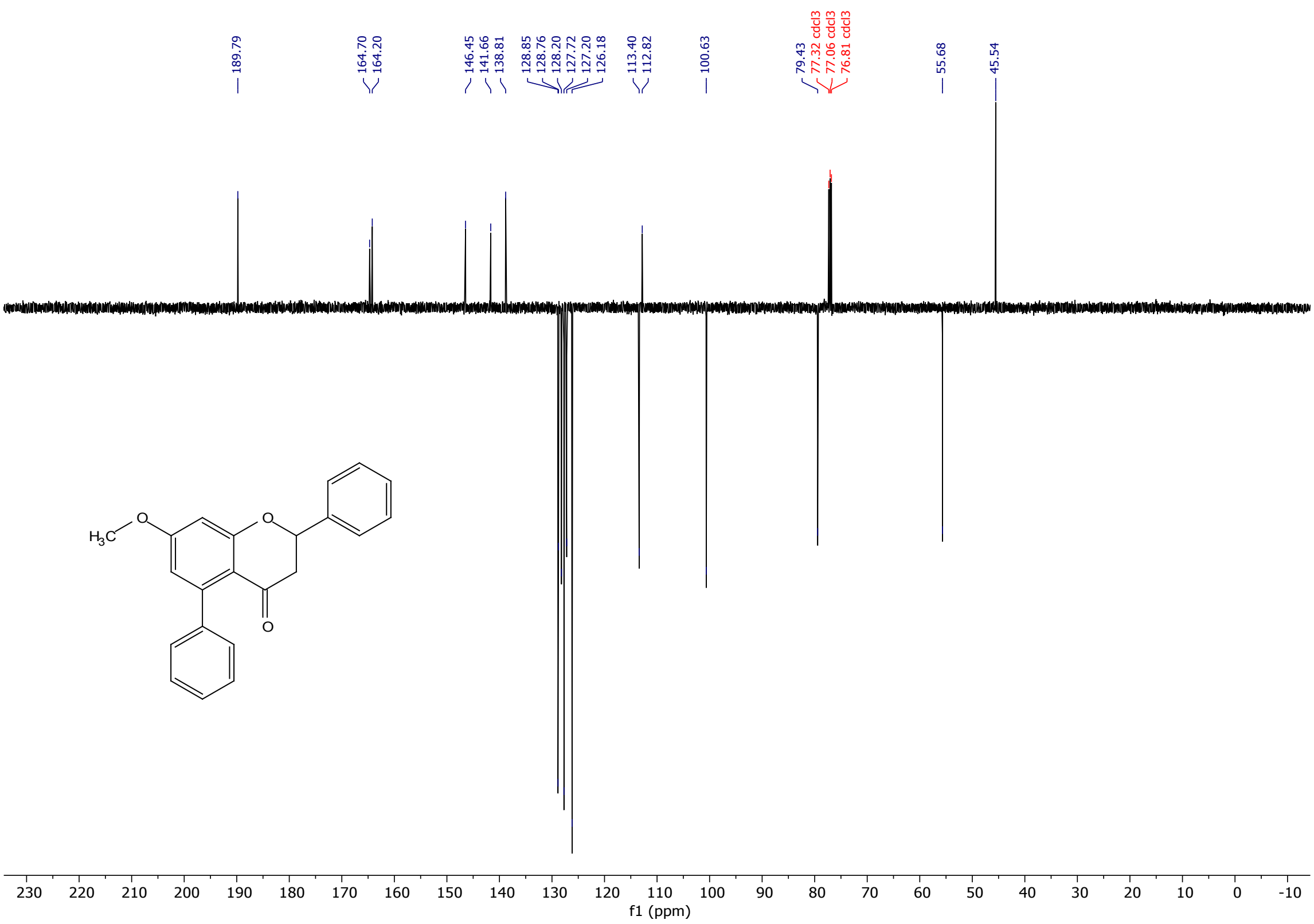

Figure S29. ^{1}H and ^{13}C APT NMR spectra of compound **2d**

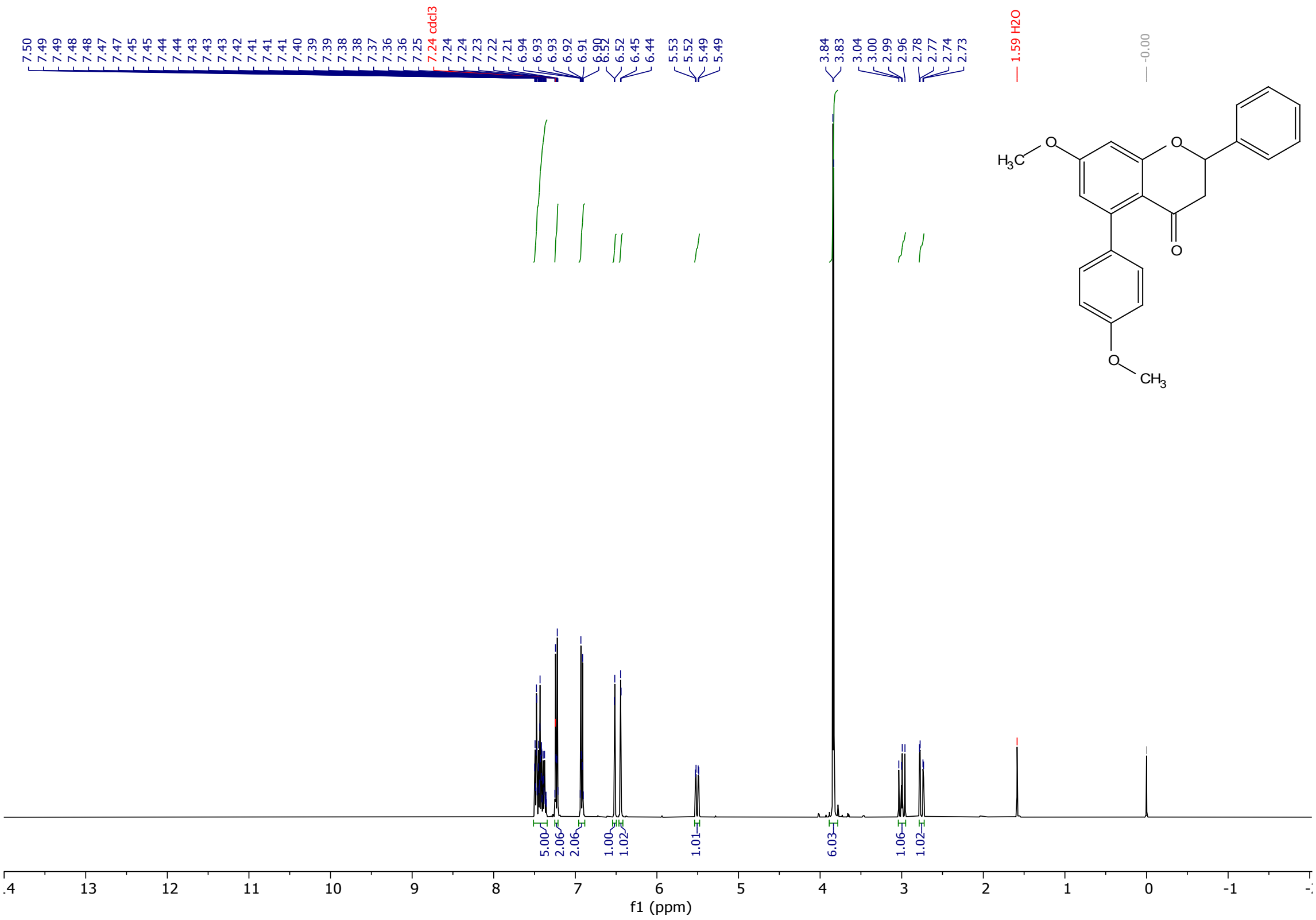

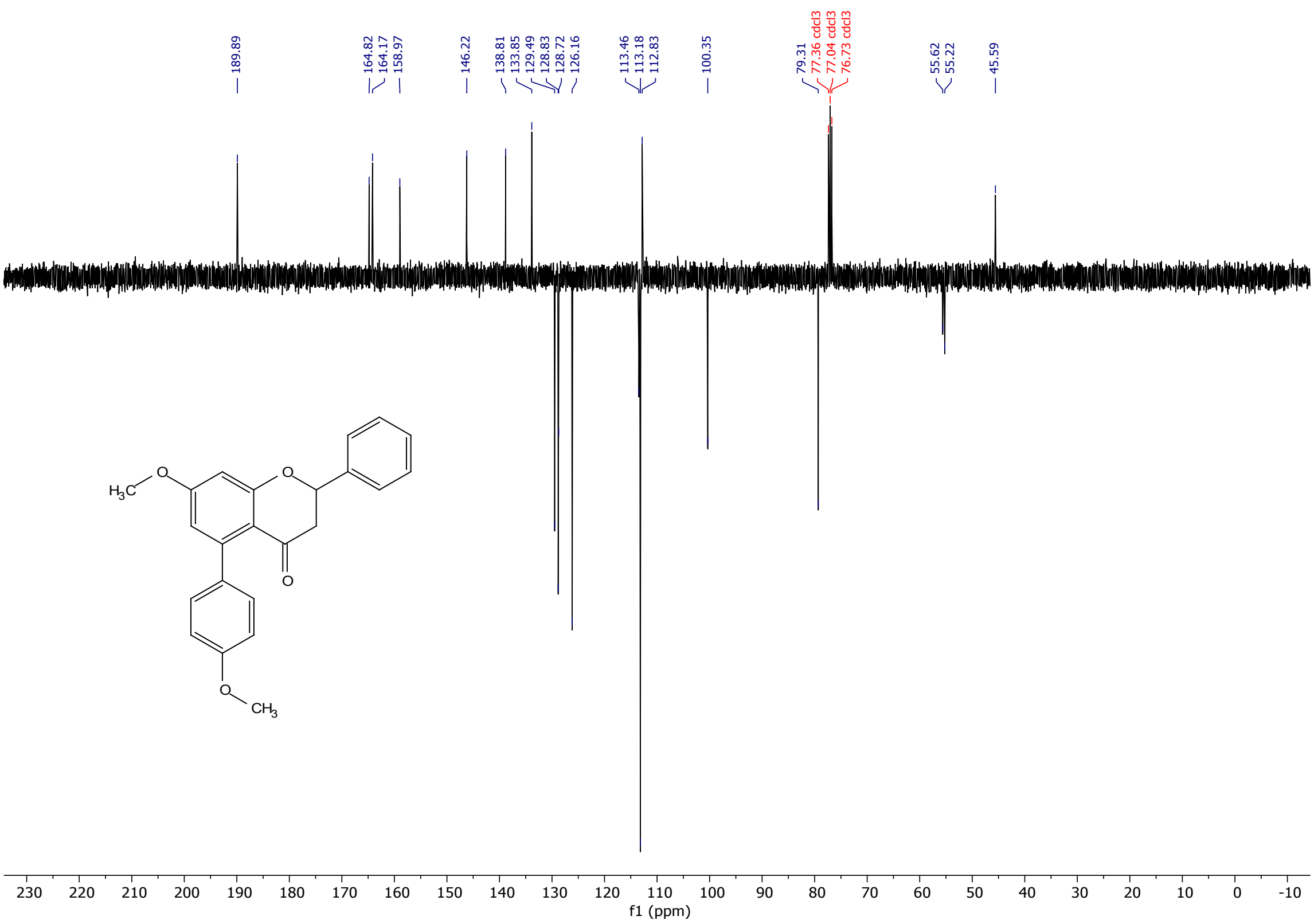

Figure S30. ^{1}H and ^{13}C APT NMR spectra of compound **2e**

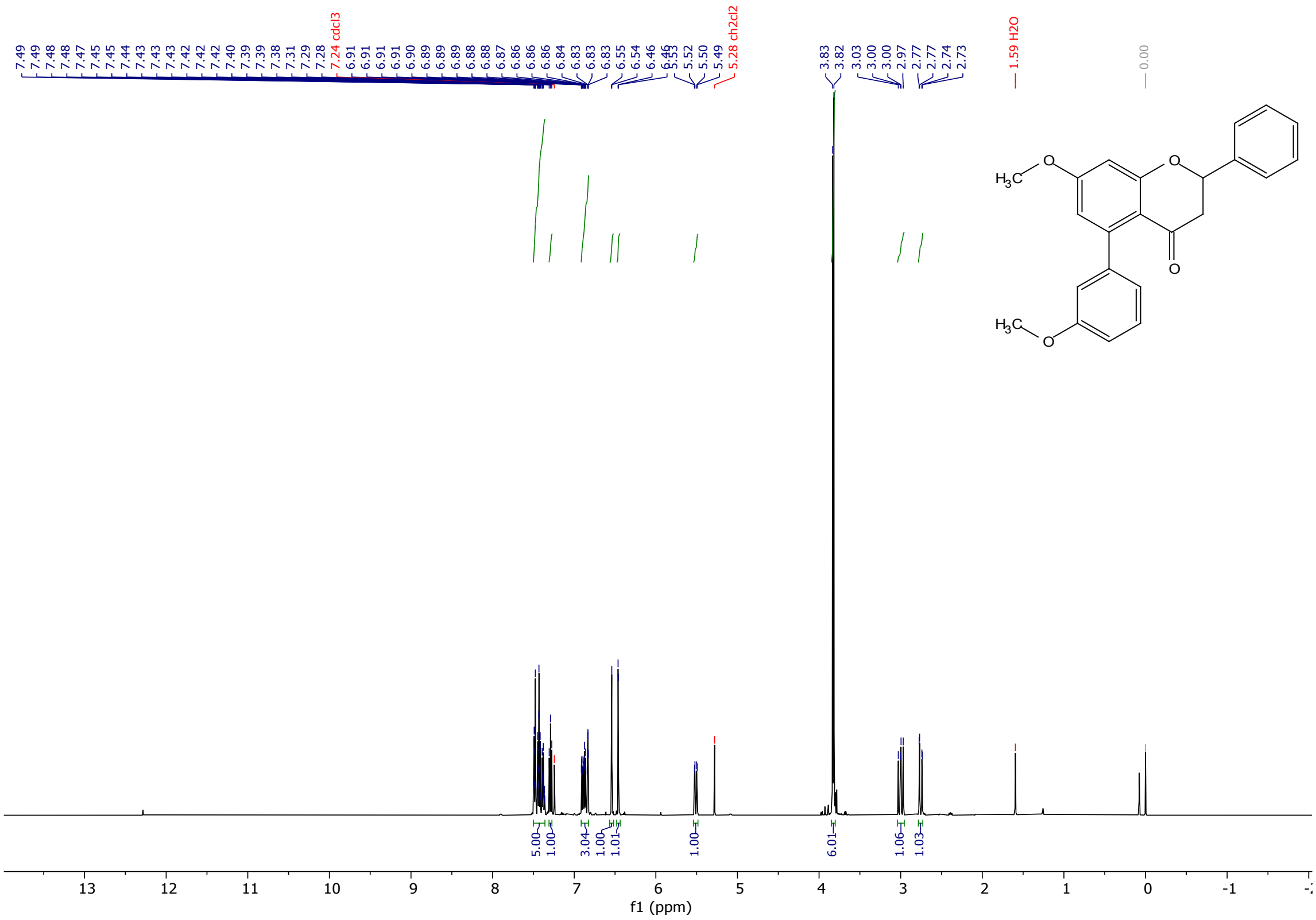

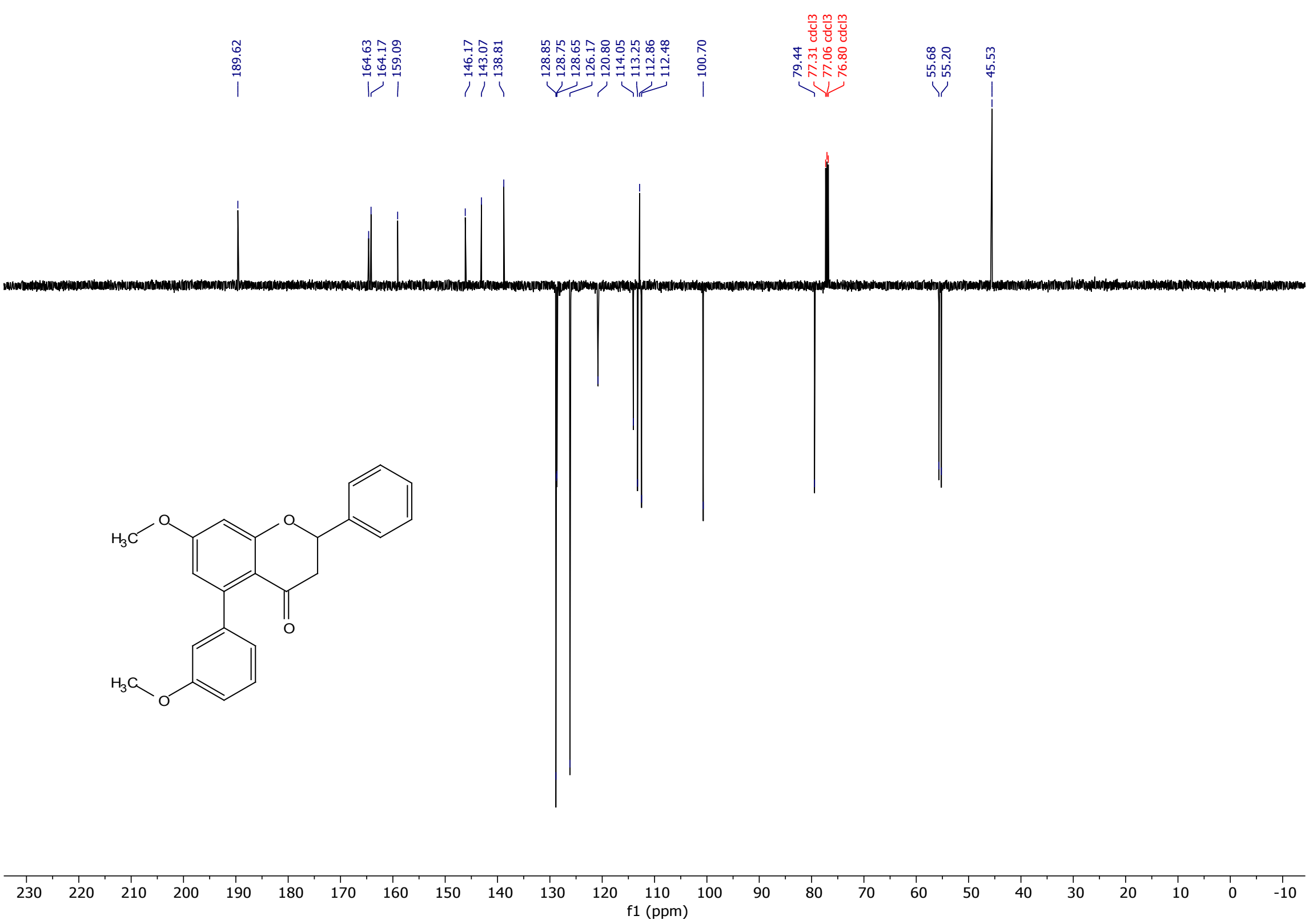

Figure S31. ^{1}H and ^{13}C APT NMR spectra of compound **2f**

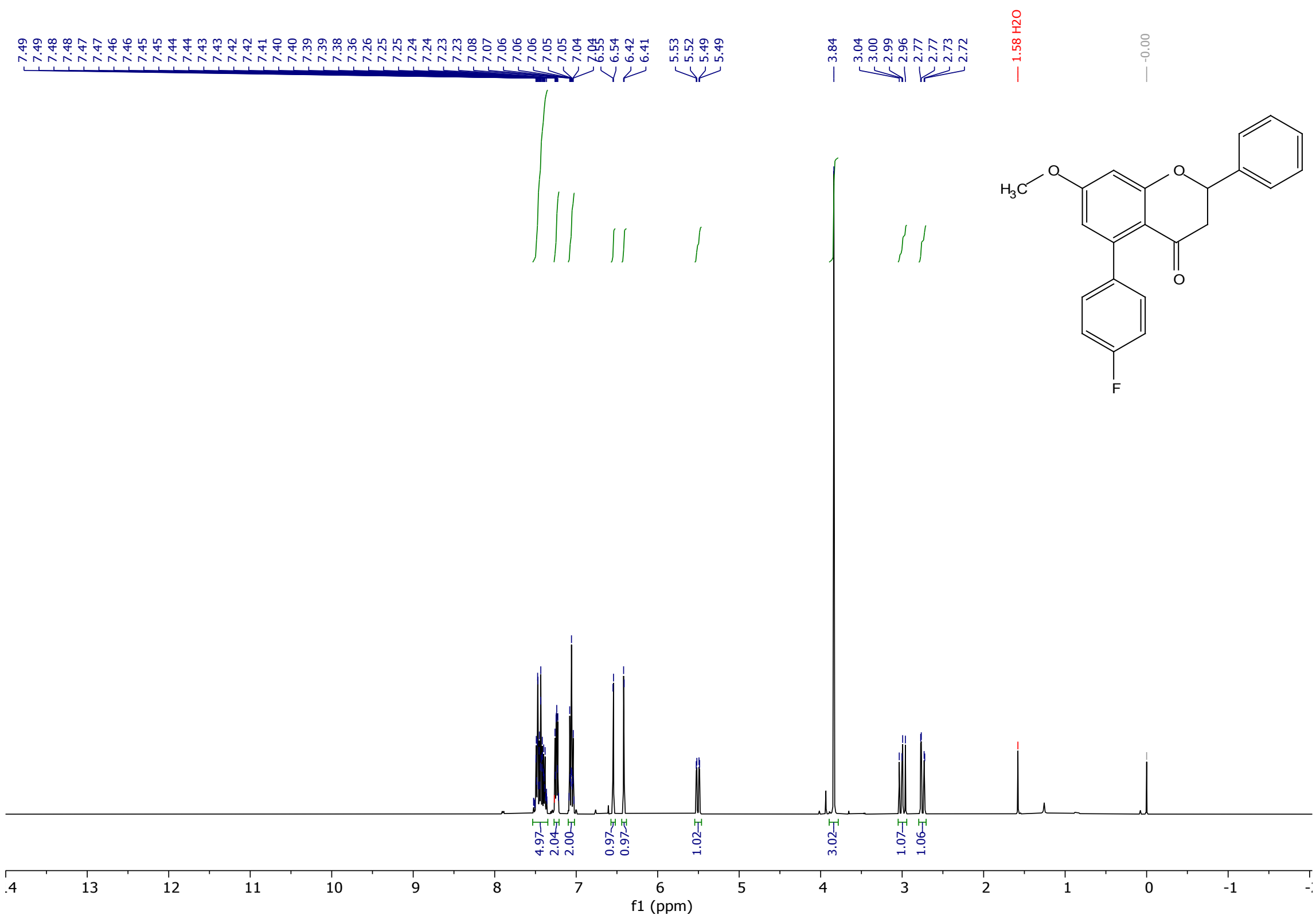

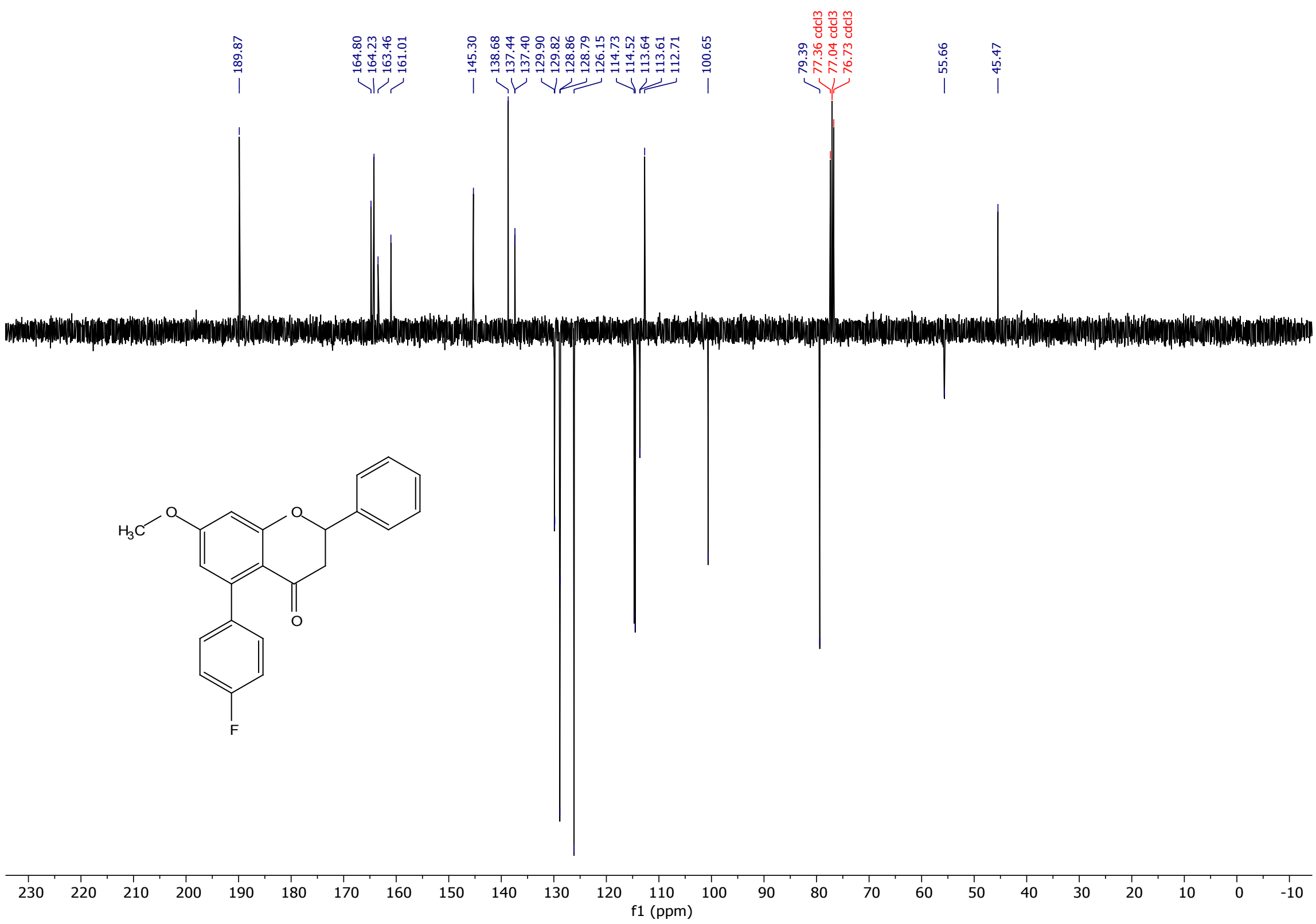

Figure S32. ^{1}H and ^{13}C APT NMR spectra of compound **2g**

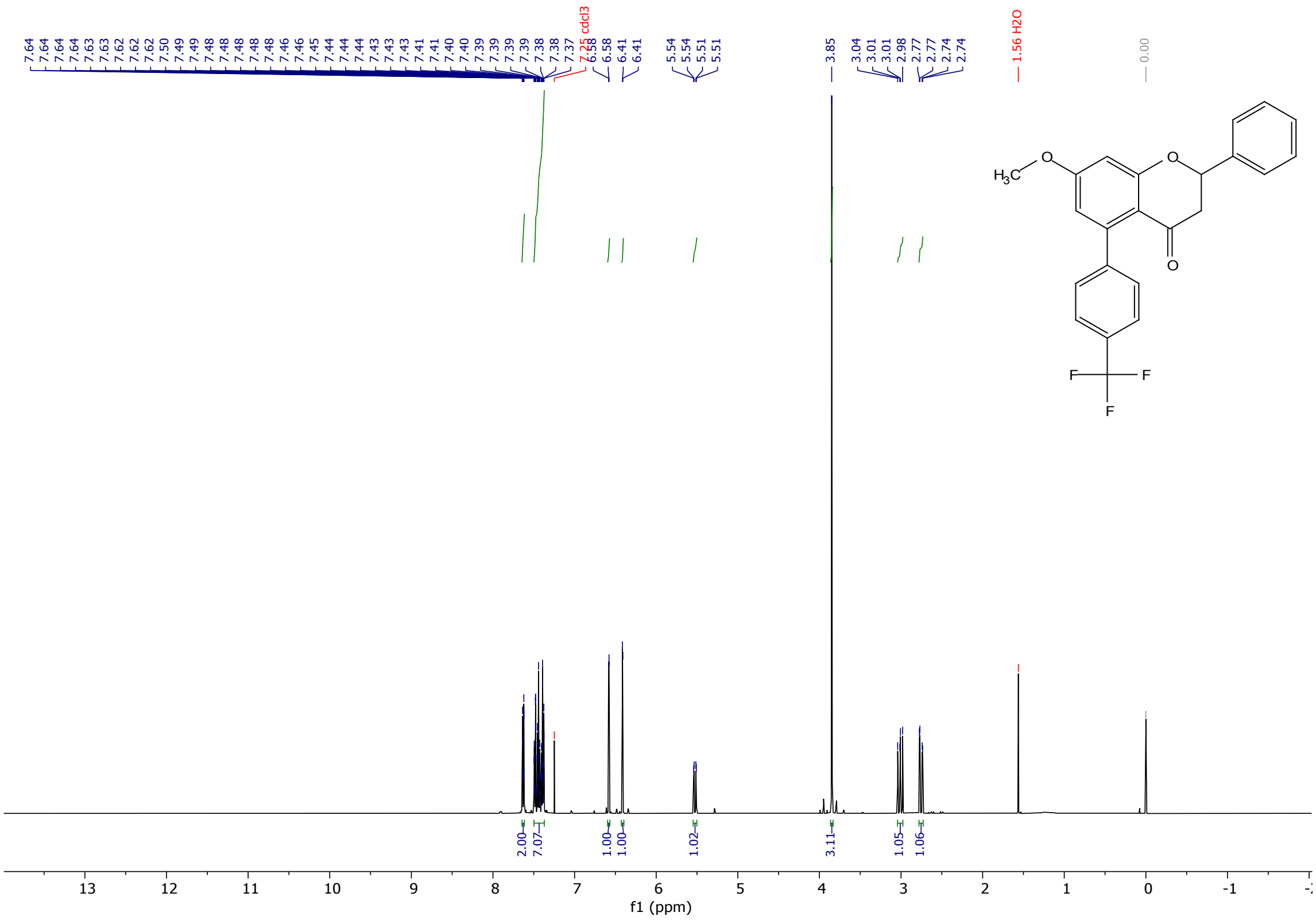

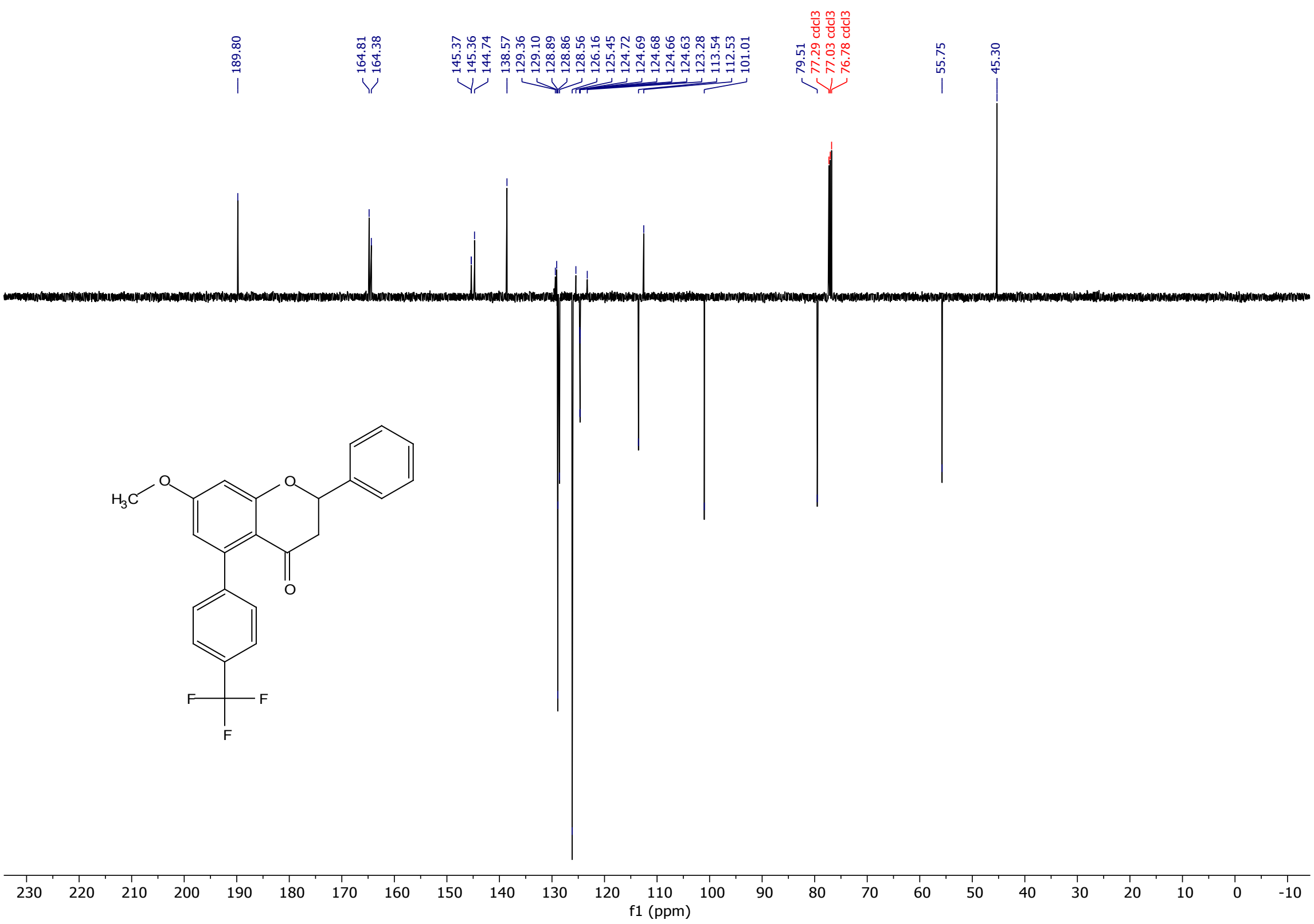

Figure S33. ¹H and ¹³C APT NMR spectra of compound **2h**

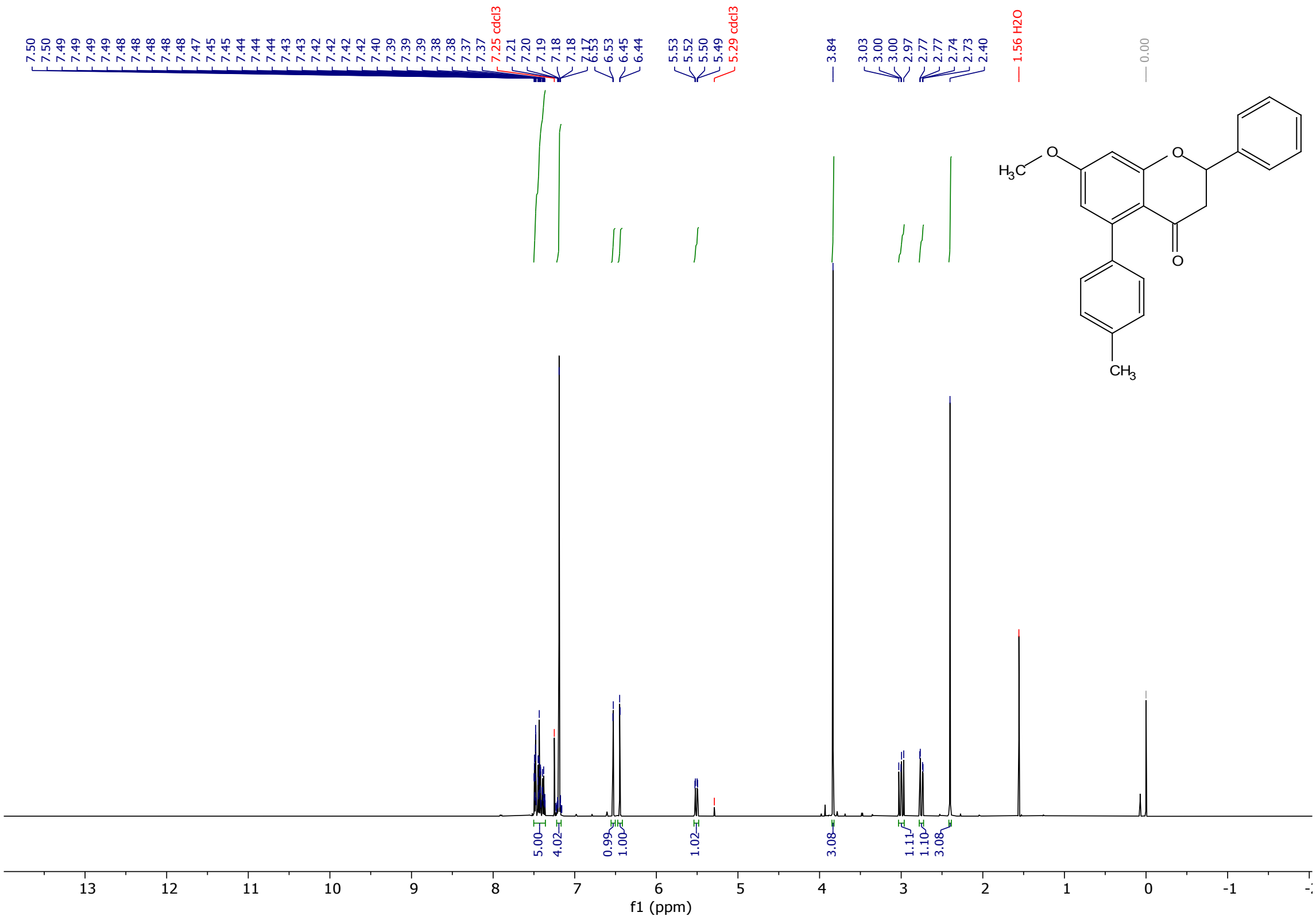

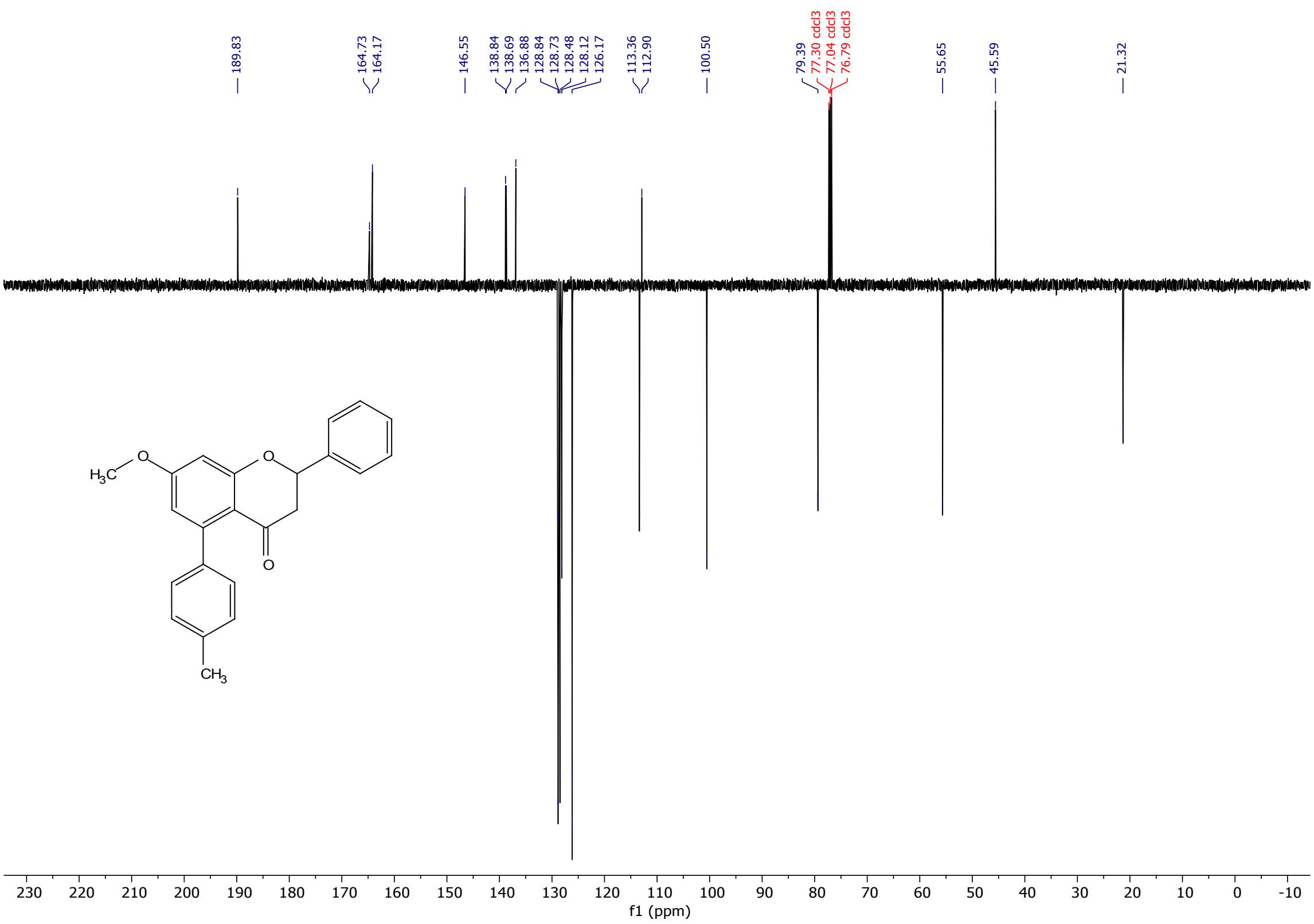

Figure S34. ^{1}H and ^{13}C APT NMR spectra of compound **2i**

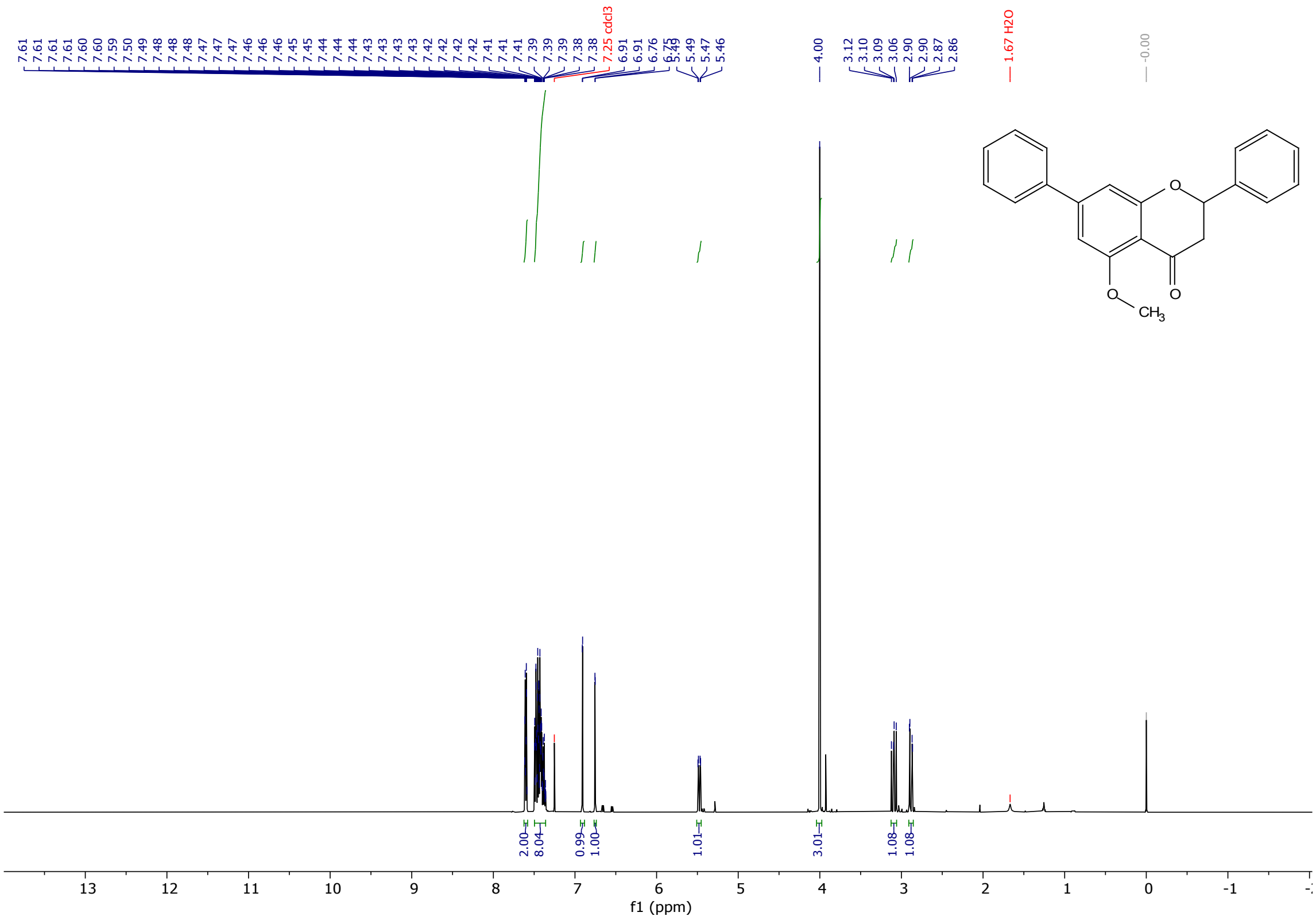

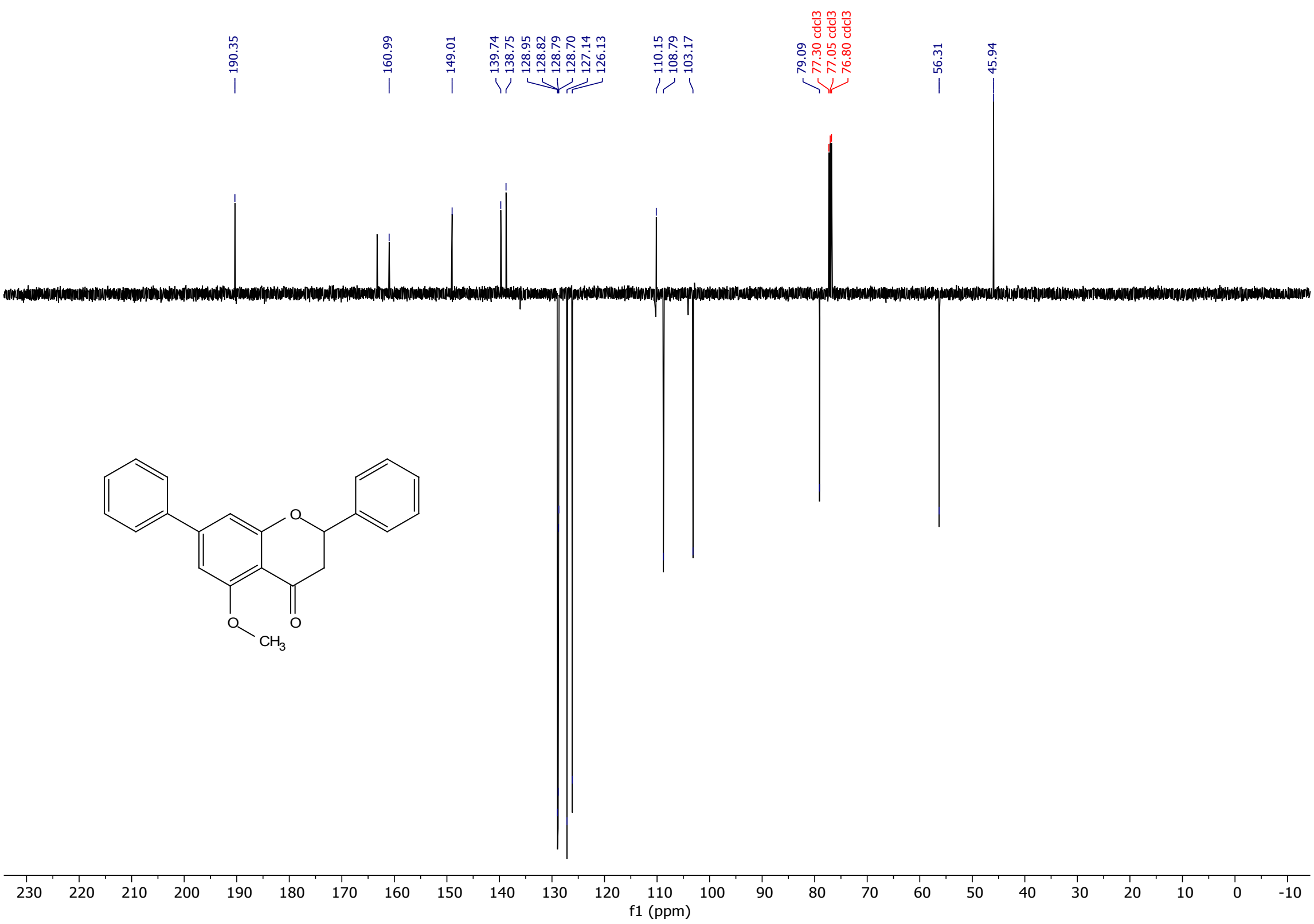

Figure S35. ^{1}H and ^{13}C APT NMR spectra of compound **2j**

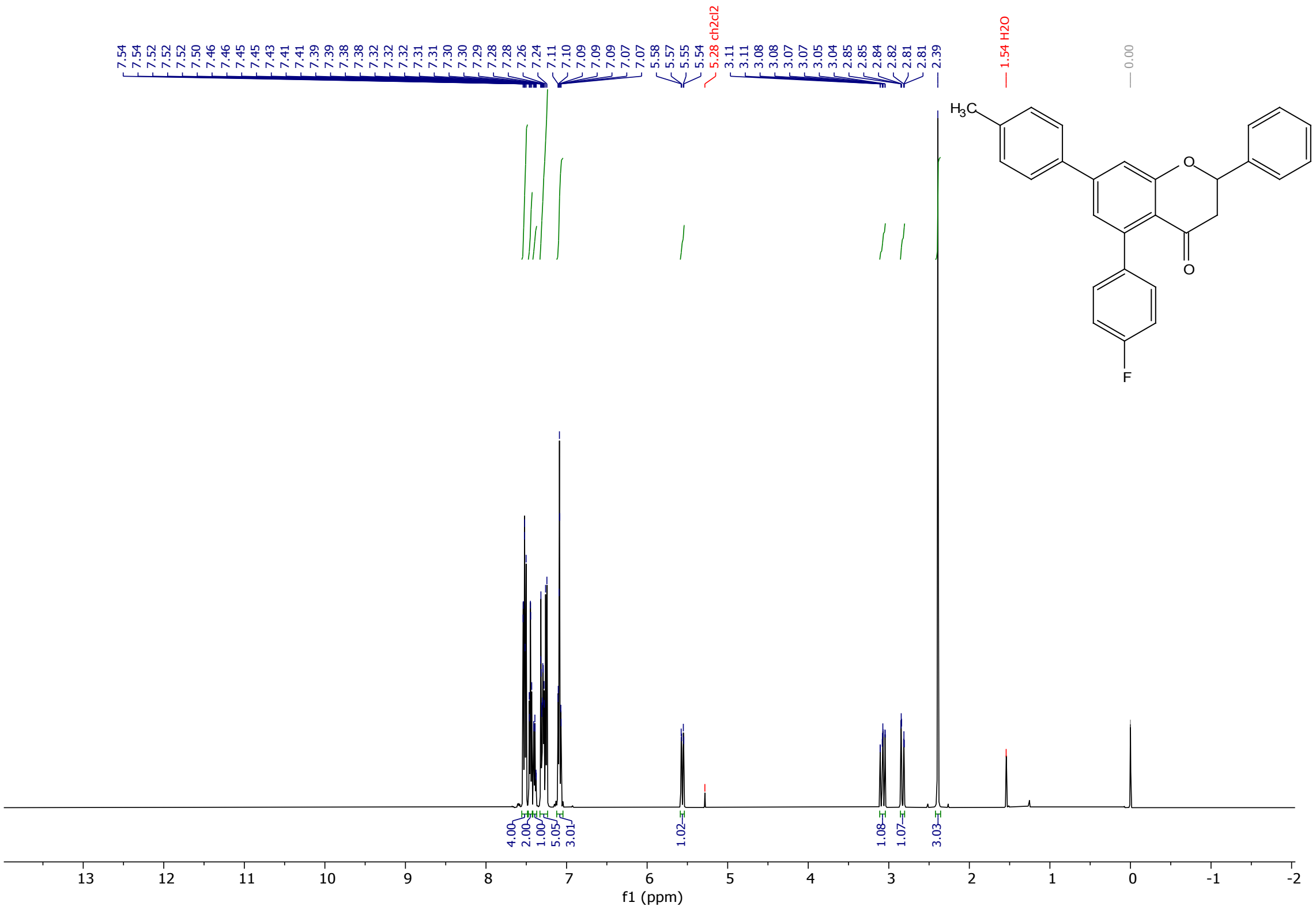

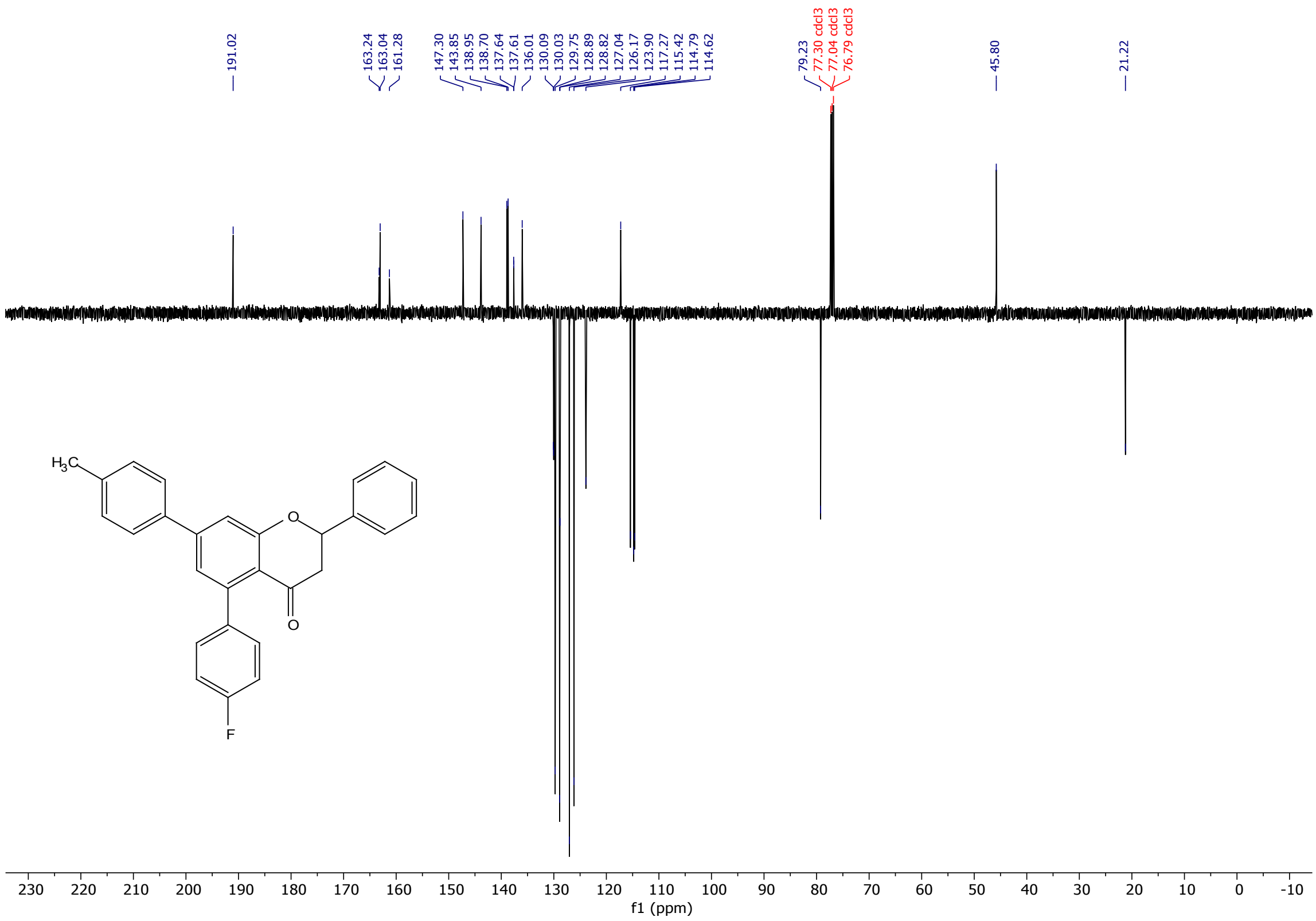

Figure S36. ^{1}H and ^{13}C APT NMR spectra of compound **3a/Pinostrobin chalcone**

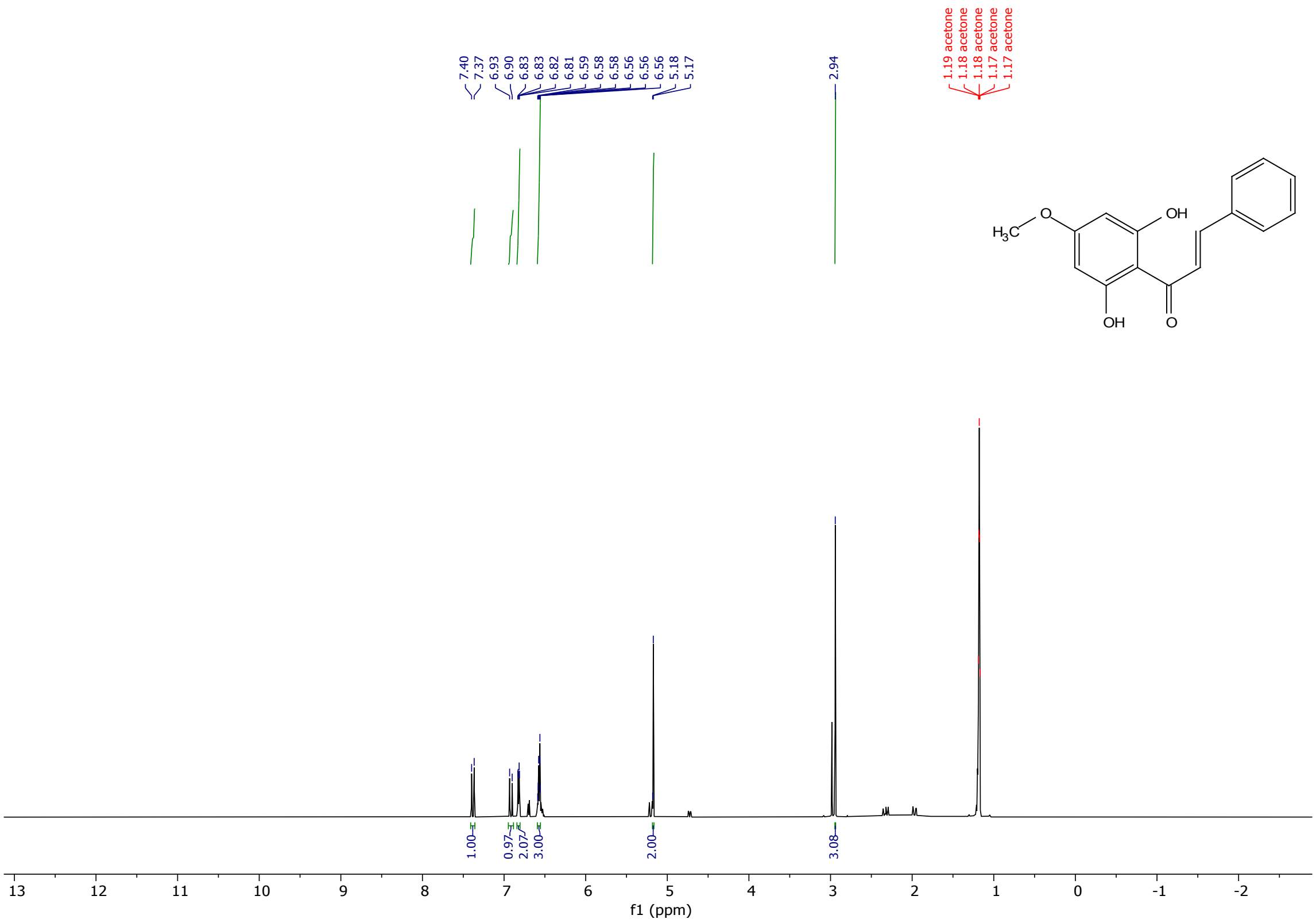

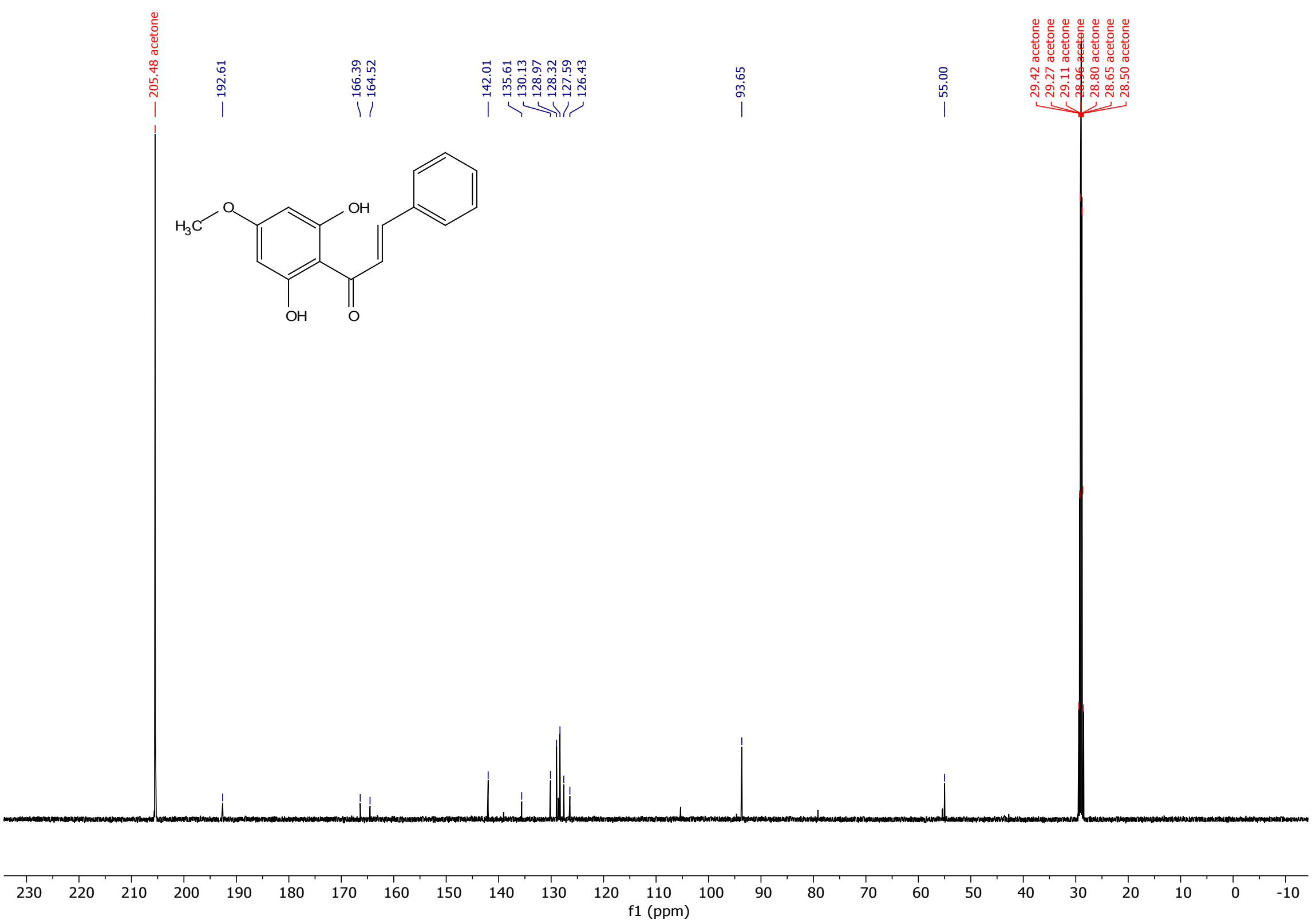

Figure S37. ^{1}H and ^{13}C APT NMR spectra of compound **3b/Flavokawain B**

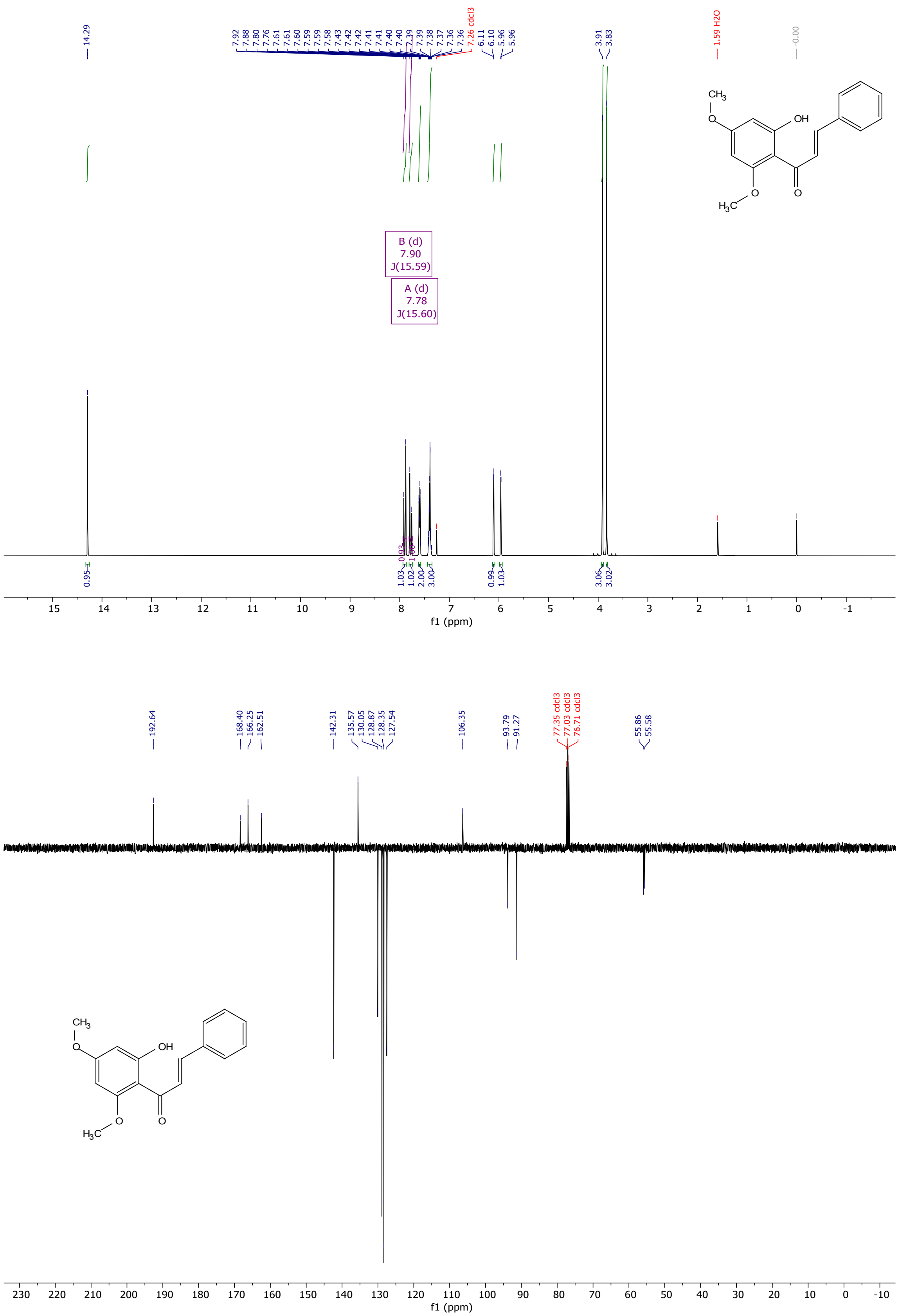

Figure S38. ^{1}H and ^{13}C APT NMR spectra of compound **3c**

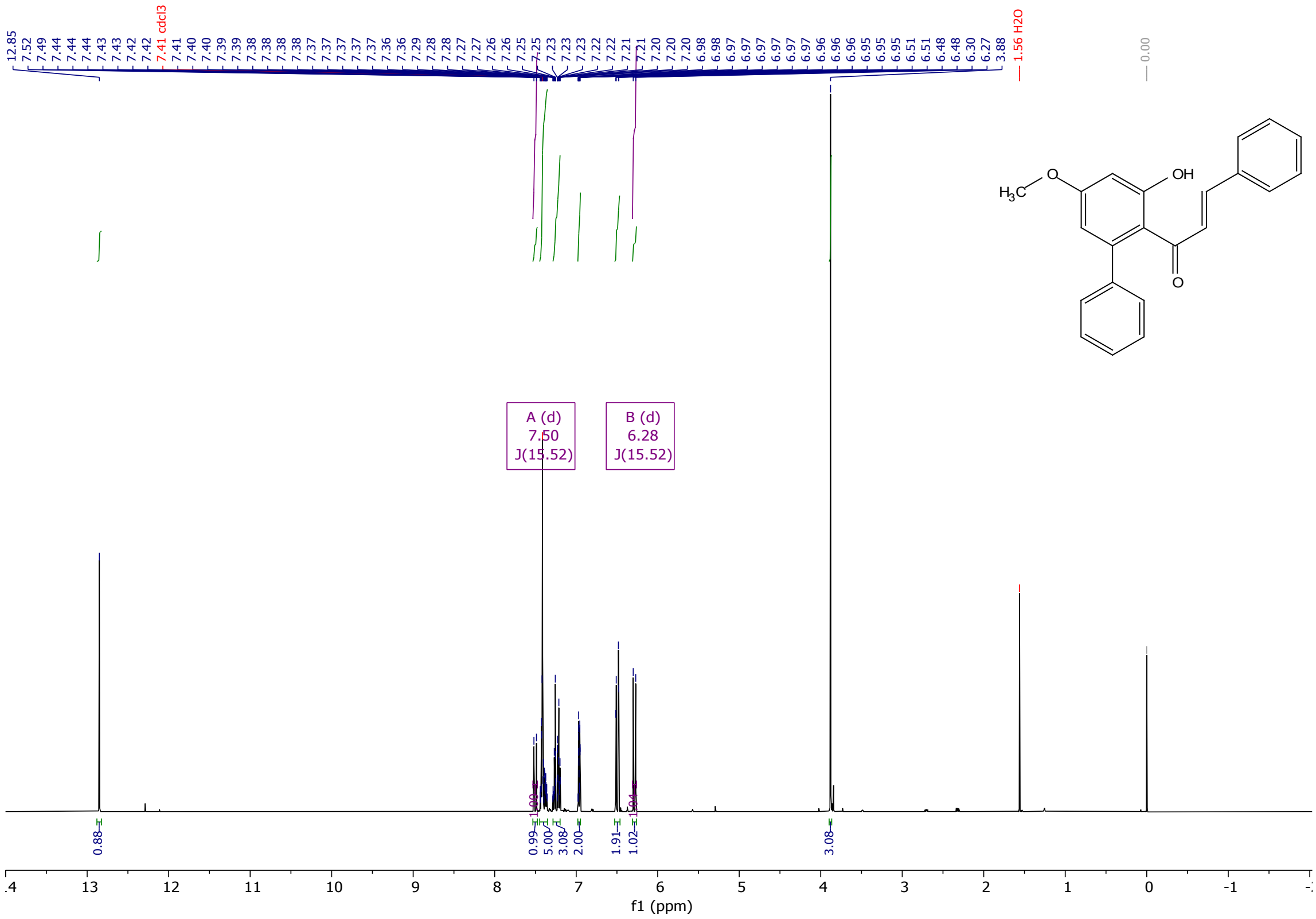

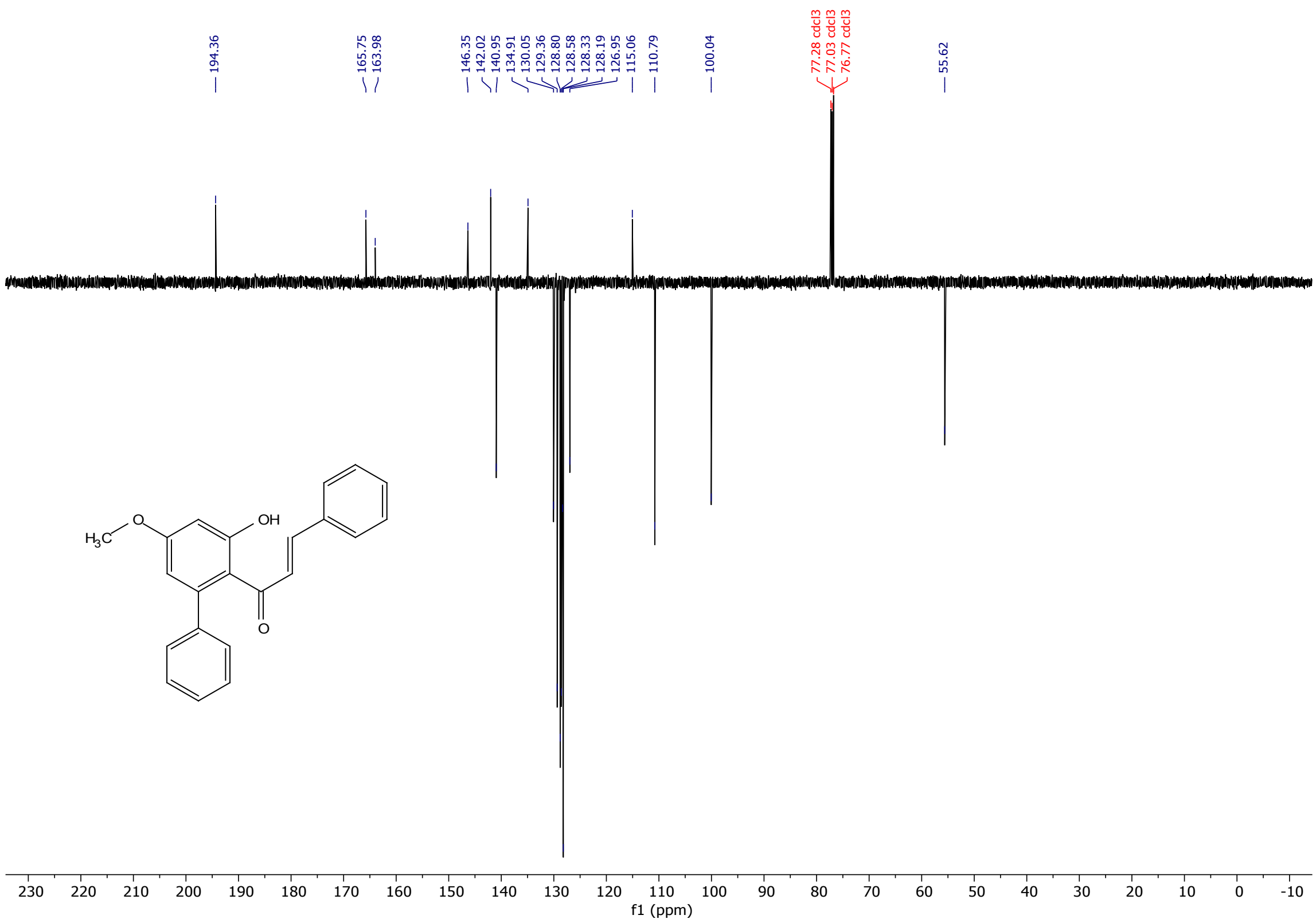

Figure S39. ^{1}H and ^{13}C APT NMR spectra of compound **3d**

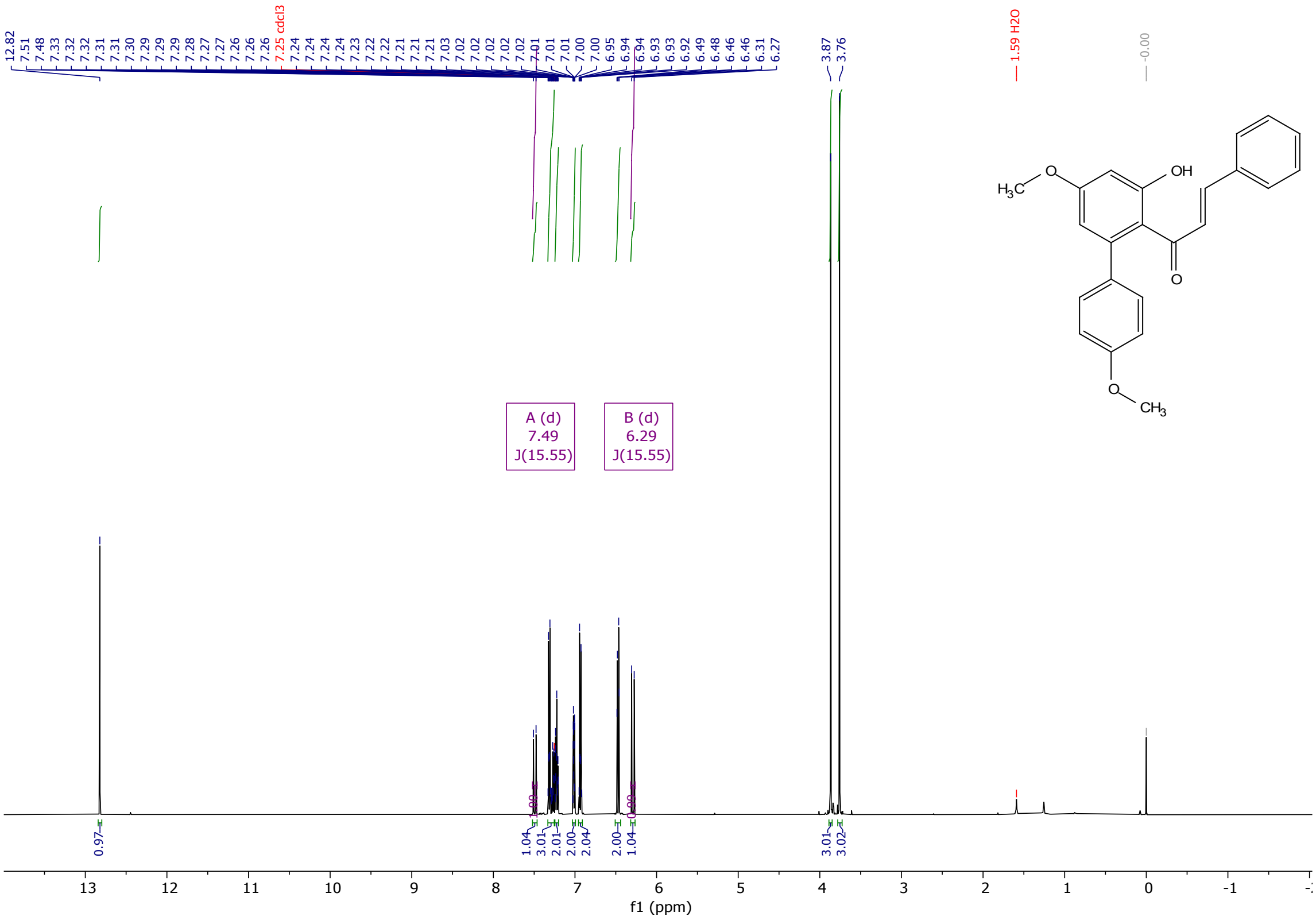

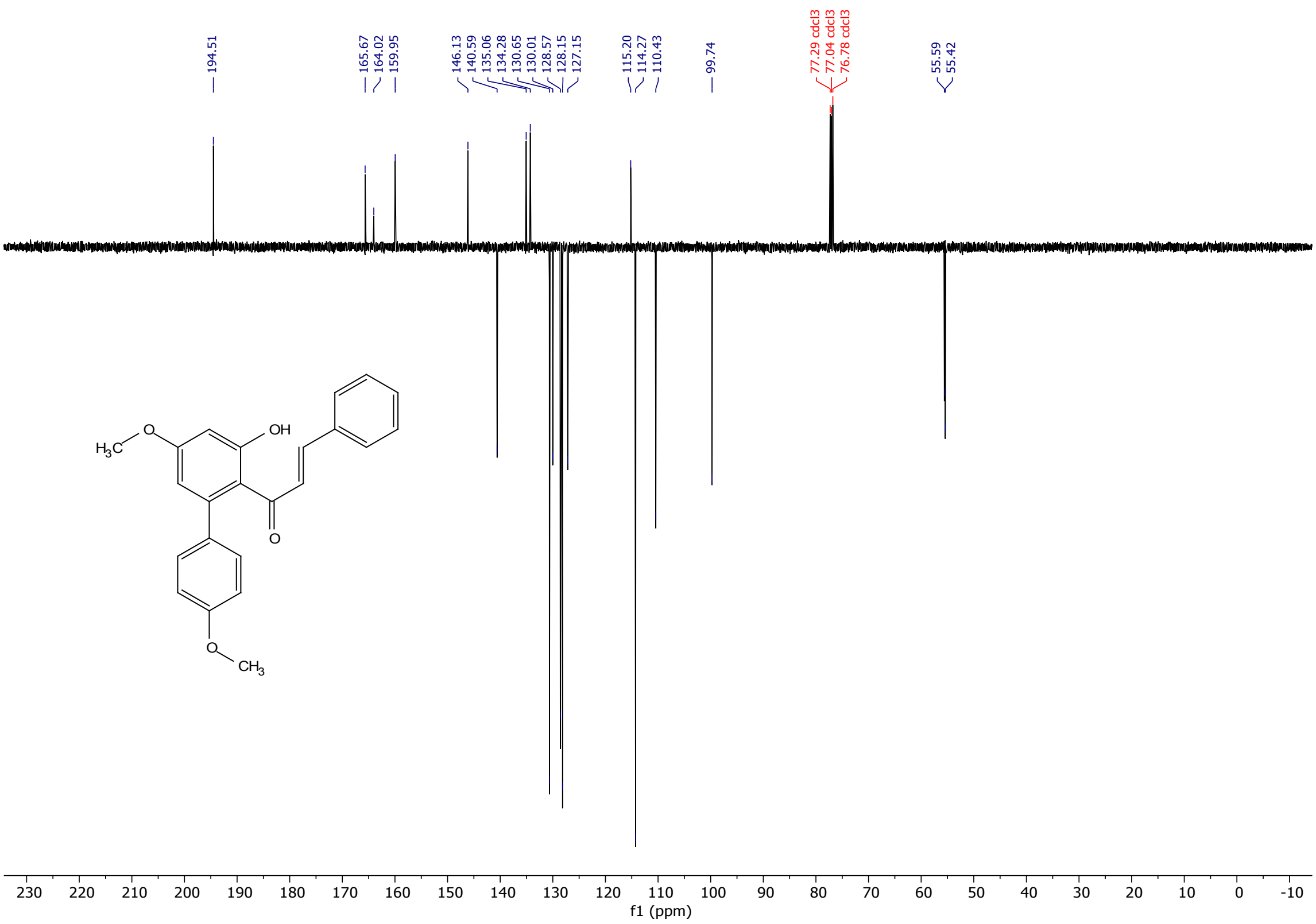

Figure S40. ^{1}H and ^{13}C APT NMR spectra of compound **3e**

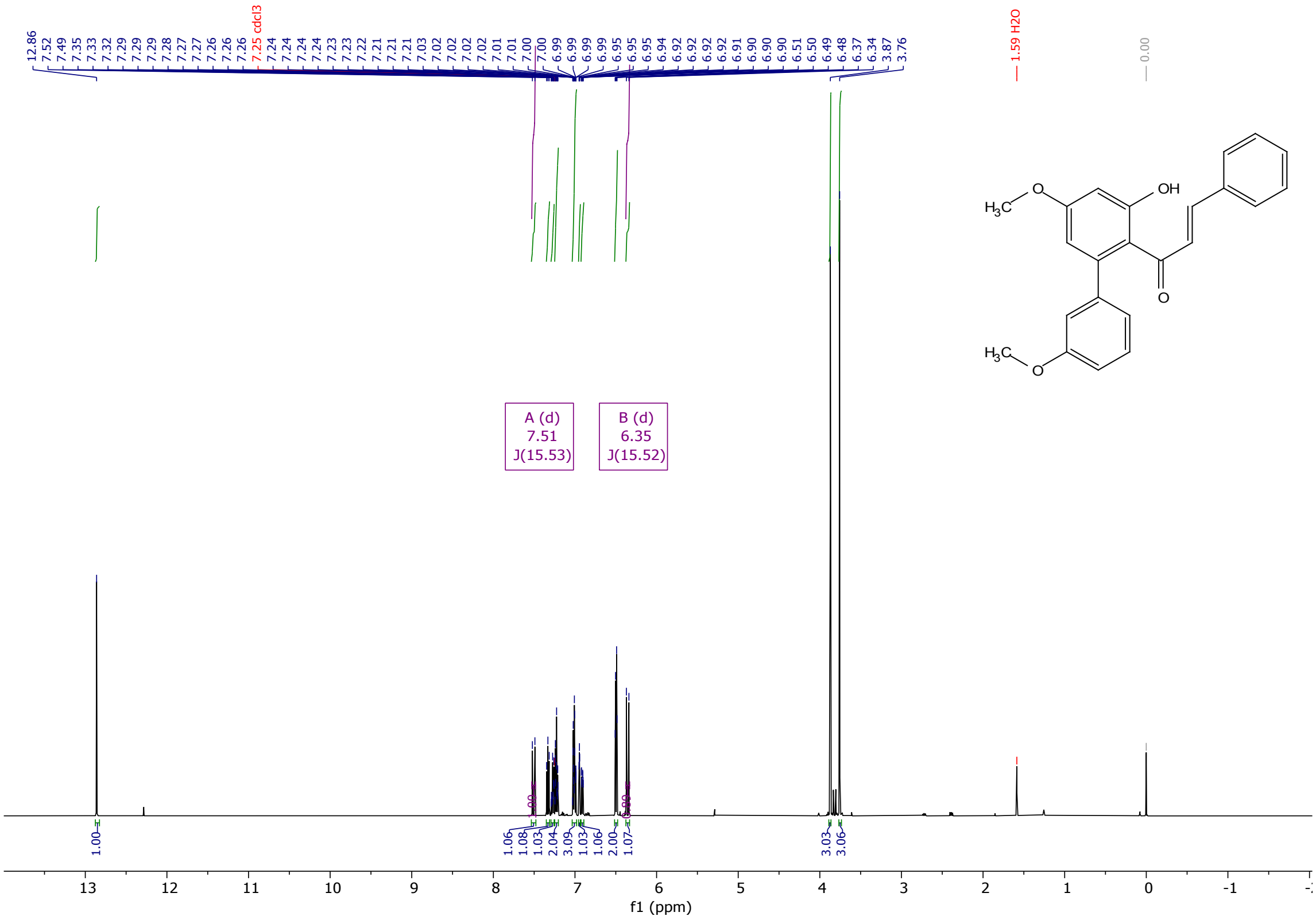

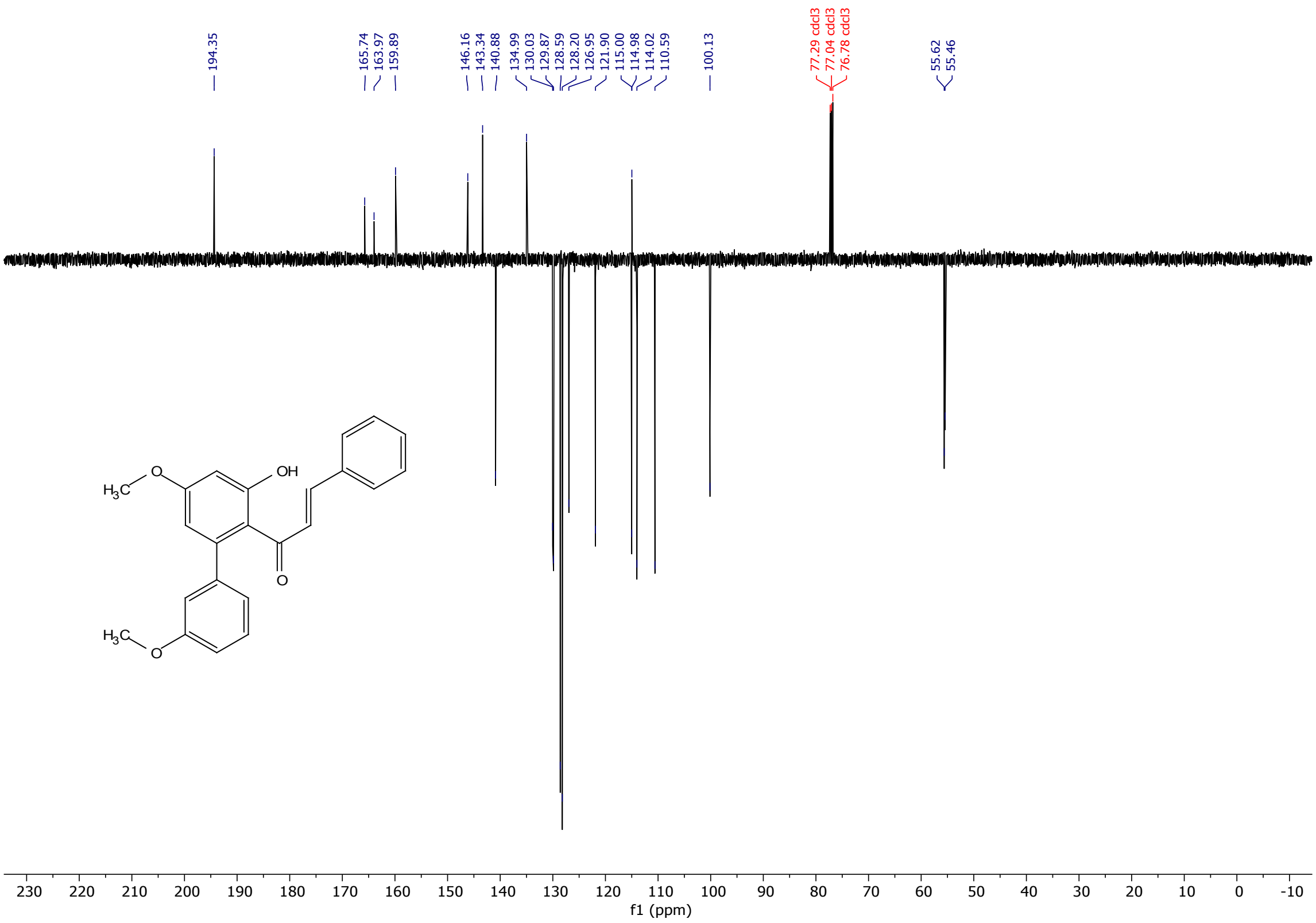

Figure S41. ^{1}H and ^{13}C APT NMR spectra of compound **3f**

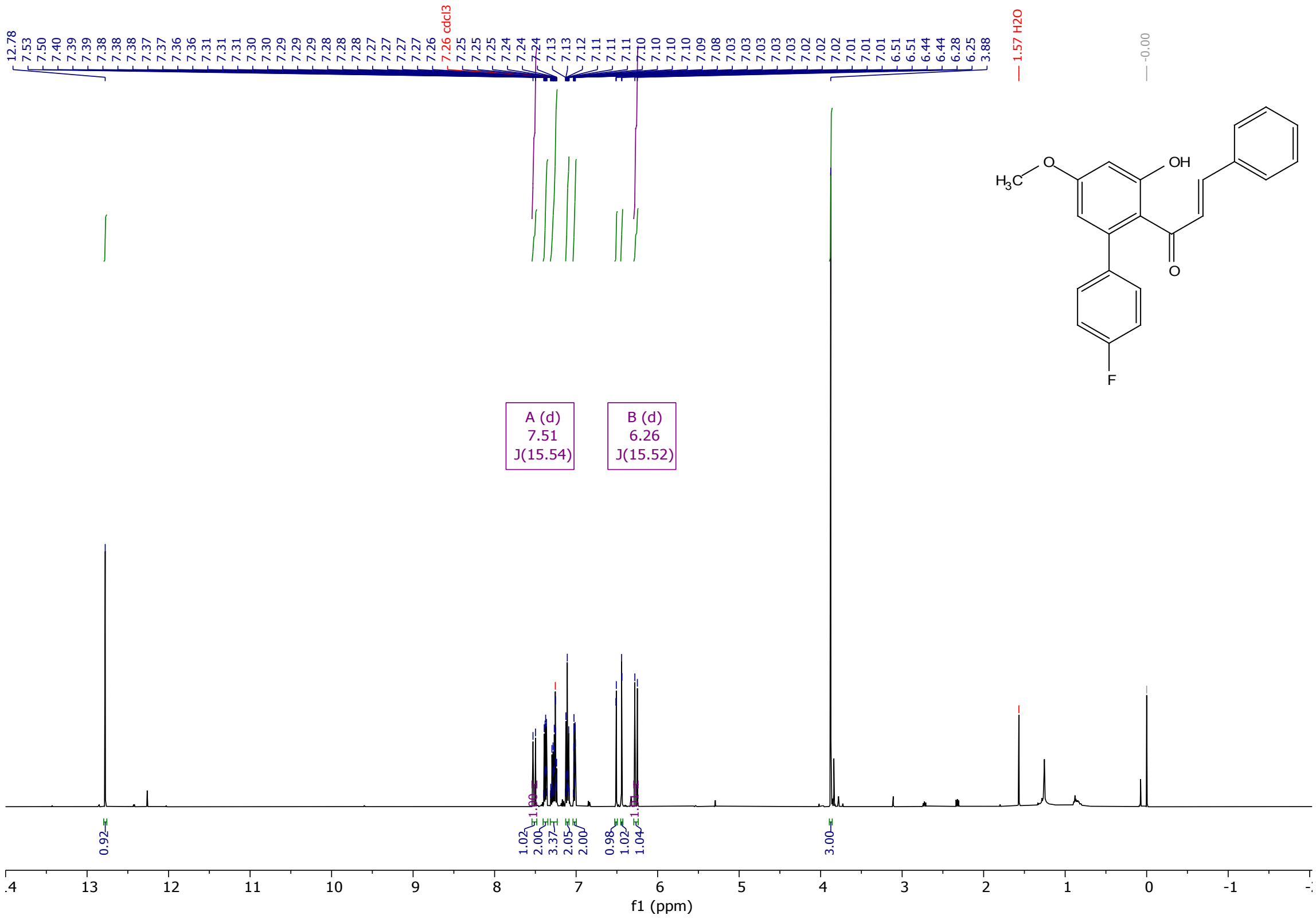

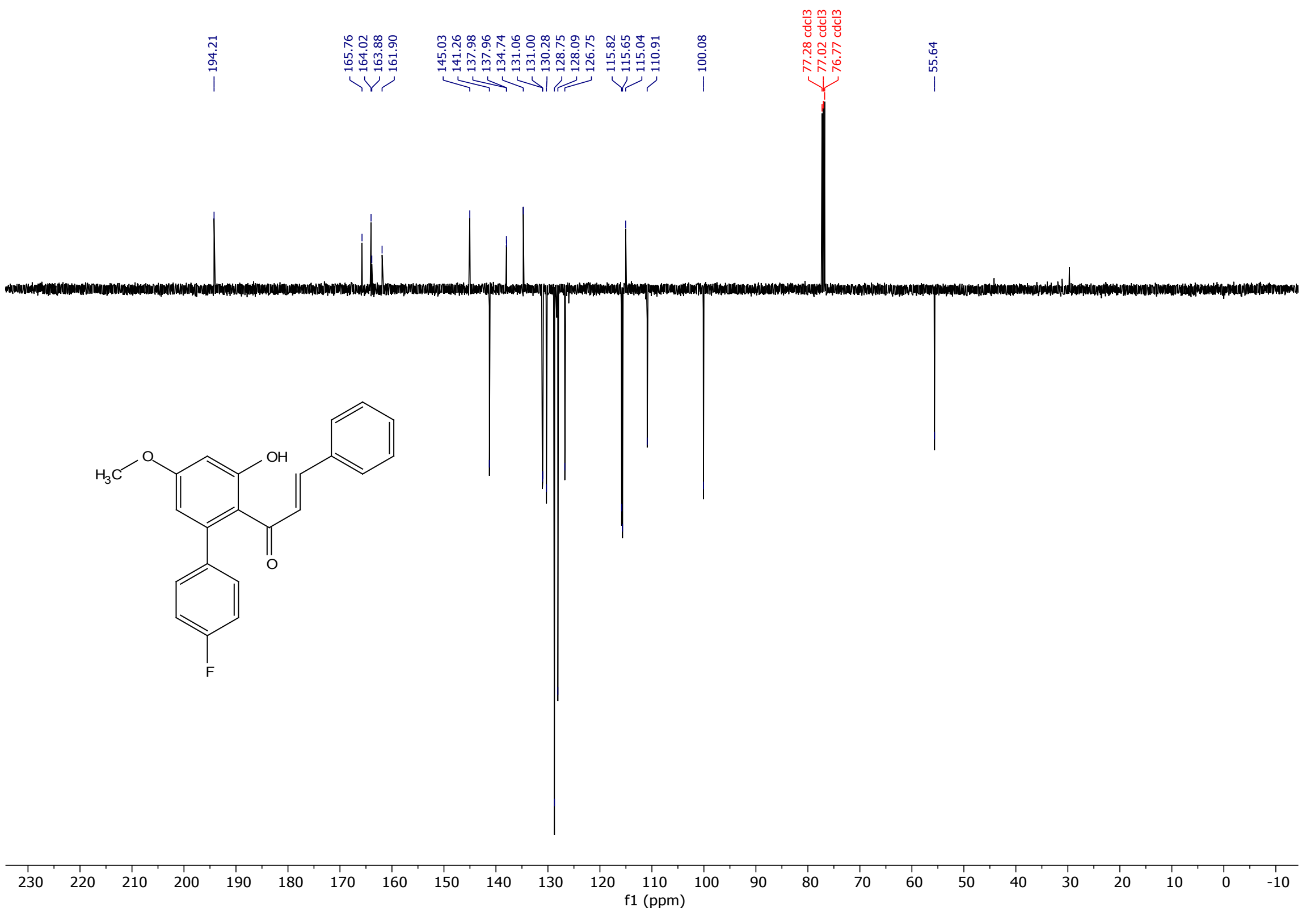

Figure S42. ^{1}H and ^{13}C APT NMR spectra of compound **3g**

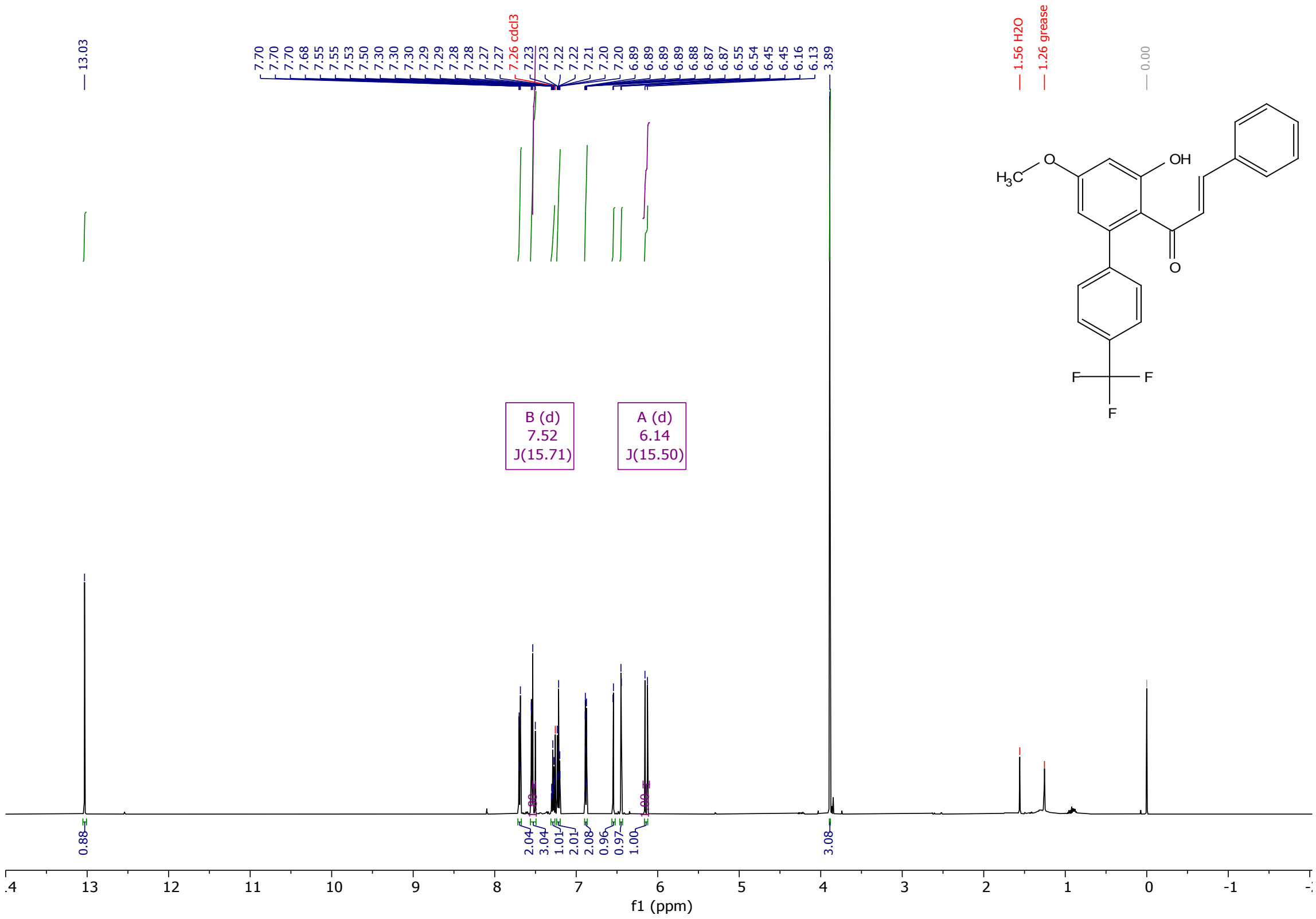

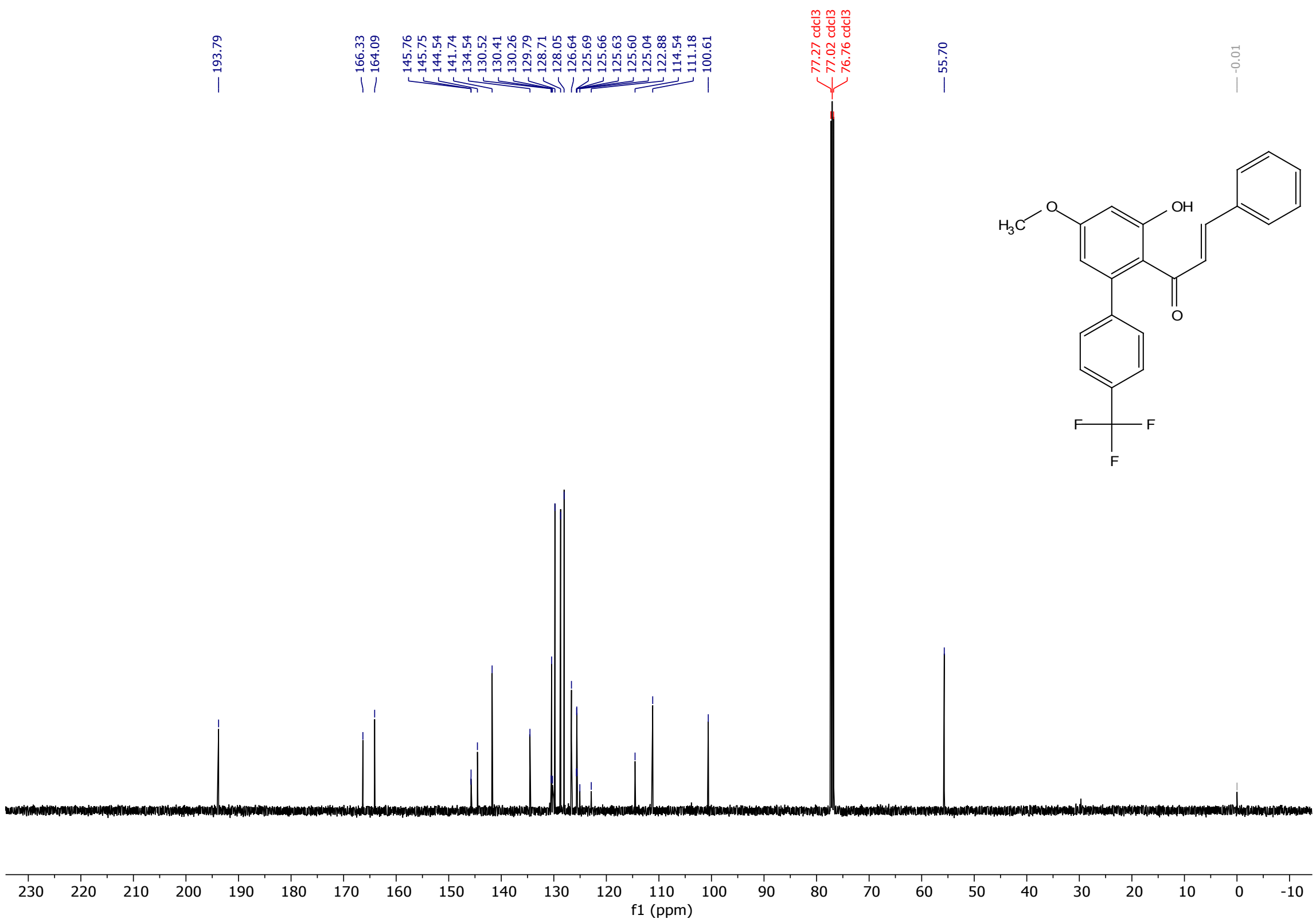

Figure S43. ^{1}H and ^{13}C APT NMR spectra of compound **3h**

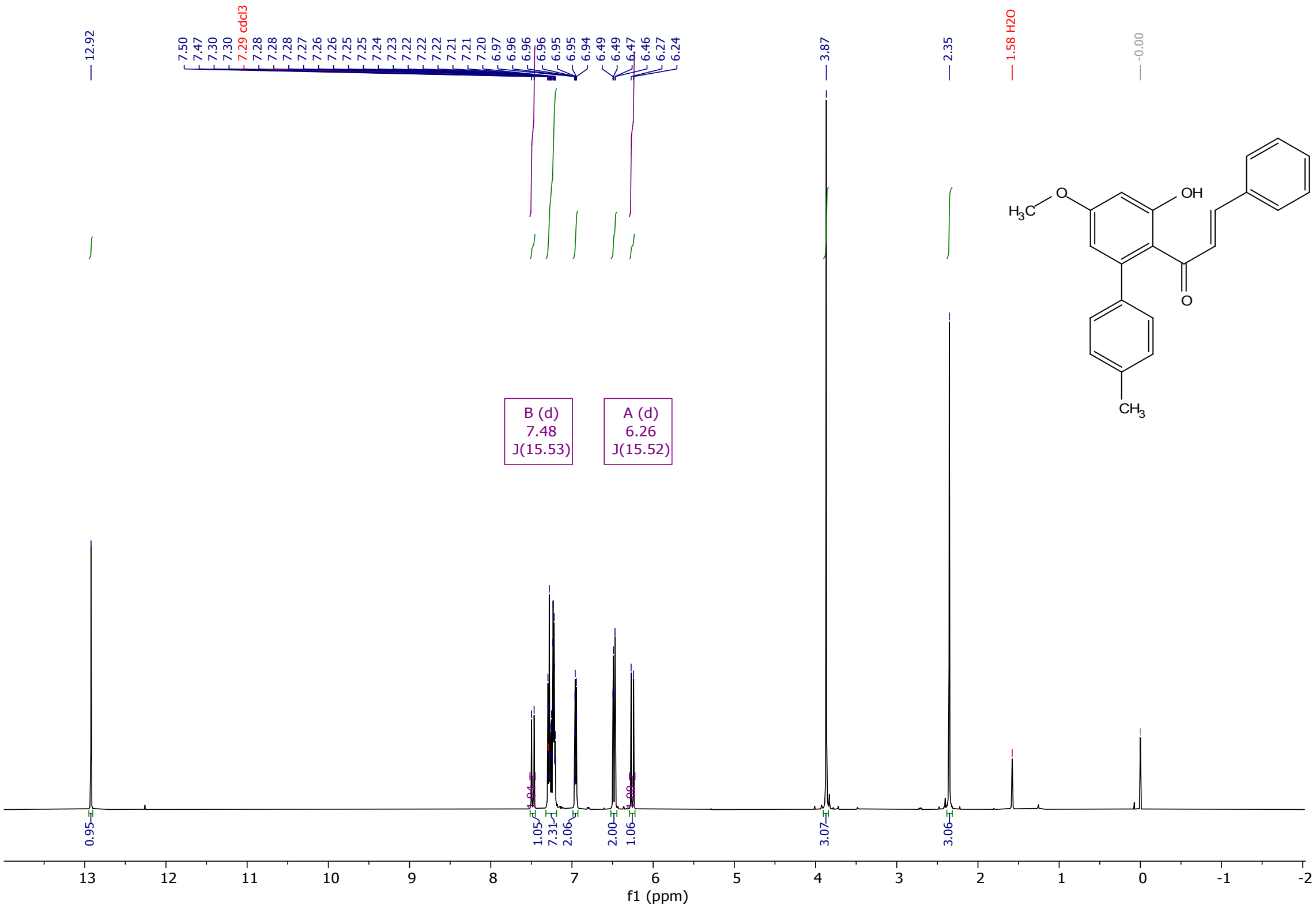

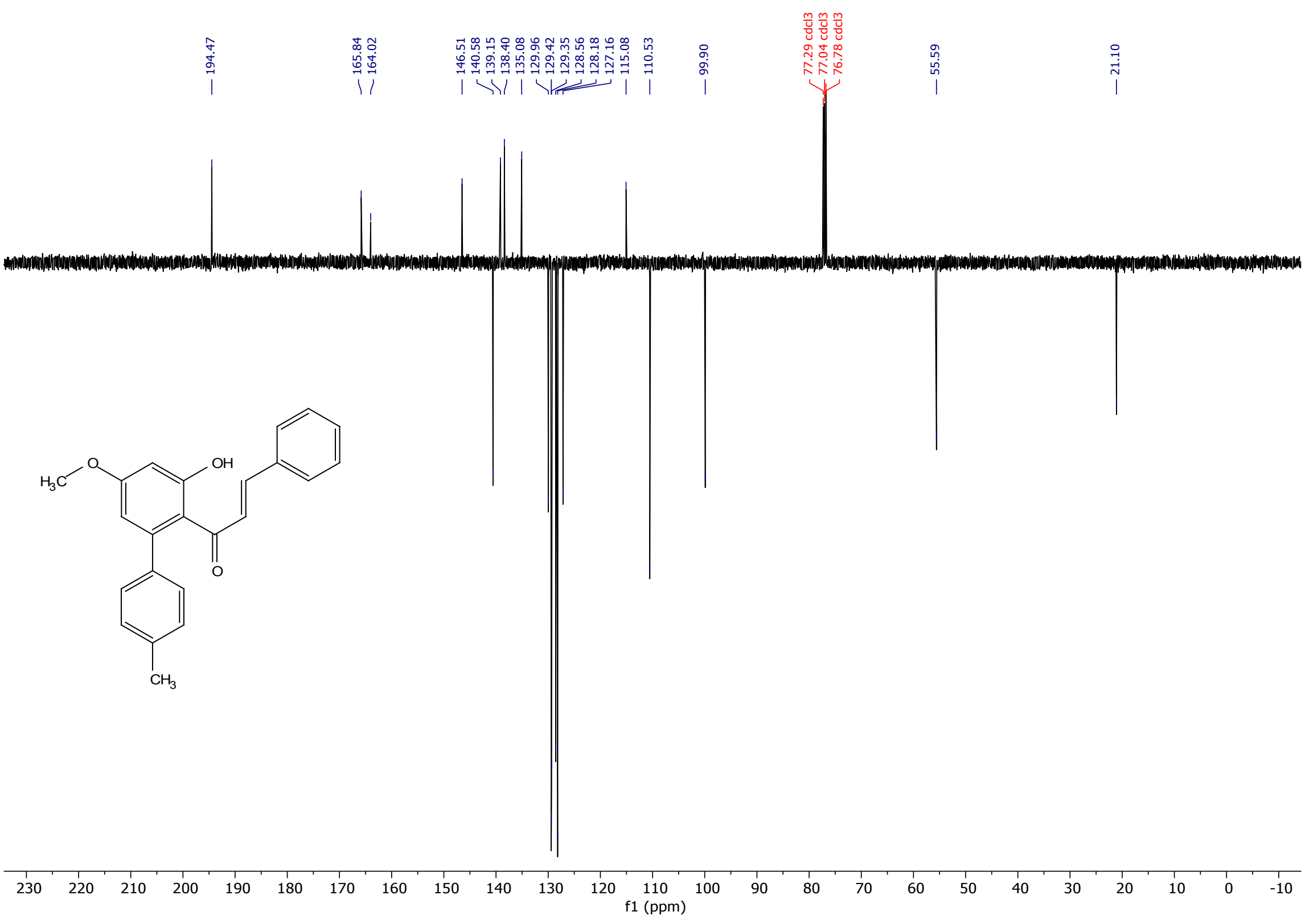

Figure S44. ^{1}H and ^{13}C APT NMR spectra of compound **3i**

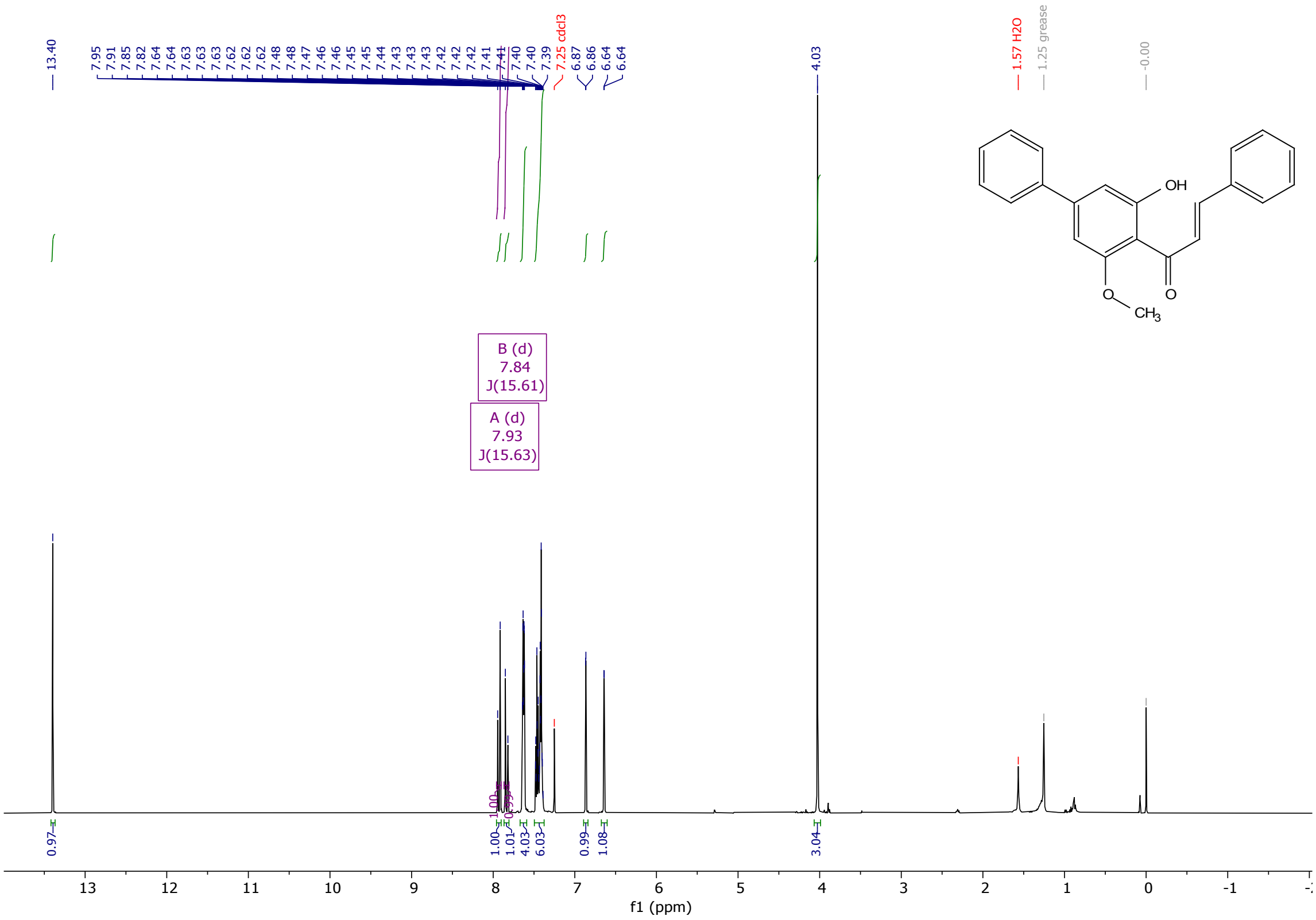

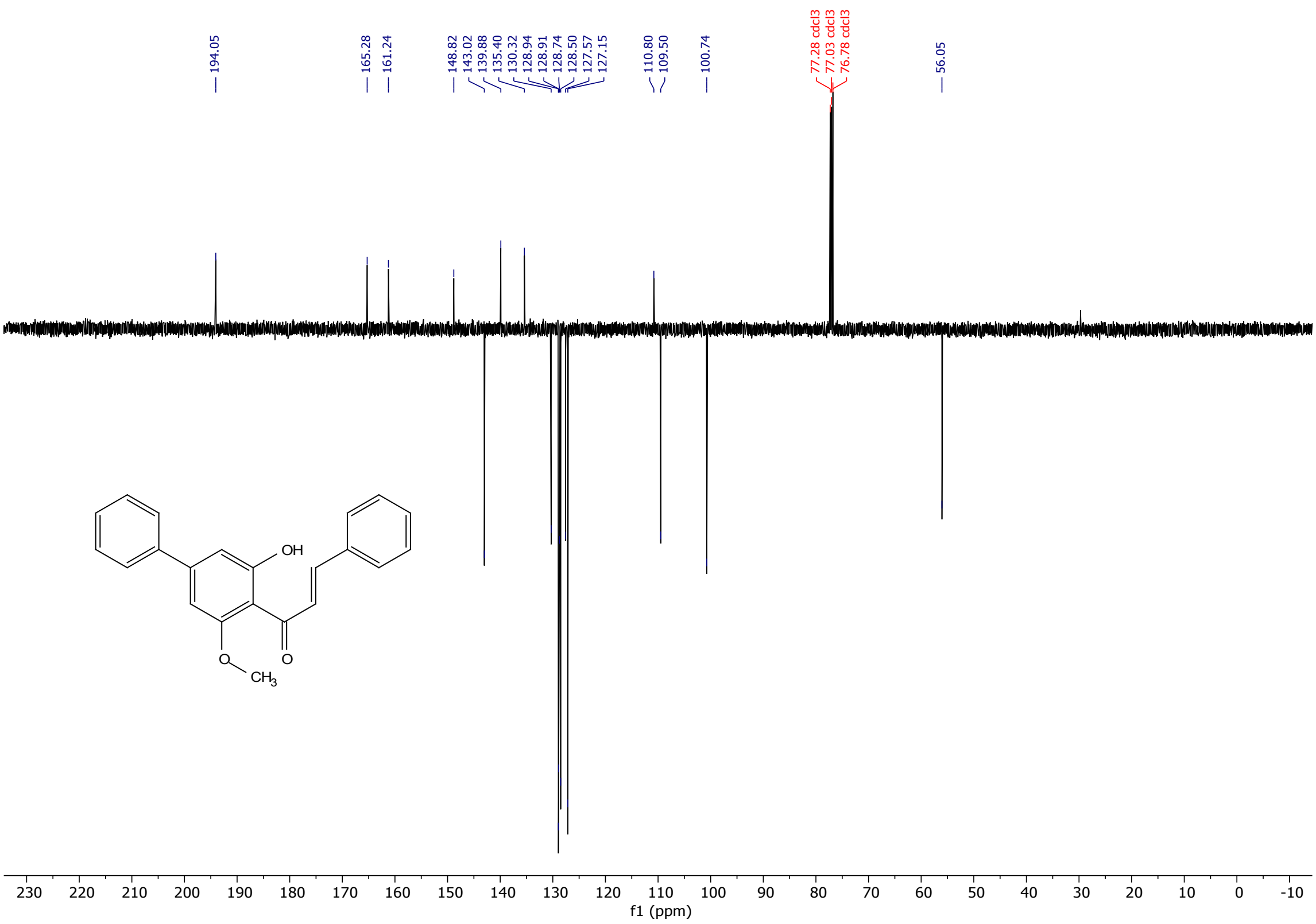

Figure S45. ^{1}H and ^{13}C APT NMR spectra of compound **3j**

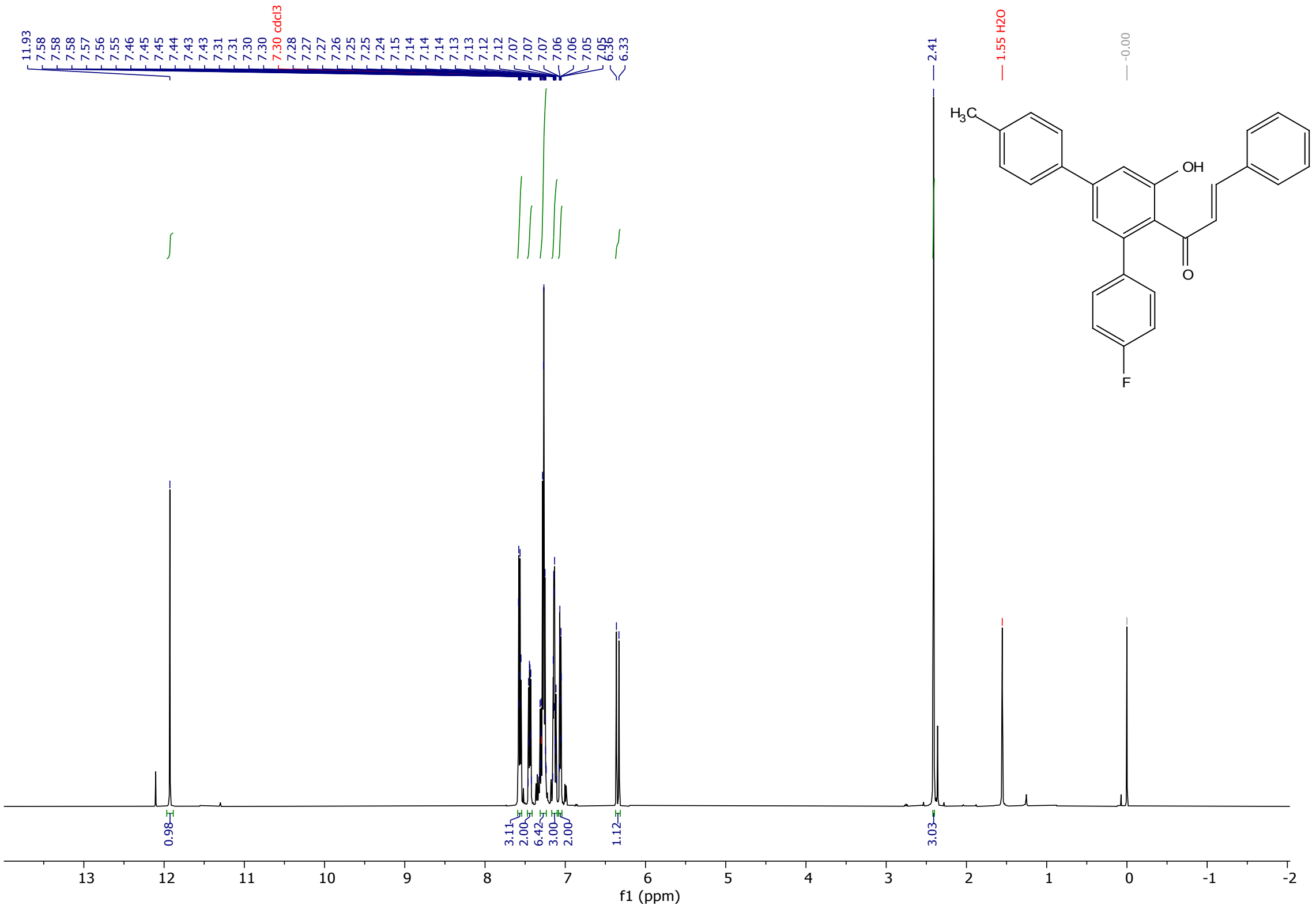

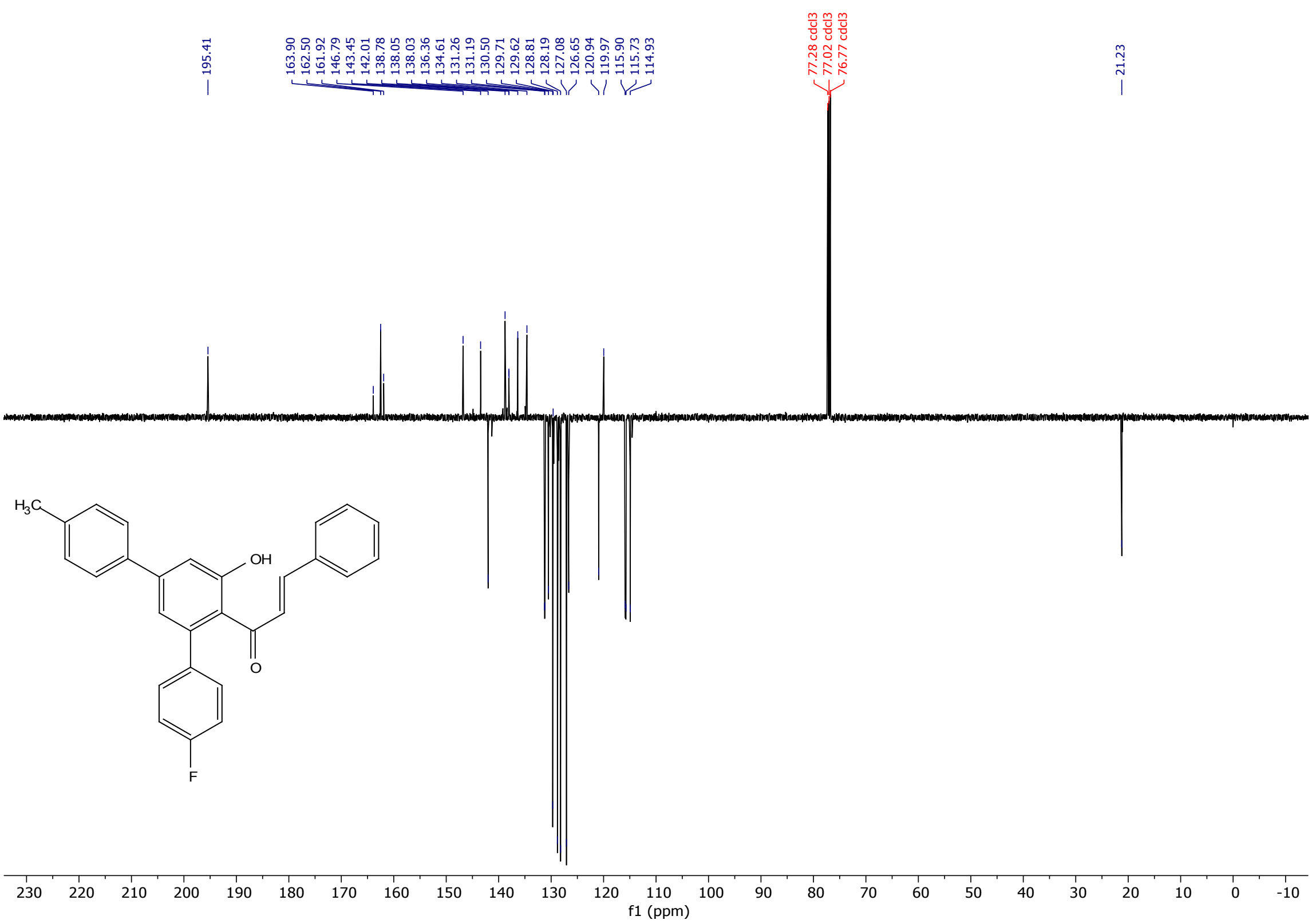

Figure S46. ^{1}H and ^{13}C APT NMR spectra of compound **2′,4′,6′-trimethoxy-chalcone (TMC)**

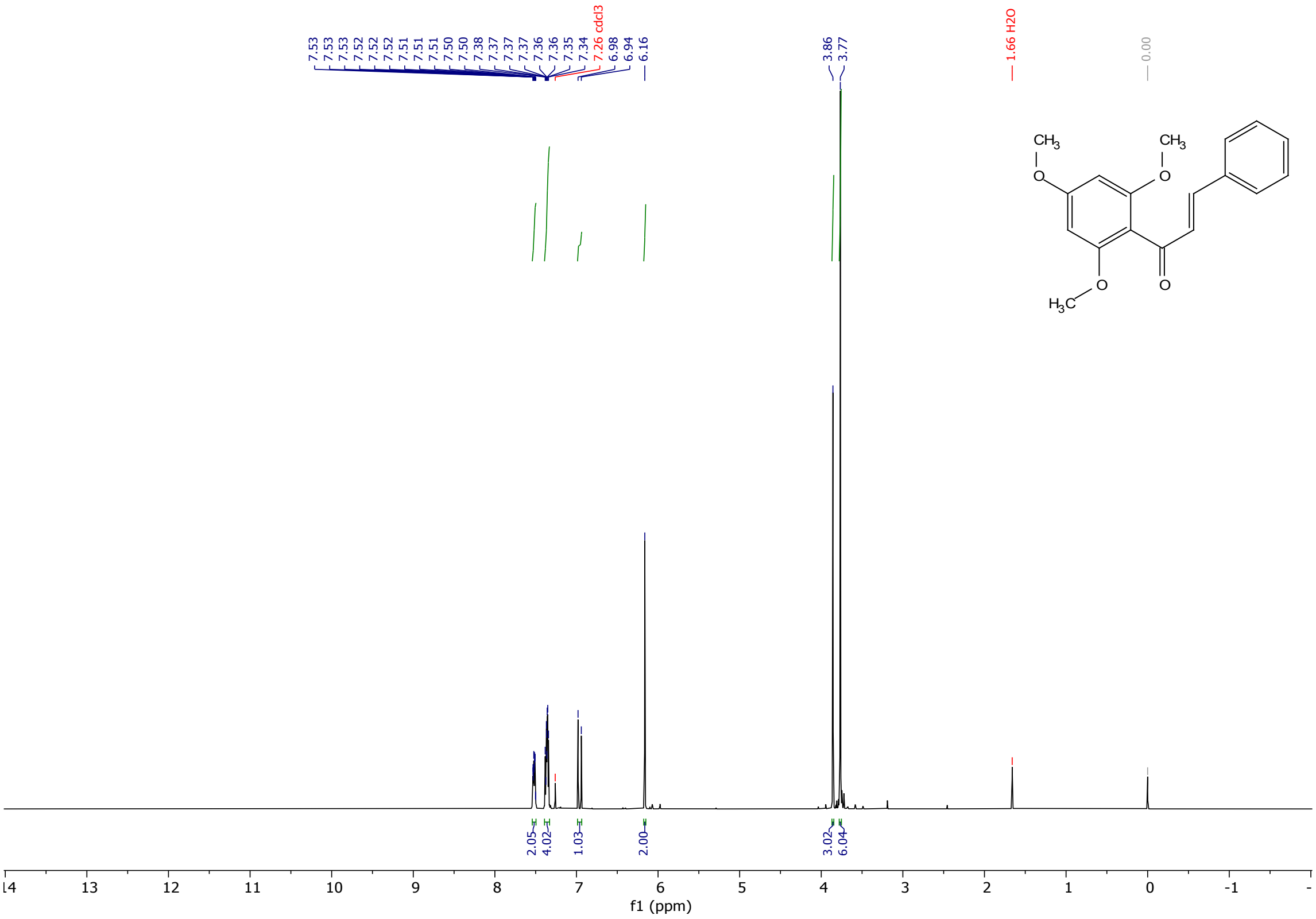

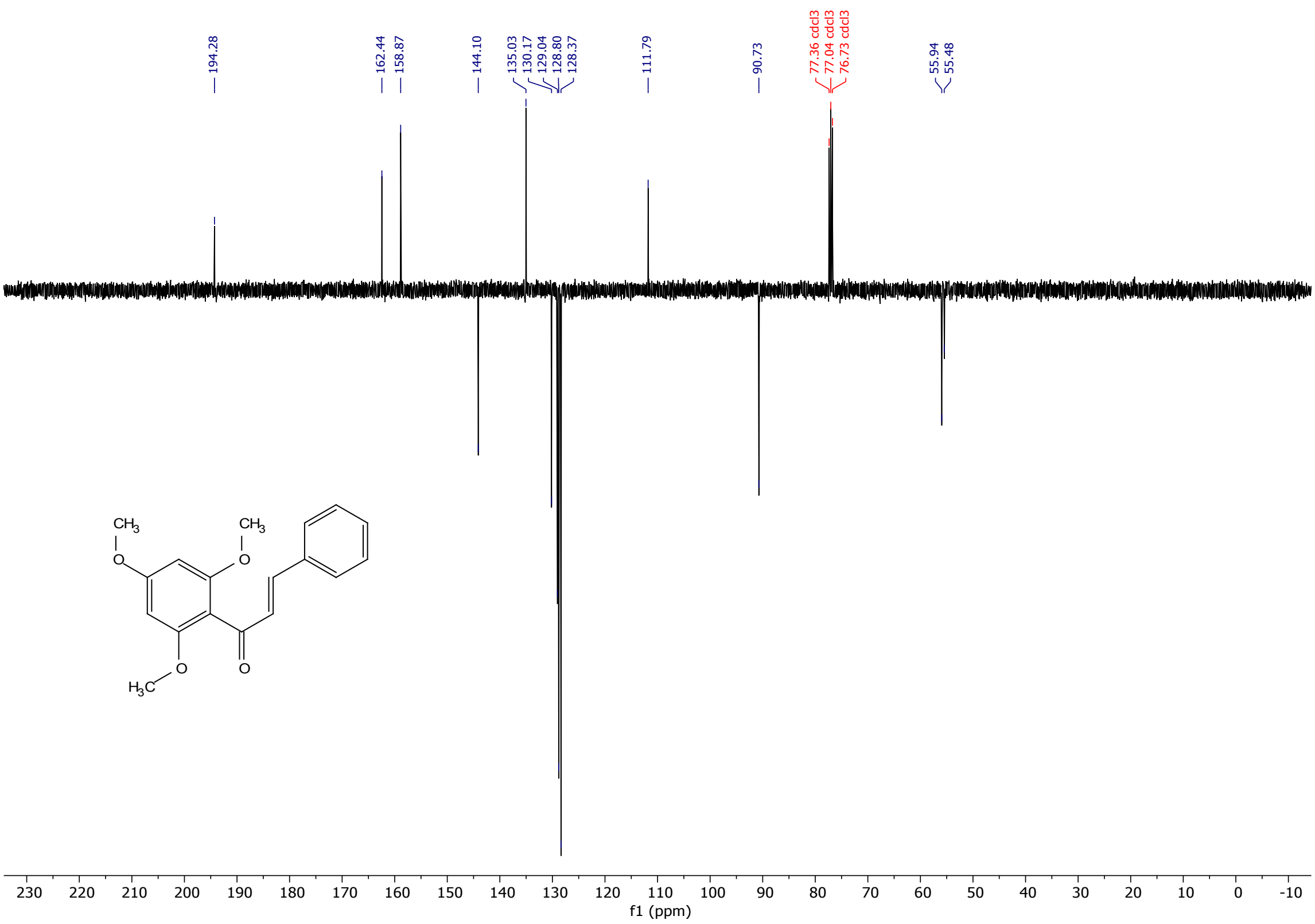

6. Biochemistry and Biological Assays

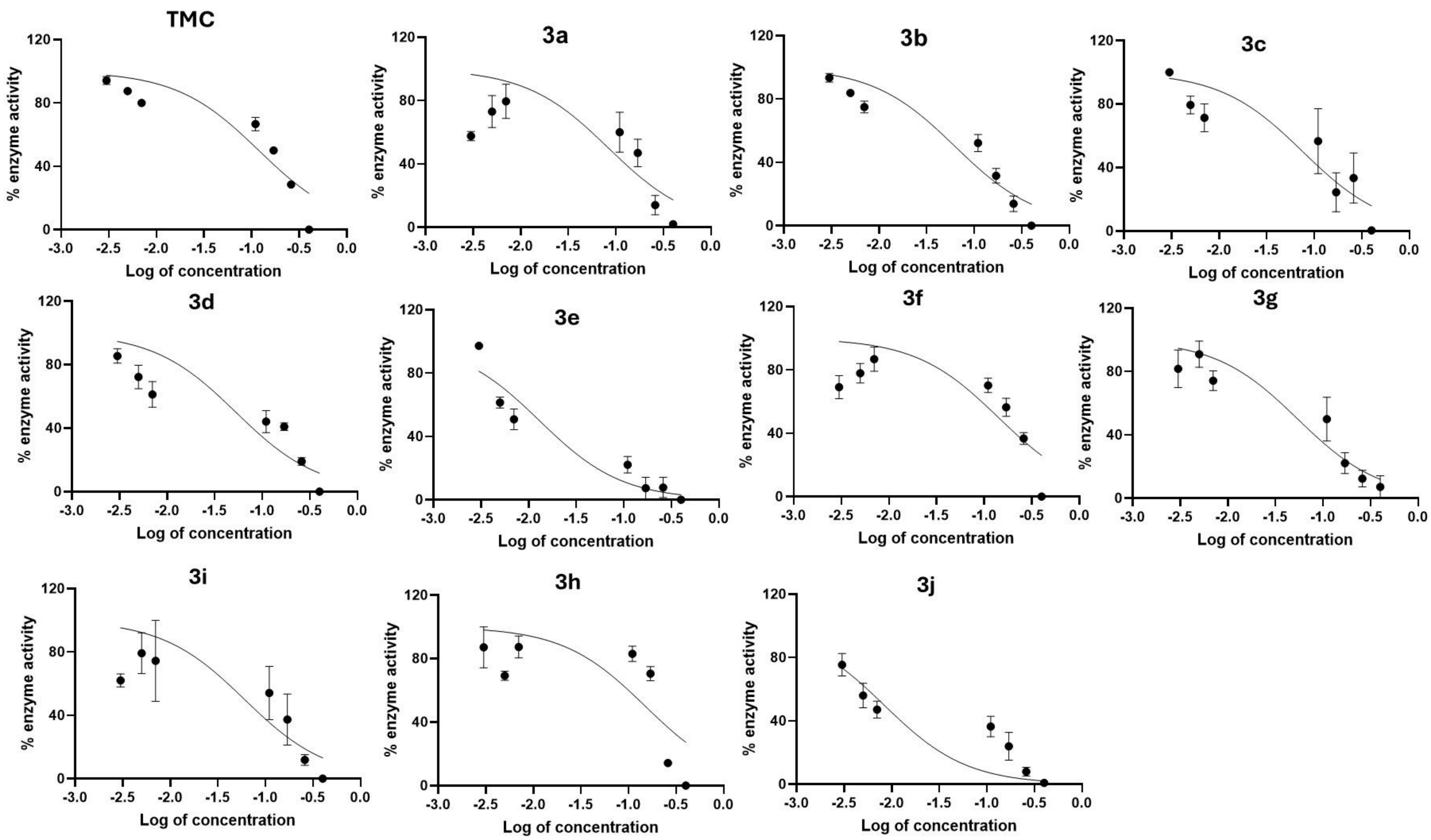

Figure S47. Proteolytic inhibition assays of chalcone derivatives in Tris-HCl buffer

Assays were performed using the fluorogenic peptide substrate Z-Phe-Arg-7-amido-4-methylcoumarin (Z-FR-AMC, 1 mM) and promastigote protein extract (5 µg) in 10 mM Tris-HCl (pH 7.5). The following chalcone compounds were analyzed: **2′,4′,6′-trimethoxychalcone (TMC)**, **pinostrobin chalcone (3a)**, **flavokawain B (3b)**, and derivatives **3c–3j**. The sigmoidal curves indicate the IC50 values (half-maximal inhibitory concentration), representing the dose-response relationship. The distribution of experimental data points (●) shows low inhibition at lower concentrations, a sharp decline in the mid-range, and stabilization at high inhibitory concentrations. Graphs illustrate residual enzymatic activity expressed as a percentage of the control (mean ± SD; %) as a function of the logarithm of the molar concentration (Log of concentration). Data corresponds to three independent experiments.

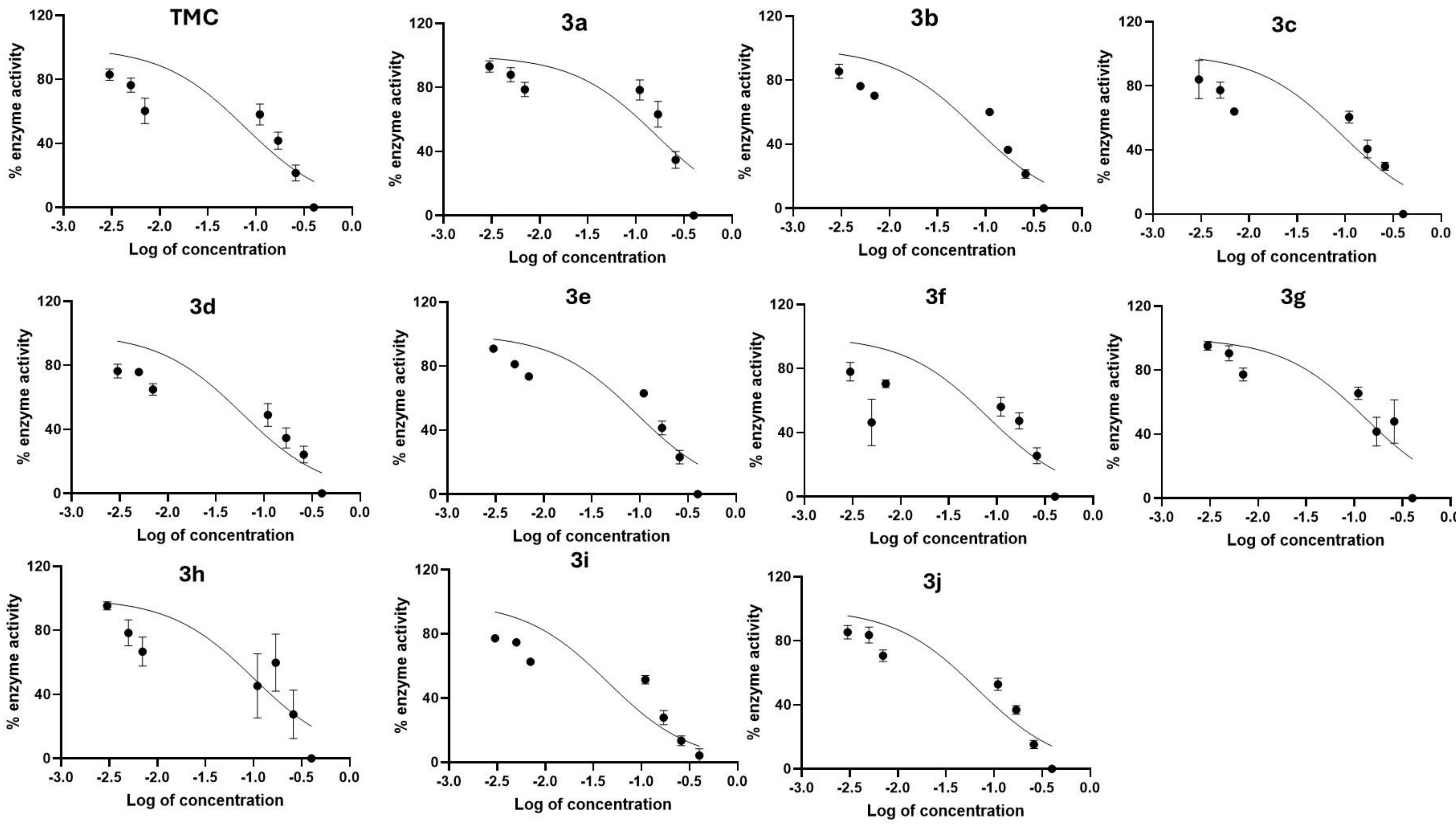

Figure S48. Proteolytic inhibition assays of chalcone derivatives in acetate buffer

Assays were performed using the fluorogenic peptide substrate Z-Phe-Arg-7-amido-4-methylcoumarin (Z-FR-AMC, 1 mM) and promastigote protein extract (5 µg) in 10mM acetate buffer (pH 5.5). The following chalcone compounds were analyzed: **2′,4′,6′-trimethoxychalcone (TMC)**, **pinostrobin chalcone (3a)**, **flavokawain B (3b)**, and derivatives **3c–3j**. The sigmoidal curves indicate the IC50 values (half-maximal inhibitory concentration), representing the dose-response relationship. The distribution of experimental data points (●) shows low inhibition at lower concentrations, a sharp decline in the mid-range, and stabilization at high inhibitory concentrations. Graphs illustrate residual enzymatic activity expressed as a percentage of the control (mean ± SD; %) as a function of the logarithm of the molar concentration (Log of concentration). Data corresponds to three independent experiments

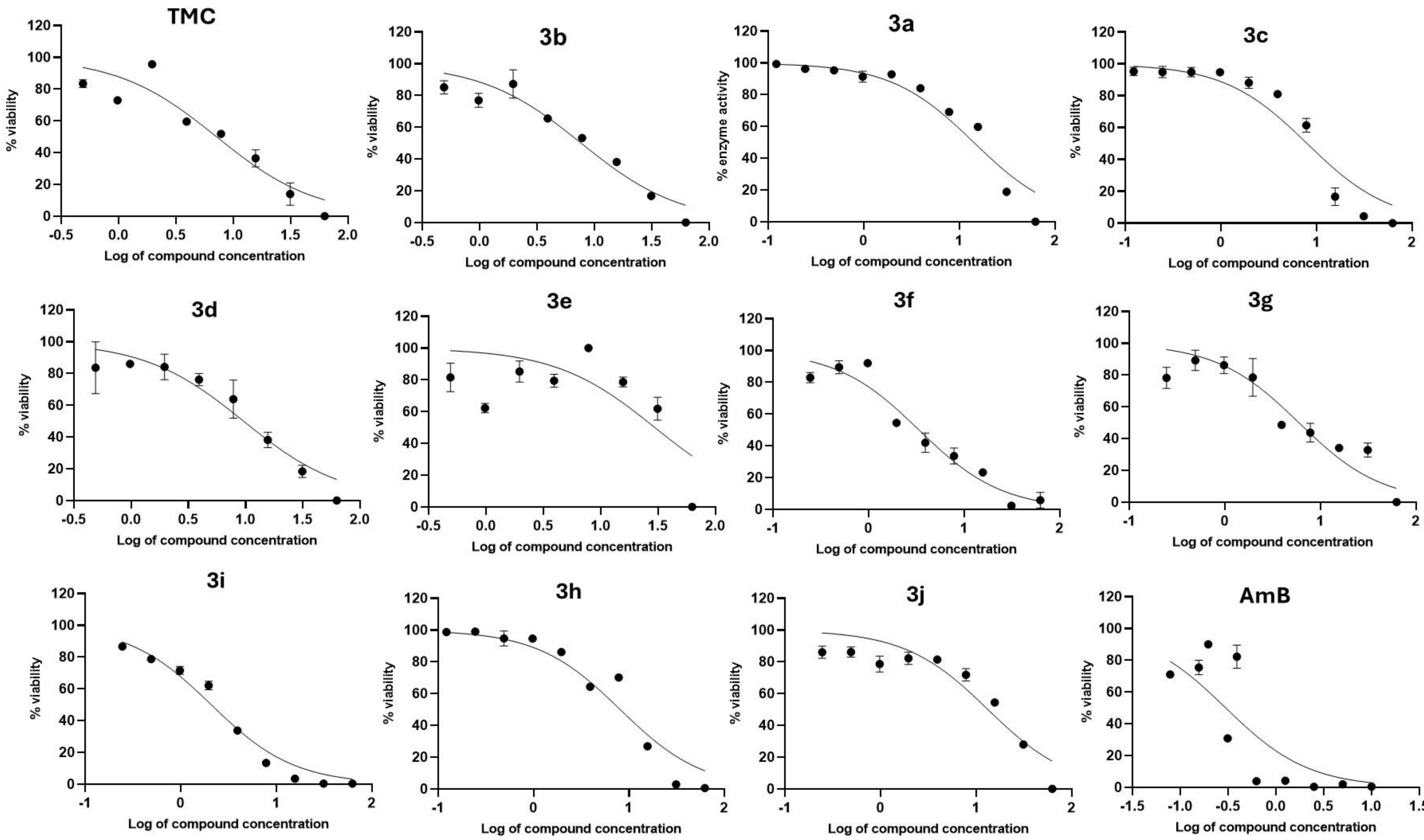

Figure S49. Promastigote viability profiles

Parasites (2x 10^6 per well) were cultured in 96-well plates containing Schneider's medium supplemented with 20% FBS, in a final volume of 100 µL. These assays were performed in the presence or absence of chalcone compounds were analyzed: **2′,4′,6′-trimethoxychalcone (TMC)**, **pinostrobin chalcone (3a)**, **flavokawain B (3b)**, and derivatives **3c–3j**, and amphotericin B (**AmB**). The sigmoidal curves indicate the IC50 values (half-maximal inhibitory concentration), representing the dose-response relationship. The distribution of experimental data points (●) shows low inhibition at lower concentrations, a sharp decline in the mid-range, and stabilization at high inhibitory concentrations. Graphs illustrate residual promastigotes viability expressed as a percentage of the control (mean ± SD; %) as a function of the logarithm of the molar concentration (Log of concentration). Data corresponds to three independent experiments.

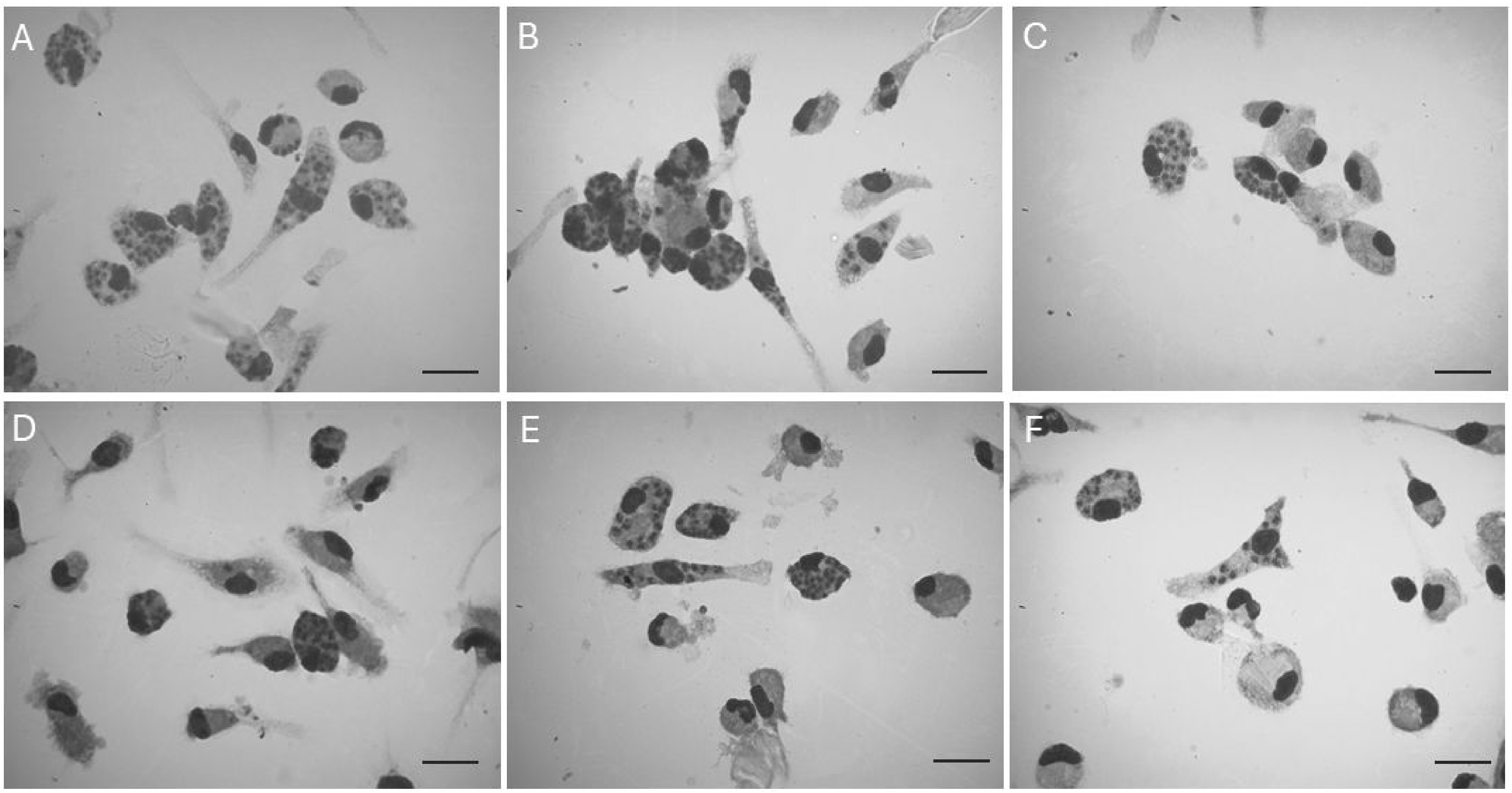

Figure S50. Representative light microscopy images of *Leishmania* (*V*.) *braziliensis*-infected murine macrophages treated with TMC and arylchalcone derivatives.

After infection, macrophages cultured on Lab-Tek chamber slides were treated with 4.0 μM of **TMC** and compounds **3b**, **3d**, **3h**, and **3i** Representative light microscopy images qualitatively illustrate the effects of the treatments on intracellular infection. Untreated macrophages (A) displayed numerous intracellular amastigotes. Compared with the control, **TMC** (B) and compound **3b** (C) showed only a slight apparent reduction in parasite burden, whereas compounds **3d** (D), **3h** (E), and **3i** (F) were associated with fewer visible intracellular amastigotes. These qualitative observations are in agreement with the quantitative analyses presented in the manuscript. Cells were stained using the Panoptic staining method. Scale bar: 50 μm.
